# Diastereodivergent and Enantioselective [4+2] Annulations of γ-Butenolides with Cyclic 1-Azadienes

**DOI:** 10.3390/molecules200813642

**Published:** 2015-07-27

**Authors:** Chao Li, Kun Jiang, Ying-Chun Chen

**Affiliations:** 1 College of Pharmacy, Third Military Medical University, Chongqing 400038, China; E-Mail: lichaolucy@sina.com; 2 Key Laboratory of Drug-Targeting and Drug Delivery System of the Ministry of Education, West China School of Pharmacy, Sichuan University, Chengdu 610041, China

**Keywords:** asymmetric organocatalysis, Brønsted base, cinchona alkaloids, butenolides, 1-azadienes, diastereodivergence

## Abstract

An asymmetric annulation reaction of γ-butenolides and cyclic 1-azadienes containing a 1,2-benzoisothiazole-1,1-dioxide motif has been studied, proceeding in a tandem Michael addition-aza-Michael addition sequence. *Endo*-type cycloadducts bearing fused tetracyclic skeletons were isolated in fair yields and with high enantioselectivity (up to >99% ee) under the catalysis of modified cinchona alkaloid (DHQD)_2_PHAL. Besides, *exo*-type diastereomers could be produced using β-isocupreidine (β-ICD) as the catalyst, though with moderate enantioselectivity.

## 1. Introduction

γ-Butenolides represent an important class of heterocycles, which widely exist in a large number of natural products and pharmaceutically useful molecules [1,2]. In addition, the γ-butenolides act as competent direct vinylogous nucleophiles in addition reactions for the construction of an array of structurally diverse architectures [3,4,5,6], even for preparing more challenging molecules with quaternary carbon centers by using γ-substituted butenolides [7,8,9,10,11,12,13]. In contrast to abundant γ-regioselective vinylogous addition reactions, to the best of our knowledge, no examples have been reported to utilize the γ-butenolides as the reactants towards tandem reactions in consideration of the potential reactivity of both γ- and β-positions [14]. Recently, our group reported a direct asymmetric allylic alkylation of γ-butenolides with MBH carbonates to access γ,γ-disubstituted butenolides that could allow sequential aza-Michael addition to deliver interesting bicyclic piperidine derivatives [15]. These results inspired us to explore domino or tandem Michael addition-aza-Michael addition to construct a variety of polycyclic skeletons.

On the other hand, our group recently developed a series of asymmetric reactions involving cyclic 1-azadienes containing a 1,2-benzoisothiazole-1,1-dioxide motif [16,17,18,19]. They are stable materials, and readily available from diverse saccharins and aldehydes. Importantly, they exhibit high electrophilicity, and can perform as either 2π or 4π partners in Diels-Alder cycloaddition reactions with HOMO-activated enamine species. Therefore, the good reactivity of such 1-azadienes in cycloaddition reactions suggests that they could be applied as potential reactants in the [4+2] reaction with the *in situ* generated dienolates from the γ-butenolides, either in a concerted or stepwise manner [15]. Here, we would like to report the asymmetric assembly of 3-vinyl-1,2-benzoisothiazole-1,1-dioxides and γ-butenolides to furnish chiral tetracyclic molecules with high structural and functional complexity.

## 2. Results and Discussion

### 2.1. The Cycloaddition Reaction of Cyclic 1-Azadienes and γ-Butenolides

#### 2.1.1. Catalyst Screenings for the Cycloaddition Reaction

The initial reaction of styryl-substituted cyclic imine **2a** and commercially available α-angelica lactone **3a** was examined in DCM at 20 °C by applying bifunctional tertiary amine-thiourea **1a** as the catalyst [20]. Unfortunately, the reaction was complicated, and both β,γ-regioselective *endo*-cycloadduct **4a** and *exo*-cycloadduct **5a** were produced in poor yields. In addition, the α-regioselective Michael addition product **6** could also be detected, albeit in much lower yield. Moreover, the enantioselectivity was very disappointing for both diastereomers (Table 1, entry 1). β-isocupreidine (β-ICD) **1b** exhibited excellent *exo*-diastereoselectivity but with moderate enantioselectivity (entry 2) [21]. Poorer results were obtained in the presence of α-isocupreine (α-IC) **1c** (entry 3) [22]. Subsequently, a number of modified cinchona alkaloids **1d**–**1h** were investigated (entries 4–8) [23]. To our gratification, (DHQD)_2_PHAL **1g** provided much better diastereoselectivity, and the enantioselectivity for the major *endo*-cycloadduct **4a** was also good, though the yield was only fair; nevertheless, the ee value of the corresponding *exo*-product was very poor (entry 7).

#### 2.1.2. Studies on *Endo*-Cycloaddition Reaction Catalyzed by (DHQD)_2_PHAL **1g**

In order to further improve the data of *endo*-cycloadduct **4a** by the catalysis of (DHQD)_2_PHAL **1g**, more reaction parameters were investigated. The results are summarized in Table 2. At first, a few solvents were explored at 20 °C (Table 2, entries 1–4), and better diastereoselectivity and excellent enantioselectivity could be obtained in PhCF_3_ (entry 4). The enantioselectivity was decreased at lower or higher reaction temperature (entries 5 and 6). In addition, the yield could not be improved by increasing the catalyst loadings (entry 7) or reaction concentration (entry 8), or by adding 1-azadiene **3a** in portions (entry 9). It should be pointed out that significant amounts of α-regioselective Michael adduct **6** (about 20%) was observed in all the tested reactions, which might account for the fair yield of *endo*-cycloadduct **4a**.

**Table 1:** Initial catalyst screening studies on [4+2] cycloaddition of 3-styryl-1,2-benzoisothiazole-1,1-dioxide **2a** and α-angelica lactone **3a** ^a^. 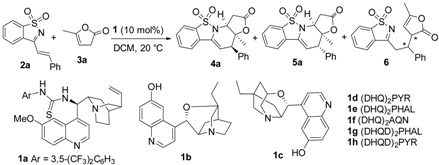

Entry	Cat	t (h)	Yield (%) ^b^ 4a/5a	dr ^c^	ee (%) ^d^ 4a/5a
1	**1a**	24	18/18	1:1	30/10
2	**1b**	24	-/36	1:19	-/55
3	**1c**	48	11/44	1:4	21/36
4	**1d**	48	55/18	3:1	−45/18
5	**1e**	48	37/9	4:1	−85/19
6	**1f**	24	55/23	2:1	10/−35
**7**	**1g**	**24**	**48/5**	**9:1**	**82/7**
8	**1h**	24	67/13	5:1	62/−16

^a^ Reactions were performed with **2a** (0.025 mmol), **3a** (0.05 mmol), **1** (10 mol %) in DCM (0.25 mL) at 20 °C. ^b^ Determined by crude ^1^H-NMR analysis using mesitylene as the internal standard. ^c^ By crude ^1^H-NMR analysis. ^d^ By chiral HPLC analysis. (DHQ)_2_PYR **1d**: hydroquinine-2,5-diphenyl-4,6-pyrimidinediyl diether; (DHQ)_2_PHAL **1e**: hydroquinine 1,4-phthalazinediyl diether; (DHQ)_2_AQN **1f**: hydroquinine (anthrax-quinone-1,4-diyl) diether; (DHQD)_2_PHAL **1g**: hydroquinidine 1,4-phthalazinediyl diether; (DHQD)_2_PYR **1h**: hydroquinidine-2,5-diphenyl-4,6-pyrimidinediyl diether.

**Table 2:** Reaction condition screenings catalyzed by (DHQD)_2_PHAL **1g** ^a^. 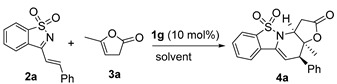

Entry	Solvent	T (°C)	t (h)	Yield (%) ^b^	dr ^c^	ee (%) ^d^
1	DCM	20	24	48	9:1	82)
2	CH_3_CN	20	48	54	5:1	73
3	PhCH_3_	20	48	32	9:1	96
**4**	**PhCF_3_**	**20**	**48**	**44**	**11:1**	**95**
5	PhCF_3_	0	48	28	11:1	90
6	PhCF_3_	50	48	51	7:1	80
7 ^e^	PhCF_3_	20	48	44	11:1	95
8 ^f^	PhCF_3_	20	48	44	11:1	95
9 ^g^	PhCF_3_	20	48	44	11:1	95

^a^ Unless noted otherwise, reactions were performed with **2a** (0.025 mmol), **3a** (0.05 mmol), **1g** (10 mol %) in solvent (0.25 mL). ^b^ Determined by crude ^1^H-NMR analysis using mesitylene as the internal standard. ^c^ By crude ^1^H-NMR analysis. ^d^ By chiral HPLC analysis. ^e^ With 20 mol % of **1g**. ^f^ In 0.125 mL solvent. ^g^ **2a** was added in three portions.

#### 2.1.3. Substrate Scope of *Endo*-Cycloaddition Reaction Catalyzed by (DHQD)_2_PHAL **1g**

With the optimized conditions in hand, we then explored a variety of cyclic 1-azadienes **2** and γ-butenolides **3** under the catalysis of (DHQD)_2_PHAL **1g** in PhCF_3_ at 20 °C. The results are summarized in Table 3. At first, a variety of cyclic 1-azadienes bearing electron-withdrawing or -donating groups on the aryl ring were tested in reactions with α-angelica lactone **3a** (Table 3, entries 1–6). In general, the substrates could be effectively consumed, but the reactions were not clean since some side products were always observed. The desired *endo*-type [4+2] cycloadducts **4** could be smoothly isolated in fair to moderate yields, while high to excellent ee values were generally obtained. In addition, outstanding enantioselectivity was also attained for the cyclic 1-azadienes bearing heteroaryl groups, though the yields were still unsatisfactory (entries 7 and 8). In addition, substitutions on the isothiazole ring had marginal effect on the yields and ee values (entries 9 and 10). On the other hand, other γ-butenolides were further explored in reactions with 1-azadiene **2a**. The similar excellent enantioselectivity along with a fair yield was gained for γ-phenyl-substituted butenolide (entry 11), while the simple 2-butenolide showed poor reactivity, and a moderate ee value was produced (entry 12).

**Table 3:** Substrate scope of *endo*-cycloaddition reaction ^a^. 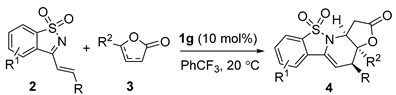

Entry	R	R^1^	R^2^	t (h)	Yield (%) ^b^	ee (%) ^c^
1	Ph	H	CH_3_	48	**4a**, 44	95
2	4-Me-C_6_H_4_	H	CH_3_	36	**4b**, 32	96
3	2-MeO-C_6_H_4_	H	CH_3_	36	**4c**, 43	94
4	3-MeO-C_6_H_4_	H	CH_3_	32	**4d**, 34	>99
5	3,5-(MeO)_2_-C_6_H_3_	H	CH_3_	36	**4e**, 31	94
4	2-F-C_6_H_4_	H	CH_3_	35	**4f**, 30	89
5	3-Br-C_6_H_4_	H	CH_3_	32	**4g**, 57	87
6	4-Br-C_6_H_4_	H	CH_3_	35	**4h**, 42	>99
7	2-Furyl	H	CH_3_	36	**4i**, 29	92
8	2-Thienyl	H	CH_3_	48	**4j**, 40	>99
9	Ph	6-Cl	CH_3_	36	**4k**, 31	97
10	Ph	6-*t*Bu	CH_3_	40	**4l**, 46	98
11	Ph	H	Ph	36	**4m**, 31	94
12	Ph	H	H	120	**4n**, 30	79

^a^ Reactions were performed with **2** (0.3 mmol), **3** (0.6 mmol), and catalyst **1g** (10 mol %) in PhCF_3_ (3 mL) at 20 °C. ^b^ Isolated pure *endo*-product. ^c^ Determined by chiral HPLC analysis.

**Scheme 1:**
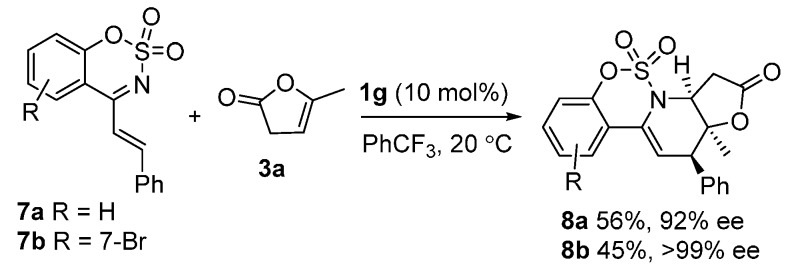
More exploration with other cyclic 1-azadienes.

Moreover, 4-styryl-1,2,3-benzoxathiazine-2,2-dioxides **7** could also be assembled with α-angelica lactone **3a** under the same catalytic conditions, and the corresponding cycloadducts **8** were isolated in excellent enantioselectivity and with modest yields (Scheme 1).

#### 2.1.4. More Studies on the *Exo*-Type Cycloaddition Reaction Catalyzed by β-ICD **1b**

As mentioned above, β-ICD **1b**-catalyzed reaction of 1-azadiene **2a** and α-angelica lactone **3a** dominantly gave *exo*-type cycloadduct **5a** in DCM, thus we explored more reaction conditions with β-ICD **1b**. The results are summarized in Table 4. The similar data were obtained in 1,2-dichloroethane (DCE, Table 4, entry 2), but both diastereo- and enantioselectivity were decreased when other solvents were used (entries 3–6). In addition, changing other types of parameters, such as reaction temperature (entry 7), catalyst loadings (entry 8), and substrate ratio (entry 9), failed to improve the yield and enantioselectivity. As the γ-regioselective vinylogous Michael addition intermediate also was detected in the reaction mixture, tetramethylguanidine (TMG) was added to facilitate the intramolecular aza-Michael addition after the disappearance of substrate **2a**. Pleasingly, better yield for *exo*-**5a** could be obtained without diminishing the stereoselectivity (entry 10). Therefore, the *exo*-cycloadduct seems to be greatly favored by using less hindered Brønsted base as the promoter. Although moderate ee value was obtained, the optical purity of *exo*-cycloadduct **5a** could be improved to 90% ee after a single recrystallization (entry 10, data in parentheses).

**Table 4:** Screening studies on the *exo*-cycloaddition reaction catalyzed by β-ICD **1b** ^a^. 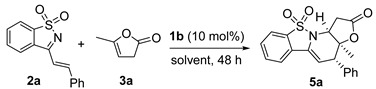

Entry	Solvent	T (°C)	Yield (%) ^b^	dr ^c^	ee (%) ^d^
1	DCM	20	36	>19:1	55
2	DCE	20	34	>19:1	55
3	PhCH_3_	20	30	7:1	44
4	PhCF_3_	20	35	3:1	30
5	Dioxane	20	33	4:1	55
6	CH_3_CN	20	33	3:1	56
7 ^e^	DCM	10	37	>19:1	56
8 ^f^	DCM	20	38	>19:1	55
9 ^g^	DCM	20	37	>19:1	55
10 ^h^	DCM	20	60 (44)	>19:1	55 (90)

^a^ Unless noted otherwise, evaluation reactions were performed with **2a** (0.025 mmol), **3a** (0.05 mmol), and **1b** (10 mol %) in solvent (0.25 mL) for 48 h. ^b^ Determined by ^1^H-NMR analysis using mesitylene as the internal standard. ^c^ By crude ^1^H-NMR analysis. ^d^ Determined by chiral HPLC analysis. ^e^ With 20 mol % of **1b**. ^f^ Three equiv of **3a** was used. ^g^ TMG was added after **2a** was consumed. ^h^ Data in parentheses referred to those after recrystallization.

Consequently, a few cyclic imine **2** were further tested in reactions with α-angelica lactone **3a** under the above optimized conditions. As summarized in Table 5, all the reactions exhibited exclusive *exo*-diastereoselectivity, and the similar moderate enantioselectivity along with fair to modest yields was obtained (Table 5, entries 1–6). Simple 2-butenolide showed good reactivity, delivering the product **5g** in moderate yield and ee value (entry 7).

**Table 5:** Substrate scope of *exo*-type cycloadditions catalyzed by **1b** ^a^. 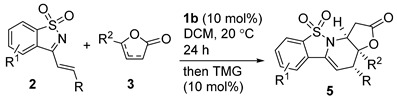

Entry	R	R^1^	R^2^	Yield (%) ^b^	ee (%) ^c^
1	Ph	H	CH_3_	**5a**, 60	55
2	4-Me-C_6_H_4_	H	CH_3_	**5b**, 61	63
3	4-Br-C_6_H_4_	H	CH_3_	**5c**, 47	56
4	2-Thienyl	H	CH_3_	**5d**, 34	63
5	1-Naphthyl	H	CH_3_	**5e**, 44	66
6	Ph	6-Cl	CH_3_	**5f**, 37	55
7	Ph	H	H	**5g**, 58	54

^a^ Reactions were performed with **2** (0.3 mmol), **3** (0.6 mmol), and **1b** (10 mol %), in DCM (3 mL) at 20 °C for 24 h. ^b^ Isolated pure *exo*-product **5**. ^c^ By chiral HPLC analysis.

### 2.2. Absolute Configuration of Endo-**4a** and Exo-**5a**

**Figure 1:**
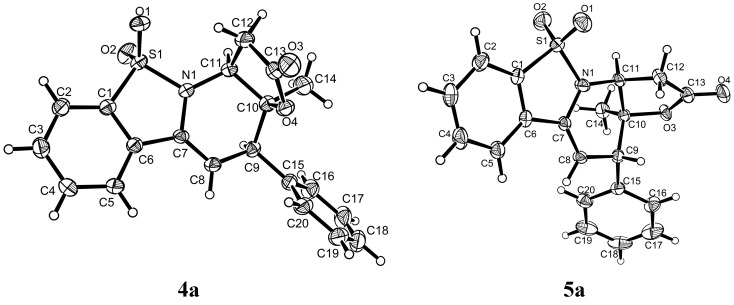
X-ray crystal structures of the cycloadducts **4a** and **5a**.

In order to determine the absolute configuration of the cycloadducts, single crystals suitable for X-ray crystallographic analysis were obtained from product **4a** and **5a**, respectively. Over 99% ees could be obtained after recrystallization from **4a** (95% ee) and **5a** (55% ee) in a mixture of ethyl acetate, isopropanol and petroleum ether. Thus, the absolute configuration of **4a** and **5a** could be unambiguously assigned, as outlined in Figure 1 [24], and more crystal data and structures refinement for 4a and 5a could be found in the supplementary materials.

### 2.3. Derivation of the Cycloaddition Product

**Scheme 2:**
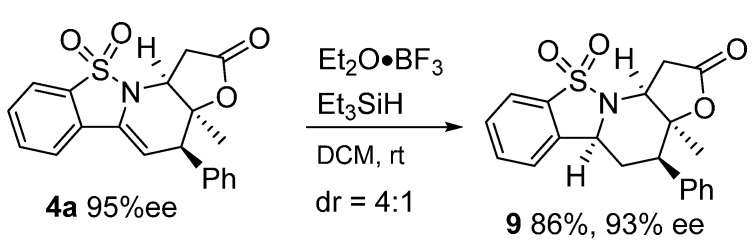
Reduction of cycloaddition product.

The unsaturated cyclic enamide group of **4a** could be reduced by Et_2_O^.^BF_3_ and Et_3_SiH [25], delivering the corresponding product **9** in a good yield and with a moderate diastereoselectivity (Scheme 2).

## 3. Experimental Section

### 3.1. General Methods

NMR data were obtained for ^1^H at 600 MHz and for ^13^C at 151 MHz. Chemical shifts were reported in ppm from tetramethylsilane with the solvent resonance as the internal standard in CDCl_3_ solution. ESI HRMS was recorded on a Waters SYNAPT G2. In each case, enantiomeric ratio was determined by HPLC analysis on a chiral column in comparison with authentic racemate, using a Daicel Chiralcel OD-H Column (250 × 4.6 mm), Chiralcel IA Column (250 × 4.6 mm), Chiralcel IC Column (250 × 4.6 mm), Chiralcel IE Column (250 × 4.6 mm), Chiralcel IF Column (250 × 4.6 mm), or Chiralcel AS-H Column (250 × 4.6 mm). UV detection was monitored at 210 nm or 285 nm. Optical rotation was examined in CH_2_Cl_2_ solution at 25 °C. Column chromatography was performed on silica gel (400 mesh) eluting with ethyl acetate and petroleum ether or DCM. TLC was performed on glass-backed silica plates. UV light and I_2_ were used to visualize products. All chemicals including α-angelica lactone **2a** were used without purification as commercially available unless otherwise noted, and the other butenolides were prepared according to the literatures [15]. α,β-Unsaturated imines **2** and **7** were prepared according to the literature procedures [16]. The tertiary amines **1b** and **1c** were also synthesized according to the literature procedures [21,22], and others were commercial available.

### 3.2. Experimental Procedures

#### 3.2.1. General Procedure for the Preparation of *Endo*-Cycloadduct **4** or **8**

The reaction was carried out with cyclic 1-azadiene **2** or **7** (0.3 mmol) and butenolide **3** (0.6 mmol) in benzotrifluoride (3.0 mL) in the presence of tertiary amine catalyst **1g** (23.4 mg, 0.03 mmol) at 20 °C. After accomplishment, the solution was concentrated and the residue was purified by flash chromatography on silica gel (DCM/ethyl acetate = 150:1) to afford the chiral product **4** or **8**.

#### 3.2.2. General Procedure for the Preparation of *Exo*-Cycloadduct **5**

3-Vinyl-1,2-benzoisothiazole-1,1-dioxide **2** (0.3 mmol) and butenolide **3** (0.6 mmol) were dissolved in DCM (2.0 mL). Then tertiary amine catalyst **1b** (9.3 mg, 0.03 mmol) was added. The solution was stirred at 20 °C for 24 h. After the disappearance of **2**, TMG (3.8 μL, 0.03 mmol) was added and the mixture was stirred for another 12 h. Then the solution was concentrated and the residue was purified by flash chromatography on silica gel (DCM/ethyl acetate = 150:1) to afford the chiral product **5**.

#### 3.2.3. General Procedure for the Preparation of **9**

To a solution of **4a** (37 mg, 0.10 mmol) in DCM (1 mL) was added Et_2_O·BF_3_ (100 μL, 1 mmol) and Et_3_SiH (130 μL, 1 mmol). The solution was stirred at room temperature for 4 h. Purification by column chromatography on silica gel (eluting with DCM/EA = 150:1) to give **9** as a white solid.

*(3aS,4R,11aS)-3a-Methyl-4-phenyl-3a,4-dihydro-1H-benzo[4,5]isothiazolo[2,3-a]furo[2,3-e]pyridin-2(11aH)-one 10,10-dioxide* (**4a**) was obtained in 44% yield; the enantiomeric excess was determined to be 95% by HPLC analysis on Daicel Chiralcel IE column (40% 2-propanol/*n*-hexane, 1 mL/min, temperature 35 °C), UV 285 nm, t_major_ = 23.48 min, t_minor_ = 38.66 min. ${\left[\alpha \right]}_{D}^{25}$ = 71.0 (c = 0.4 M CH_2_Cl_2_); ^1^H-NMR (600 MHz, CDCl_3_) δ: 7.85 (d, *J* = 7.8 Hz, 1H), 7.72–7.67 (m, 2H), 7.63–7.59 (m, 1H), 7.35–7.29 (m, 5H), 5.81 (d, *J* = 4.7 Hz, 1H), 4.55 (t, *J* = 6.3 Hz, 1H), 3.82 (d, *J* = 4.7 Hz, 1H), 2.98 (dd, *J* = 6.1, 5.2 Hz, 2H), 1.61 (s, 3H); ^13^C-NMR (151 MHz, CDCl_3_) δ: 172.71, 136.38, 133.51, 132.42, 131.07, 130.51, 128.94, 128.44, 128.09, 121.28, 101.14, 83.58, 54.95, 47.71, 36.26, 26.78; ESI HRMS: calcd. for C_20_H_17_NO_4_S + Na^+^ 390.0776, found 390.0775.

*(3aS,4R,11aS)-3a-Methyl-4-(p-tolyl)-3a,4-dihydro-1H-benzo[4,5]isothiazolo[2,3-a]furo[2,3-e]pyridine-2(11aH)-one 10,10-dioxide* (**4b**) was obtained in 32% yield; the enantiomeric excess was determined to be 96% by HPLC analysis on Daicel Chiralcel IE column (40% 2-propanol/*n*-hexane, 1 mL/min, temperature 35 °C), UV 285 nm, t_major_ = 26.39 min, t_minor_ = 40.71 min. ${\left[\alpha \right]}_{D}^{25}$ = 136.5 (c = 0.8 M CH_2_Cl_2_); ^1^H-NMR (600 MHz, CDCl_3_) δ: 7.86 (d, *J* = 7.8 Hz, 1H), 7.74–7.67 (m, 2H), 7.62 (t, *J* = 7.4 Hz, 1H), 7.23 (d, *J* = 7.9 Hz, 2H), 7.15 (d, *J* = 7.8 Hz, 2H), 5.81 (d, *J* = 4.8 Hz, 1H), 4.55 (t, *J* = 6.5 Hz, 1H), 3.80 (d, *J* = 4.7 Hz, 1H), 2.97 (d, *J* = 6.3 Hz, 2H), 2.34 (s, 3H), 1.62 (s, 3H); ^13^C-NMR (151 MHz, CDCl_3_) δ: 172.75, 137.88, 133.49, 133.19, 132.39, 130.93, 130.45, 130.36, 129.14, 128.97, 121.27, 101.29, 83.57, 54.87, 47.33, 36.32, 26.87, 21.07; ESI HRMS: calcd. for C_21_H_19_NO_4_S + Na^+^ 404.0932, found 404.0929.

*(3aS,4R,11aS)-4-(2-Methoxyphenyl)-3a-methyl-3a,4-dihydro-1H-benzo[4,5]isothiazolo[2,3-a]furo[2,3-e]pyridin-2(11aH)-one 10,10-dioxide* (**4c**) was obtained in 43% yield; the enantiomeric excess was determined to be 94% by HPLC analysis on Daicel Chiralcel IA column (10% 2-propanol/*n*-hexane, 1 mL/min, temperature 35 °C), UV 285 nm, t_minor_ = 33.00 min, t_major_ = 41.11 min. ${\left[\alpha \right]}_{D}^{25}$ = 19.8 (c = 0.44 M CH_2_Cl_2_); ^1^H-NMR (600 MHz, CDCl_3_) δ: 7.85 (d, *J* = 7.9 Hz, 1H), 7.68 (t, *J* = 7.4 Hz, 2H), 7.61 (d, *J* = 6.6 Hz, 1H), 7.34–7.27 (m, 2H), 6.95 (dd, *J* = 14.1, 7.7 Hz, 2H), 5.71 (d, *J* = 4.2 Hz, 1H), 4.60–4.55 (m, 1H), 4.47 (s, 1H), 3.89 (s, 3H), 3.12 (dd, *J* = 18.1, 5.2 Hz, 1H), 3.01 (dd, *J* = 18.1, 7.1 Hz, 1H), 1.60 (s, 3H); ^13^C-NMR (151 MHz, CDCl_3_) δ: 172.87, 157.77, 133.38, 132.39, 130.71, 130.24, 129.29, 129.16, 125.54, 121.17, 120.67, 110.74, 84.44, 55.63, 55.27, 35.93, 26.23; ESI HRMS: calcd. for C_21_H_19_NO_5_S + Na^+^ 420.0882, found 420.0880.

*(3aS,4R,11aS)-4-(3-Methoxyphenyl)-3a-methyl-3a,4-dihydro-1H-benzo[4,5]isothiazolo[2,3-a]furo [2,3-e]pyridin-2(11aH)-one 10,10-dioxide* (**4d**) was obtained in 34% yield; the enantiomeric excess was determined to be >99% by HPLC analysis on Daicel Chiralcel IA column (10% 2-propanol/*n*-hexane, 1 mL/min, temperature 35 °C), UV 285 nm, t_minor_ = 42.73 min, t_major_ = 50.51 min. ${\left[\alpha \right]}_{D}^{25}$ = 89.9 (c = 0.96 M CH_2_Cl_2_); ^1^H-NMR (600 MHz, CDCl_3_) δ: 7.86 (d, *J* = 7.9 Hz, 1H), 7.74–7.67 (m, 2H), 7.62 (t, *J* = 7.4 Hz, 1H), 7.26 (t, *J* = 3.8 Hz, 1H), 6.93 (d, *J* = 7.6 Hz, 1H), 6.91–6.84 (m, 2H), 5.80 (d, *J* = 4.6 Hz, 1H), 4.56 (t, *J* = 6.2 Hz, 1H), 3.81 (s, 1H), 3.80 (s, 3H), 3.06 (dd, *J* = 18.1, 5.3 Hz, 1H), 2.98 (dd, *J* = 18.1, 7.1 Hz, 1H), 1.62 (s, 3H); ^13^C-NMR (151 MHz, CDCl_3_) δ: 172.69, 159.51, 137.96, 133.47, 132.46, 131.02, 130.48, 129.36, 128.93, 122.87, 121.27, 116.68, 113.11, 101.10, 83.54, 55.00, 47.66, 36.15, 26.77; ESI HRMS: calcd. for C_21_H_19_NO_5_S + Na^+^ 420.0882, found 420.0883.

*(3aS,4R,11aS)-4-(3,5-Dimethoxyphenyl)-3a-methyl-3a,4-dihydro-1H-benzo[4,5]isothiazolo[2,3-a]furo[2,3-e]pyridin-2(11aH)-one 10,10-dioxide* (**4e**) was obtained in 31% yield; the enantiomeric excess was determined to be 94% by HPLC analysis on Daicel Chiralcel IA column (10% 2-propanol/*n*-hexane, 1 mL/min, temperature 35 °C), UV 285 nm, t_minor_ = 47.47 min, t_major_ = 51.77 min. ${\left[\alpha \right]}_{D}^{25}$ = 116.4 (c = 0.28 M CH_2_Cl_2_); ^1^H-NMR (600 MHz, CDCl_3_) δ: 7.85 (d, *J* = 7.8 Hz, 1H), 7.72–7.67 (m, 2H), 7.62 (d, *J* = 7.3 Hz, 1H), 6.49 (d, *J* = 1.9 Hz, 2H), 6.42 (s, 1H), 5.79 (d, *J* = 4.6 Hz, 1H), 4.58–4.54 (m, 1H), 3.78 (s, 6H), 3.75 (d, *J* = 4.5 Hz, 1H), 3.11 (dd, *J* = 18.1, 5.2 Hz, 1H), 2.98 (dd, *J* = 18.1, 7.1 Hz, 1H), 1.62 (s, 3H); ^13^C-NMR (151 MHz, CDCl_3_) δ: 172.73 (s), 160.60 (s), 138.75 (s), 133.46 (s), 130.96 (s), 130.48 (s), 128.92 (s), 121.27 (d, *J* = 11.3 Hz), 108.96 (s), 101.09 (s), 99.55 (s), 83.53 (s), 55.41 (s), 55.08 (s), 47.78 (s), 36.03 (s), 26.70 (s) ppm; ESI HRMS: calcd. for C_21_H_21_NO_6_S + Na^+^ 450.0987, found 450.0984.

*(3aS,4R,11aS)-4-(2-Fluorophenyl)-3a-methyl-3a,4-dihydro-1H-benzo[4,5]isothiazolo[2,3-a]furo[2,3-e]pyridin-2(11aH)-one 10,10-dioxide* (**4f**) was obtained in 30% yield; the enantiomeric excess was determined to be 89% by HPLC analysis on Daicel Chiralcel IE column (40% 2-propanol/*n*-hexane, 1 mL/min, temperature 35 °C), UV 285 nm, t_major_ = 20.02 min, t_minor_ = 33.58 min. ${\left[\alpha \right]}_{D}^{25}$ = 91.3 (c = 0.88 M CH_2_Cl_2_); ^1^H-NMR (600 MHz, CDCl_3_) δ: 7.85 (d, *J* = 7.8 Hz, 1H), 7.69 (q, *J* = 7.8 Hz, 2H), 7.62 (t, *J* = 7.2 Hz, 1H), 7.38 (t, *J* = 7.4 Hz, 1H), 7.32 (dd, *J* = 13.7, 6.9 Hz, 1H), 7.13 (dt, *J* = 18.2, 8.2 Hz, 2H), 5.68 (d, *J* = 4.1 Hz, 1H), 4.62–4.57 (m, 1H), 4.28 (d, *J* = 3.9 Hz, 1H), 3.16 (dd, *J* = 18.2, 4.5 Hz, 1H), 3.05 (dd, *J* = 18.2, 6.9 Hz, 1H), 1.62 (s, 3H); ^13^C-NMR (151 MHz, CDCl_3_) δ: 172.58, 162.01, 160.37, 133.53, 132.43, 131.48, 131.38, 130.56, 129.93, 128.85 124.33, 124.19, 124.10, 121.25, 115.51, 115.36, 100.38, 83.84, 55.16, 39.58, 35.97, 26.08; ESI HRMS: calcd. for C_20_H_16_FNO_4_S + Na^+^ 408.0682, found 408.0678.

*(3aS,4R,11aS)-4-(3-Bromophenyl)-3a-methyl-3a,4-dihydro-1H-benzo[4,5]isothiazolo[2,3-a]furo [2,3-e]pyridin-2(11aH)-one 10,10-dioxide* (**4g**) was obtained in 57% yield; the enantiomeric excess was determined to be 87% by HPLC analysis on Daicel Chiralcel IA column (20% 2-propanol/*n*-hexane, 1 mL/min, temperature 35 °C), UV 285 nm, t_major_ = 22.19 min, t_minor_ = 29.23 min. ${\left[\alpha \right]}_{D}^{25}$ = 72.8 (c = 0.88 M CH_2_Cl_2_); ^1^H-NMR (600 MHz, CDCl_3_) δ: 7.86 (d, *J* = 7.8 Hz, 1H), 7.72 (t, *J* = 8.1 Hz, 2H), 7.66–7.61 (m, 1H), 7.51–7.46 (m, 2H), 7.30 (d, *J* = 7.8 Hz, 1H), 7.24 (t, *J* = 7.8 Hz, 1H), 5.73 (d, *J* = 4.2 Hz, 1H), 4.57 (dd, *J* = 6.6, 4.6 Hz, 1H), 3.78 (d, *J* = 4.2 Hz, 1H), 3.13 (dd, *J* = 18.2, 4.4 Hz, 1H), 3.04 (dd, *J* = 18.2, 6.8 Hz, 1H), 1.60 (s, 3H); ^13^C-NMR (151 MHz, CDCl_3_) δ: 172.51, 139.05, 133.56, 133.28, 132.50, 131.38 , 131.26, 130.64, 129.96, 129.21, 128.78, 122.50, 121.30, 100.45, 83.41, 55.14, 47.34 , 36.11, 26.33; ESI HRMS: calcd. for C_20_H_16_BrNO_4_S + Na^+^ 467.9881, found 467.9882.

*(3aS,4R,11aS)-4-(4-Bromophenyl)-3a-methyl-3a,4-dihydro-1H-benzo[4,5]isothiazolo[2,3-a]furo [2,3-e]pyridin-2(11aH)-one 10,10-dioxide* (**4h**) was obtained in 42% yield; the enantiomeric excess was determined to be >99% by HPLC analysis on Daicel Chiralcel IE column (40% 2-propanol/*n*-hexane, 1 mL/min, temperature 35 °C), UV 285 nm, t_major_ = 21.91 min, t_minor_ = 77.19 min. ${\left[\alpha \right]}_{D}^{25}$ = 100.4 (c = 0.52 M CH_2_Cl_2_); ^1^H-NMR (600 MHz, CDCl_3_) δ: 7.85 (d, *J* = 7.8 Hz, 1H), 7.74–7.67 (m, 2H), 7.63 (dd, *J* = 10.5, 4.0 Hz, 1H), 7.48 (d, *J* = 8.4 Hz, 2H), 7.23 (d, *J* = 8.4 Hz, 2H), 5.74 (d, *J* = 4.4 Hz, 1H), 4.56 (s, 1H), 3.79 (d, *J* = 4.3 Hz, 1H), 3.02 (t, *J* = 6.4 Hz, 2H), 1.59 (s, 3H); ^13^C-NMR (151 MHz, CDCl_3_) δ: 172.51, 135.60, 133.57, 132.47, 132.11, 131.60, 131.36, 130.65, 128.77, 122.26, 121.29, 100.54, 83.38, 55.05, 47.14, 36.19, 26.50; ESI HRMS: calcd. for C_20_H_16_BrNO_4_S + Na^+^ 467.9881, found 467.9885.

*(3aS,4R,11aS)-4-(Furan-2-yl)-3a-methyl-3a,4-dihydro-1H-benzo[4,5]isothiazolo[2,3-a]furo[2,3-e] pyridin-2(11aH)-one 10,10- dioxide* (**4i**) was obtained in 29% yield; the enantiomeric excess was determined to be 92% by HPLC analysis on Daicel Chiralcel IA column (20% 2-propanol/*n*-hexane, 1 mL/min, temperature 35 °C), UV 285 nm, t_major_ = 42.11 min, t_minor_ = 58.68 min. ${\left[a\right]}_{D}^{25}$ = 5.8 (c = 0.12 M CH_2_Cl_2_); ^1^H-NMR (600 MHz, CDCl_3_) δ: 7.87 (d, *J* = 7.8 Hz, 1H), 7.75 (d, *J* = 7.8 Hz, 1H), 7.73–7.69 (m, 1H), 7.64 (dd, *J* = 11.1, 4.0 Hz, 1H), 7.41 (d, *J* = 1.0 Hz, 1H), 6.35 (dd, *J* = 3.1, 1.9 Hz, 1H), 6.28 (d, *J* = 3.2 Hz, 1H), 5.81 (d, *J* = 5.7 Hz, 1H), 4.53 (t, *J* = 8.1 Hz, 1H), 3.97 (d, *J* = 5.7 Hz, 1H), 2.97 (dd, *J* = 18.0, 8.2 Hz, 1H), 2.83 (dd, *J* = 18.0, 8.1 Hz, 1H), 1.69 (s, 3H); ^13^C-NMR (151 MHz, CDCl_3_) δ: 172.21, 149.97, 142.95, 133.52, 132.33, 130.88, 130.72, 128.72, 121.43, 121.33, 110.75, 110.47, 97.16, 82.67, 53.88, 41.71, 35.48, 27.14; ESI HRMS: calcd. for C_18_H_15_NO_5_S + Na^+^ 380.0569, found 380.0567.

*(3aS,4S,11aS)-3a-Methyl-4-(thiophen-2-yl)-3a,4-dihydro-1H-benzo[4,5]isothiazolo[2,3-a]furo[2,3-e]pyridin-2(11aH)-one 10,10-dioxide* (**4j**) was obtained in 40% yield; the enantiomeric excess was determined to be >99% by HPLC analysis on Daicel Chiralcel IA column(10% 2-propanol/*n*-hexane, 1 mL/min, temperature 35 °C), UV 285 nm, t_major_ = 49.55 min, t_minor_ = 68.53 min. ${\left[a\right]}_{D}^{25}$ = 70.5 (c = 0.4 M CH_2_Cl_2_); ^1^H-NMR (600 MHz, CDCl_3_) δ: 7.87 (d, *J* = 7.8 Hz, 1H), 7.76 (d, *J* = 7.8 Hz, 1H), 7.72 (t, *J* = 7.6 Hz, 1H), 7.65 (t, *J* = 7.6 Hz, 1H), 7.28 (d, *J* = 5.1 Hz, 1H), 7.04 (d, *J* = 2.8 Hz, 1H), 7.01–6.99 (m, 1H), 5.93 (d, *J* = 5.3 Hz, 1H), 4.54 (t, *J* = 7.7 Hz, 1H), 4.13 (d, *J* = 5.5 Hz, 1H), 2.94 (dd, *J* = 17.9, 7.9 Hz, 1H), 2.79 (dd, *J* = 17.9, 7.3 Hz, 1H), 1.68 (s, 3H); ^13^C-NMR (151 MHz, CDCl_3_) δ: 172.58, 138.90, 133.56, 132.48, 130.88, 130.76, 128.99, 128.64, 127.22, 126.27, 121.41, 100.21, 82.85, 54.36, 42.84, 36.03, 27.08; ESI HRMS: calcd. for C_18_H_15_NO_4_S_2_ + Na^+^ 396.0340, found 396.0338.

*(3aS,4R,11aS)-8-Chloro-3a-methyl-4-phenyl-3a,4-dihydro-1H-benzo[4,5]isothiazolo[2,3-a]furo [2,3-e]pyridin-2(11aH)-one 10,10-dioxide* (**4k**) was obtained in 31% yield; and the enantiomeric excess was determined to be 97% by HPLC analysis on Daicel Chiralcel ID column (40% 2-propanol/*n*-hexane, 1 mL/min, temperature 35 °C), UV 285nm, t_major_ = 16.55 min, t_minor_ = 21.63 min. ${\left[a\right]}_{D}^{25}$ = 120.5 (c = 0.8 M CH_2_Cl_2_); ^1^H-NMR (600 MHz, CDCl_3_) δ: 7.83 (s, 1H), 7.65 (s, 2H), 7.38–7.31 (m, 5H), 5.80 (d, *J* = 4.5 Hz, 1H), 4.58–4.53 (m, 1H), 3.82 (d, *J* = 4.5 Hz, 1H), 3.01 (dd, *J* = 11.9, 6.1 Hz, 2H), 1.61 (s, 3H) ppm; ^13^C-NMR (151 MHz, CDCl_3_) δ: 172.51, 136.71, 136.23, 133.94, 133.63, 130.46, 130.30, 128.49, 128.17, 127.32, 122.53, 121.41, 101.97, 83.45, 55.14, 47.68, 36.16, 26.58; ESI HRMS: calcd. for C_20_H_16_ClNO_4_S + Na^+^ 424.0386, found 424.0384.

*(3aS,4R,11aS)-8-(tert-Butyl)-3a-methyl-4-phenyl-3a,4-dihydro-1H-benzo[4,5]isothiazolo[2,3-a]furo [2,3-e]pyridin-2(11aH)-one 10,10-dioxide* (**4l**) was obtained in 46% yield; the enantiomeric excess was determined to be 98% by HPLC analysis on Daicel Chiralcel IF column (40% 2-propanol/*n*-hexane, 1 mL/min, temperature 35 °C), UV 285 nm, t_major_ = 9.39 min, t_minor_ = 10.78 min. ${\left[a\right]}_{D}^{25}$ = 78.5 (c = 0.6 M CH_2_Cl_2_); ^1^H-NMR (600 MHz, CDCl_3_) δ: 7.85 (s, 1H), 7.73 (d, *J* = 8.3 Hz, 1H), 7.64 (d, *J* = 8.3 Hz, 1H), 7.36–7.31 (m, 5H), 5.77 (d, *J* = 4.8 Hz, 1H), 4.55 (s, 1H), 3.83 (d, *J* = 4.7 Hz, 1H), 2.96 (d, *J* = 6.6 Hz, 2H), 1.62 (s, 3H), 1.38 (s, 9H); ^13^C-NMR (151 MHz, CDCl_3_) δ: 172.74, 155.05, 136.44, 132.40, 131.32, 131.12, 130.53, 128.40, 128.05, 126.35, 120.98, 117.56, 100.32, 83.65, 54.94, 47.75, 36.30, 35.56, 31.07, 26.95; ESI HRMS: calcd. for C_24_H_25_NO_4_S + Na^+^ 446.1402, found 446.1400.

*(3aR,4R,11aS)-3a,4-Diphenyl-3a,4-dihydro-1H-benzo[4,5]isothiazolo[2,3-a]furo[2,3-e]pyridin-2(11aH)-one 10,10-dioxide* (**4m**) was obtained in 31% yield; and the enantiomeric excess was determined to be 94% by HPLC analysis on Daicel Chiralcel IF column (40% 2-propanol/*n*-hexane, 1 mL/min, temperature 35 °C), UV 285 nm, t_major_ = 10.98 min, t_minor_ = 12.35 min. ${\left[a\right]}_{D}^{25}$ = 22.8 (c = 0.36 M CH_2_Cl_2_); ^1^H-NMR (600 MHz, CDCl_3_) δ: 7.87 (d, *J* = 7.9 Hz, 1H), 7.75–7.69 (m, 2H), 7.63 (t, *J* = 7.5 Hz, 1H), 7.38–7.33 (m, 3H), 7.25–7.22 (m, 3H), 7.18 (t, *J* = 7.4 Hz, 2H), 6.93 (d, *J* = 7.3 Hz, 2H), 5.84 (d, *J* = 3.1 Hz, 1H), 5.03 (d, *J* = 3.8 Hz, 1H), 4.12 (d, *J* = 2.8 Hz, 1H), 3.39 (dd, *J* = 17.6, 1.4 Hz, 1H), 2.65 (dd, *J* = 17.6, 5.0 Hz, 1H); ^13^C-NMR (151 MHz, CDCl_3_) δ: 172.97, 139.70, 136.24, 133.54, 132.50, 131.09, 130.38, 130.23, 129.03, 128.85, 128.78, 127.89, 127.67, 125.25, 121.21, 101.61, 86.25, 56.43, 49.05, 35.65; ESI HRMS: calcd. for C_25_H_19_NO_4_S + Na^+^ 452.0932, found 452.0930.

*(4R,11aS)-4-Phenyl-3a,4-dihydro-1H-benzo[4,5]isothiazolo[2,3-a]furo[2,3-e]pyridin-2(11aH)-one 10,10-dioxide* (**4n**) was obtained in 30% yield; the enantiomeric excess was determined to be 79% by HPLC analysis on Daicel Chiralcel IC column(40% 2-propanol/*n*-hexane, 1 mL/min, temperature 35 °C), UV 285 nm, t_minor_ = 20.60 min, t_major_ = 35.62 min. ${\left[a\right]}_{D}^{25}$ = −12.8 (c = 0.4 M CH_2_Cl_2_); ^1^H-NMR (600 MHz, CDCl_3_) δ: 7.84 (d, *J* = 7.9 Hz, 1H), 7.71 (dd, *J* = 17.3, 7.6 Hz, 2H), 7.61 (t, *J* = 7.4 Hz, 1H), 7.37 (td, *J* = 14.1, 7.1 Hz, 5H), 5.75 (d, *J* = 2.4 Hz, 1H), 4.95 (t, *J* = 4.0 Hz, 1H), 4.81 (s, 1H), 4.10 (d, *J* = 3.6 Hz, 1H), 3.46 (d, *J* = 17.5 Hz, 1H), 2.86 (dd, *J* = 17.5, 5.1 Hz, 1H); ^13^C-NMR (151 MHz, CDCl_3_) δ: 172.99, 137.45, 133.51, 132.25, 131.72, 130.48, 129.19, 129.05, 128.72, 127.95, 121.17, 99.76, 78.12, 51.24, 40.94, 36.76; ESI HRMS: calcd. for C_19_H_15_NO_4_S + Na^+^ 376.0619, found 376.0616.

*(3aS,4S,11aS)-3a-Methyl-4-phenyl-3a,4-dihydro-1H-benzo[4,5]isothiazolo[2,3-a]furo[2,3-e] pyridine-2(11aH)-one 10,10-dioxide* (**5a**) was obtained in 60% yield; the enantiomeric excess was determined to be 55% by HPLC analysis on Daicel Chiralcel IE column (40% 2-propanol/*n*-hexane, 1 mL/min, temperature 35 °C), UV 285 nm, t_major_ = 20.97 min, t_minor_ = 28.28 min. ${\left[a\right]}_{D}^{25}$ = −27.6 (c = 0.84 M CH_2_Cl_2_); ^1^H-NMR (600 MHz, CDCl_3_) δ: 7.87 (d, *J* = 7.8 Hz, 1H), 7.75 (d, *J* = 7.8 Hz, 1H), 7.71 (t, *J* = 7.5 Hz, 1H), 7.64 (t, *J* = 7.5 Hz, 1H), 7.39 (t, *J* = 7.2 Hz, 2H), 7.35 (t, *J* = 7.1 Hz, 1H), 7.28 (d, *J* = 7.2 Hz, 2H), 5.80 (d, *J* = 3.1 Hz, 1H), 4.55 (t, *J* = 8.1 Hz, 1H), 3.93 (d, *J* = 2.9 Hz, 1H), 3.25 (dd, *J* = 17.7, 7.4 Hz, 1H), 3.04 (dd, *J* = 17.7, 8.9 Hz, 1H), 1.19 (s, 3H); ^13^C-NMR (151 MHz, CDCl_3_) δ: 172.4, 137.4, 133.5, 132.2, 130.5, 129.9, 129.4, 128.7, 128.0, 127.96, 121.3, 99.5, 82.9, 53.3, 46.2, 36.5, 21.8; ESI HRMS: calcd. for C_20_H_17_NO_4_S + Na^+^ 390.0776, found 390.0775.

*(3aS,4S,11aS)-3a-Methyl-4-(p-tolyl)-3a,4-dihydro-1H-benzo[4,5]isothiazolo[2,3-a]furo[2,3-e] pyridin-2(11aH)-one 10,10-dioxide* (**5b**) was obtained in 61% yield; the enantiomeric excess was determined to be 63% by HPLC analysis on Daicel Chiralcel IE column (40% 2-propanol/*n*-hexane, 1 mL/min, temperature 35 °C), UV 285 nm, t_major_ = 21.40 min, t_minor_ = 30.40 min. ${\left[a\right]}_{D}^{25}$ = 73.4 (c = 0.80 M CH_2_Cl_2_); ^1^H-NMR (600 MHz, CDCl_3_) δ: 7.86 (d, *J* = 7.9 Hz, 1H), 7.74 (d, *J* = 7.8 Hz, 1H), 7.70 (d, *J* = 7.3 Hz, 1H), 7.63 (d, *J* = 7.5 Hz, 1H), 7.19 (d, *J* = 8.0 Hz, 2H), 7.16 (d, *J* = 8.1 Hz, 2H), 5.78 (d, *J* = 3.1 Hz, 1H), 4.53 (t, *J* = 8.1 Hz, 1H), 3.88 (d, *J* = 3.1 Hz, 1H), 3.22 (dd, *J* = 17.7, 7.4 Hz, 1H), 3.02 (ddd, *J* = 17.7, 8.6, 2.7 Hz, 1H), 2.36 (s, 3H), 1.18 (s, 3H); ^13^C-NMR (151 MHz, CDCl_3_) δ: 172.46, 137.72, 134.33, 133.89, 132.17, 130.47, 129.80, 129.36, 129.28, 128.71, 121.35, 121.28, 99.87, 82.97, 53.27, 45.76, 36.46, 21.93, 21.05; ESI HRMS: calcd. for C_21_H_19_NO_4_S + Na^+^ 404.0932, found 404.0929.

*(3aS,4S,11aS)-4-(4-Bromophenyl)-3a-methyl-3a,4-dihydro-1H-benzo[4,5]isothiazolo[2,3-a]furo [2,3-e]pyridin-2(11aH)-one 10,10-dioxide* (**5c**) was obtained in 47% yield; the enantiomeric excess was determined to be 56% by HPLC analysis on Daicel Chiralcel IE column (40% 2-propanol/*n*-hexane, 1 mL/min, temperature 35 °C), UV 285 nm, t_major_ = 23.11 min, t_minor_ = 36.52 min. ${\left[a\right]}_{D}^{25}$ = 21.3 (c = 1.60 M CH_2_Cl_2_); ^1^H-NMR (600 MHz, CDCl_3_) δ: 7.88 (d, *J* = 7.8 Hz, 1H), 7.76–7.70 (m, 2H), 7.65 (t, *J* = 7.5 Hz, 1H), 7.52 (d, *J* = 8.3 Hz, 2H), 7.17 (d, *J* = 8.3 Hz, 2H), 5.72 (d, *J* = 3.0 Hz, 1H), 4.57–4.49 (m, 1H), 3.89 (d, *J* = 2.9 Hz, 1H), 3.25 (dd, *J* = 17.8, 7.6 Hz, 1H), 2.99 (dd, *J* = 17.8, 9.2 Hz, 1H), 1.18 (s, 3H); ^13^C-NMR (151 MHz, CDCl_3_) δ: 172.11, 136.36, 133.53, 132.23, 131.82, 131.06, 130.69, 130.28, 128.45, 122.14, 121.38, 98.53, 82.40, 53.27, 45.75, 36.36, 21.81; ESI HRMS: calcd. for C_20_H_16_BrNO_4_S + Na^+^ 467.9881, found 467.9883.

*(3aS,4R,11aS)-3a-Methyl-4-(thiophen-2-yl)-3a,4-dihydro-1H-benzo[4,5]isothiazolo[2,3-a]furo[2,3-e]pyridin-2(11aH)-one 10,10-dioxide* (**5d**) was obtained in 34% yield; the enantiomeric excess was determined to be 63% by HPLC analysis on Daicel Chiralcel IA column (10% 2-propanol/*n*-hexane, 1 mL/min, temperature 35 °C), UV 285 nm, t_minor_ = 57.94 min, t_major_ = 73.67 min. ${\left[a\right]}_{D}^{25}$ = 32.0 (c = 0.92 M CH_2_Cl_2_); ^1^H-NMR (600 MHz, CDCl_3_) δ: 7.86 (d, *J* = 7.8 Hz, 1H), 7.76 (d, *J* = 7.8 Hz, 1H), 7.71 (t, *J* = 7.5 Hz, 1H), 7.64 (t, *J* = 7.5 Hz, 1H), 7.30 (dd, *J* = 4.8, 1.0 Hz, 1H), 7.07–7.03 (m, 2H), 5.84 (d, *J* = 3.2 Hz, 1H), 4.57 (dd, *J* = 8.5, 7.8 Hz, 1H), 4.22 (d, *J* = 3.0 Hz, 1H), 3.24 (dd, *J* = 17.8, 7.5 Hz, 1H), 3.00 (dd, *J* = 17.8, 8.9 Hz, 1H), 1.27 (s, 3H); ^13^C-NMR (151 MHz, CDCl_3_) δ: 172.14, 139.78, 133.52, 132.29, 130.69, 129.99, 128.47, 127.18, 121.41, 98.83, 82.82, 53.22, 41.82, 36.48, 21.82; ESI HRMS: calcd. for C_18_H_15_NO_4_S_2_ + Na^+^ 396.0340, found 396.0338.

*(3aS,4S,11aS)-3a-Methyl-4-(naphthalen-1-yl)-3a,4-dihydro-1H-benzo[4,5]isothiazolo[2,3-a]furo [2,3-e]pyridin-2(11aH)-one 10,10-dioxide* (**5e**) was obtained in 44% yield; the enantiomeric excess was determined to be 66% by HPLC analysis on Daicel Chiralcel IF column (40% 2-propanol/*n*-hexane, 1 mL/min, temperature 35 °C), UV 285 nm, t_major_ = 20.51 min, t_minor_ = 34.12 min. ${\left[a\right]}_{D}^{25}$ = 23.5 (c = 1.04 M CH_2_Cl_2_); ^1^H-NMR (600 MHz, CDCl_3_) δ: 8.15 (d, *J* = 8.5 Hz, 1H), 7.93–7.88 (m, 2H), 7.85 (d, *J* = 8.1 Hz, 1H), 7.74–7.67 (m, 2H), 7.63 (dd, *J* = 15.3, 7.7 Hz, 2H), 7.54 (t, *J* = 7.3 Hz, 1H), 7.48 (t, *J* = 7.7 Hz, 1H), 7.34 (d, *J* = 7.0 Hz, 1H), 5.81 (d, *J* = 2.9 Hz, 1H), 4.93 (s, 1H), 4.60 (t, *J* = 6.8 Hz, 1H), 3.29 (dd, *J* = 22.4, 6.8 Hz, 2H), 1.22 (s, 3H); ^13^C-NMR (151 MHz, CDCl_3_) δ: 172.56, 134.84, 133.97, 133.50, 132.21, 130.46, 129.93, 129.02, 128.89, 128.76, 127.39, 126.85, 126.11, 125.13, 123.34, 121.31, 101.64, 84.32, 53.78, 40.23, 36.23, 22.80; ESI HRMS: calcd. for C_24_H_19_NO_4_S + Na^+^ 440.0932, found 440.0928.

*(3aS,4S,11aS)-8-Chloro-3a-methyl-4-phenyl-3a,4-dihydro-1H-benzo[4,5]isothiazolo[2,3-a]furo[2,3-e]pyridin-2(11aH)-one 10,10-dioxide* (**5f**) was obtained in 37% yield; the enantiomeric excess was determined to be 55% by HPLC analysis on Daicel Chiralcel ID column (40% 2-propanol/*n*-hexane, 1 mL/min, temperature 35 °C), UV 285 nm, t_minor_ = 17.39 min, t_major_ = 22.99 min. ${\left[a\right]}_{D}^{25}$ = 35.9 (c = 1.16 M CH_2_Cl_2_); ^1^H-NMR (600 MHz, CDCl_3_) δ: 7.84 (d, *J* = 1.1 Hz, 1H), 7.69–7.63 (m, 2H), 7.42–7.33 (m, 3H), 7.28–7.24 (m, 2H), 5.80 (d, *J* = 3.3 Hz, 1H), 4.53 (t, *J* = 8.0 Hz, 1H), 3.91 (d, *J* = 3.0 Hz, 1H), 3.23 (dd, *J* = 17.8, 7.4 Hz, 1H), 3.04 (dd, *J* = 17.7, 8.6 Hz, 1H), 1.19 (s, 3H); ^13^C-NMR (151 MHz, CDCl_3_) δ: 172.16, 137.19, 136.75, 133.89, 133.40, 129.38, 129.18, 128.74, 128.05, 127.06, 122.61, 121.48, 100.35, 82.74, 53.40, 46.18, 36.39, 22.04; ESI HRMS: calcd. for C_20_H_16_ClNO_4_S + Na^+^ 424.0386, found 424.0384.

*(4S,11aS)-4-Phenyl-3a,4-dihydro-1H-benzo[4,5]isothiazolo[2,3-a]furo[2,3-e]pyridin-2(11aH)-one 10,10-dioxide* (**5g**) was obtained in 58% yield; the enantiomeric excess was determined to be 54% by HPLC analysis on Daicel Chiralcel IE column (40% 2-propanol/*n*-hexane, 1 mL/min, temperature 35 °C), UV 285 nm, t_minor_ = 29.40 min, t_major_ = 51.53 min. ${\left[a\right]}_{D}^{25}$ = −17.5 (c = 0.72 M CH_2_Cl_2_); ^1^H-NMR (600 MHz, CDCl_3_) δ: 7.86 (d, *J* = 7.9 Hz, 1H), 7.70 (dt, *J* = 15.9, 7.7 Hz, 2H), 7.62 (dd, *J* = 10.8, 3.9 Hz, 1H), 7.38 (t, *J* = 7.4 Hz, 2H), 7.33 (t, *J* = 7.3 Hz, 1H), 7.25 (d, *J* = 7.3 Hz, 2H), 5.74 (d, *J* = 4.3 Hz, 1H), 4.70 (t, *J* = 4.1 Hz, 1H), 4.64 (dd, *J* = 8.3, 5.1 Hz, 1H), 4.01 (t, *J* = 3.9 Hz, 1H), 3.42 (dd, *J* = 17.5, 3.1 Hz, 1H), 2.93 (dd, *J* = 17.6, 5.5 Hz, 1H); ^13^C-NMR (151 MHz, CDCl_3_) δ: 172.86, 139.35, 133.56, 132.26, 130.95, 130.54, 129.30, 129.02, 128.13, 121.24, 99.18, 80.44, 48.17, 41.21, 36.38; ESI HRMS: calcd. for C_19_H_15_NO_4_S + Na^+^ 376.0619, found 376.0617.

*3-(2-(1,1-Dioxidobenzo[d]isothiazol-3-yl)-1-phenylethyl)-5-methylfuran-2(3H)-one* (**6**). ^1^H-NMR (600 MHz, CDCl_3_) δ: 7.86 (t, *J* = 8.5 Hz, 1H), 7.71 (s, 2H), 7.64 (dd, *J* = 6.7, 4.5 Hz, 1H), 7.38 (t, *J* = 7.4 Hz, 1H), 7.29 (t, *J* = 7.9 Hz, 2H), 7.05 (d, *J* = 7.6 Hz, 2H), 6.21 (d, *J* = 6.5 Hz, 1H), 4.05 (t, *J* = 7.1 Hz, 1H), 3.77 (dd, *J* = 12.8, 7.7 Hz, 1H), 3.07 (dd, *J* = 18.5, 3.6 Hz, 1H), 2.21–2.17 (m, 1H), 2.11 (s, 3H); ^13^C-NMR (151 MHz, CDCl_3_) δ: 167.35, 137.45, 134.02, 130.99, 129.44, 129.31, 128.26, 128.02, 127.92, 121.75, 106.73, 45.03, 42.37, 39.43, 30.29. ESI LRMS: calcd. for C_20_H_17_NO_4_S + H^+^ 368.1, found 368.

*(3aS,12R,12aS)-12a-Methyl-12-phenyl-3,3a,12,12a-tetrahydro-2H-benzo[e]furo[2',3':5,6]pyrido [1,2-c][1,2,3]oxathiazin-2-one 5,5-dioxide* (**8a**) was obtained in 56% yield; the enantiomeric excess was determined to be 92% by HPLC analysis on Daicel Chiralcel IA column (40% 2-propanol/*n*-hexane, 1 mL/min, temperature 35 °C), UV 285 nm, t_major_ = 5.40 min, t_minor_ = 8.00 min. ${\left[a\right]}_{D}^{25}$ = − 2.7 (c = 1.2 M CH_2_Cl_2_); ^1^H-NMR (600 MHz, CDCl_3_) δ: 7.60 (d, *J* = 7.9 Hz, 1H), 7.44–7.36 (m, 6H), 7.26 (dd, *J* = 8.9, 6.4 Hz, 1H), 7.14 (d, *J* = 8.3 Hz, 1H), 6.24 (d, *J* = 3.9 Hz, 1H), 4.68 (dd, *J* = 8.5, 1.7 Hz, 1H), 3.58 (d, *J* = 3.9 Hz, 1H), 3.14 (dd, *J* = 19.3, 8.5 Hz, 1H), 2.80 (dd, *J* = 19.3, 1.7 Hz, 1H), 1.40 (s, 3H); ^13^C-NMR (151 MHz, CDCl_3_) δ: 173.19, 148.77, 136.83, 133.29, 131.14, 130.07, 128.69, 127.95, 126.46, 124.19, 119.52, 117.65, 115.56, 92.17, 62.29, 49.41, 34.60, 25.14; ESI HRMS: calcd. for C_20_H_17_NO_5_S + Na^+^ 406.0725, found 406.0723.

*(3aS,12R,12aS)-8-Bromo-12a-methyl-12-phenyl-3,3a,12,12a-tetrahydro-2H-benzo[e]furo[2ʹ,3ʹ:5,6] pyrido[1,2-c][1,2,3]oxathiazin-2-one 5,5-dioxide* (**8b**) was obtained in 45% yield; the enantiomeric excess was determined to be >99% by HPLC analysis on Daicel Chiralcel IA column (40% 2-propanol/*n*-hexane, 1 mL/min, temperature 35 °C), UV 285 nm, t_major_ = 6.28 min, t_minor_ = 8.00 min. ${\left[a\right]}_{D}^{25}$ = 6.4 (c = 1.6 M CH_2_Cl_2_); ^1^H-NMR (600 MHz, CDCl_3_) δ: 7.46 (d, *J* = 8.6 Hz, 1H), 7.42–7.35 (m, 6H), 7.32 (d, *J* = 1.5 Hz, 1H), 6.24 (d, *J* = 3.9 Hz, 1H), 4.67 (dd, *J* = 8.4, 1.4 Hz, 1H), 3.55 (d, *J* = 3.9 Hz, 1H), 3.14 (dd, *J* = 19.3, 8.5 Hz, 1H), 2.76 (dd, *J* = 19.3, 1.5 Hz, 1H), 1.39 (s, 3H); ^13^C-NMR (151 MHz, CDCl_3_) δ: 172.98, 148.76, 136.60, 132.56, 130.01, 129.80, 128.74, 128.04, 125.23, 124.08, 122.81, 116.69, 116.30, 92.02, 62.36, 49.43, 34.60, 25.06; ESI HRMS: calcd. for C_20_H_16_BrNO_5_S + Na^+^ 483.9830, found 483.9827.

*(3aS,4R,5aS,11aS)-3a-Methyl-4-phenyl-1,3a,4,5,5a,11a-hexahydro-2H-benzo[4,5]isothiazolo[2,3-a]furo[2,3-e]pyridin-2-one 10,10-dioxide* (**9**) was obtained in 86% yield; the diastereomer ratio was determined to be 4:1 by ^1^H-NMR analysis and the enantiomeric excess was determined to be 93% by HPLC analysis on Daicel Chiralcel IA column (20% 2-propanol/*n*-hexane, 1 mL/min, temperature 35 °C), UV 210 nm, t_major_ = 17.07 min, t_minor_ = 25.06; ${\left[a\right]}_{D}^{25}$ = −32.5 (c = 0.36 M CH_2_Cl_2_); ^1^H-NMR (600 MHz, CDCl_3_) δ: 7.80 (d, *J* = 7.8 Hz, 1H), 7.63 (t, *J* = 7.6 Hz, 1H), 7.55 (t, *J* = 7.5 Hz, 1H), 7.39–7.31 (m, 6H), 4.34 (d, *J* = 11.3 Hz, 1H), 4.19 (d, *J* = 4.7 Hz, 1H), 3.79 (d, *J* = 18.0 Hz, 1H), 2.98 (ddd, *J* = 16.6, 15.5, 4.3 Hz, 2H), 2.47–2.42 (m, 1H), 2.30–2.22 (m, 1H), 1.34 (s, 3H); ^13^C-NMR (151 MHz, CDCl_3_) δ: 173.56, 138.32, 136.22, 135.09, 133.30, 129.68, 129.52, 128.53, 127.86, 122.46, 121.25, 83.36, 59.21, 58.31, 50.52, 34.65, 31.04, 23.49; ESI HRMS: calcd. for C_20_H_19_NO_4_S + Na^+^ 392.0932, found 392.0930.

## 4. Conclusions

We have investigated the asymmetric and β,γ-regioselective [4+2] annulation reactions of γ-butenolides and cyclic 1-azadienes containing a 1,2-benzoisothiazole-1,1-dioxide motif. These reactions occurred in a cascade Michael addition-aza-Michael addition sequence to give complex fused tetracyclic architectures. Diastereodivergent cycloadducts could be produced by employing different Brønsted base catalysts. *Endo*-type cycloadducts were obtained in high enantioselectivity (up to >99% ee) under the catalysis of modified cinchona alkaloid (DHQD)_2_PHAL. On the other hand, *exo*-type diastereomers could be produced catalyzed by β-isocupreidine (β-ICD) followed by TMG-promoted cyclization process, though with moderate enantioselectivity. The potential application of such natural product-like compounds in biological studies is in exploration.

## Supplementary Materials

Supplementary materials can be accessed at: http://www.mdpi.com/1420-3049/20/08/13642/s1.
