# Supplementary material for: Diastereodivergent and Enantioselective [4+2] Annulations of γ-Butenolides with Cyclic 1-Azadienes
Source: Molecules. 2015 Jul 27;20(8):13642–58. doi: 10.3390/molecules200813642 (PMC6332167; doi:10.3390/molecules200813642)
Supplement: Supplementary file 1 [file molecules-20-13642-s001.pdf]

Supplementary Materials

1. Crystal Data and Structures Refinement for Enantiopure 4a and 5a

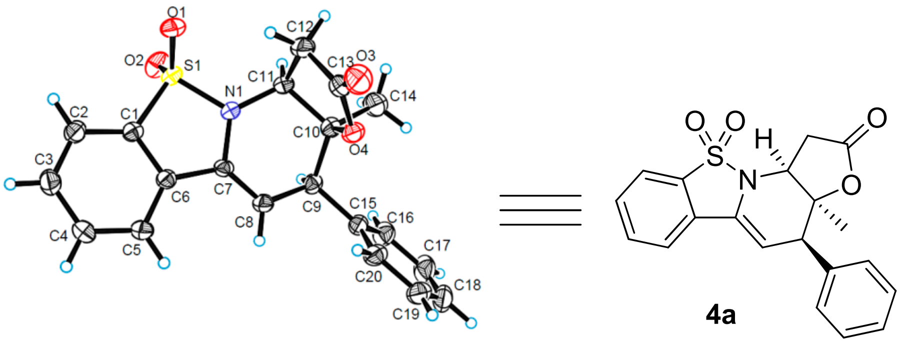

Figure 1. X-ray structure of enantiopure **4a**.

Table 1. Crystal data of enantiopure **4a**.

Identification code	**4a**
Empirical formula	$C_{20}H_{17}NO_4S$
Formula weight	367.40
Temperature/K	290(2)
Crystal system	orthorhombic
Space group	$P2_12_12_1$
a/Å	10.0540(3)
b/Å	12.0824(5)
c/Å	14.5627(4)
α/°	90
β/°	90
γ/°	90
Volume/Å^3	1769.03(10)
Z	4
ρ_{calc}g/cm^3	1.379
μ/mm^{-1}	0.209
F(000)	768.0
Crystal size/mm^3	0.42 × 0.32 × 0.3
Radiation	MoKα (λ = 0.71073)
2Θ range for data collection/°	6.534 to 52.732
Index ranges	$-9 \leq h \leq 12$, $-9 \leq k \leq 15$, $-18 \leq l \leq 18$
Reflections collected	9168
Independent reflections	3620 [R_{int} = 0.0210, R_{sigma} = 0.0289]
Data/restraints/parameters	3620/0/235
Goodness-of-fit on F^2	1.065
Final R indexes [I>=2σ (I)]	R_1 = 0.0369, wR_2 = 0.0840
Final R indexes [all data]	R_1 = 0.0446, wR_2 = 0.0886
Largest diff. peak/hole / e Å^{-3}	0.14/−0.31
Flack parameter	−0.05(3)

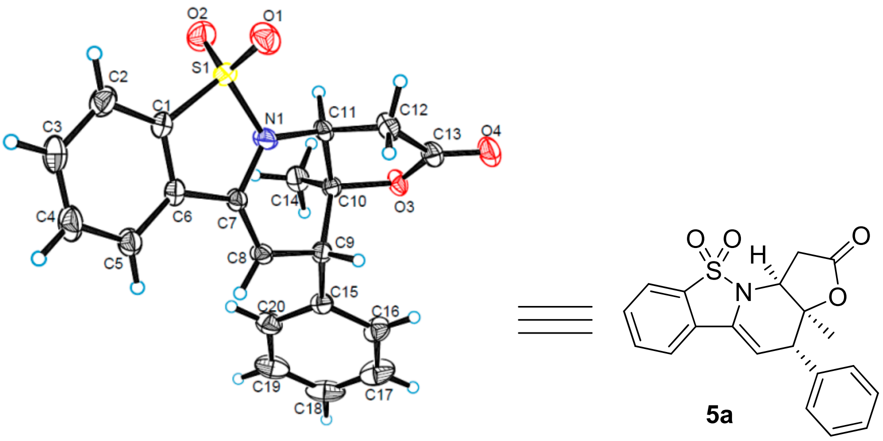

Figure 2. X-ray structure of enantiopure **5a**.

Table 2. Crystal data of enantiopure **5a**.

Identification code	**5a**
Empirical formula	$C_{20}H_{17}NO_4S$
Formula weight	367.40
Temperature/K	291(2)
Crystal system	monoclinic
Space group	$P2_1$
a/Å	8.3275(5)
b/Å	6.8178(4)
c/Å	15.4042(8)
α/°	90
β/°	95.886(5)
γ/°	90
Volume/Å^3	869.97(8)
Z	2
ρ_{calc}g/cm^3	1.403
μ/mm^{-1}	0.212
F(000)	384.0
Crystal size/mm^3	0.32 × 0.3 × 0.25
Radiation	MoKα (λ = 0.71073)
2Θ range for data collection/°	6.54 to 52.744
Index ranges	$-10 \leq h \leq 10, -8 \leq k \leq 8, -19 \leq l \leq 19$
Reflections collected	8527
Independent reflections	3483 [R_{int} = 0.0265, R_{sigma} = 0.0295]
Data/restraints/parameters	3483/1/235
Goodness-of-fit on F^2	1.060
Final R indexes [I>=2σ (I)]	R_1 = 0.0709, wR_2 = 0.1873
Final R indexes [all data]	R_1 = 0.0730, wR_2 = 0.1886
Largest diff. peak/hole/e Å^{-3}	0.90/−0.32
Flack parameter	0.08(6)

2. NMR Spectra and HPLC Chromatograms

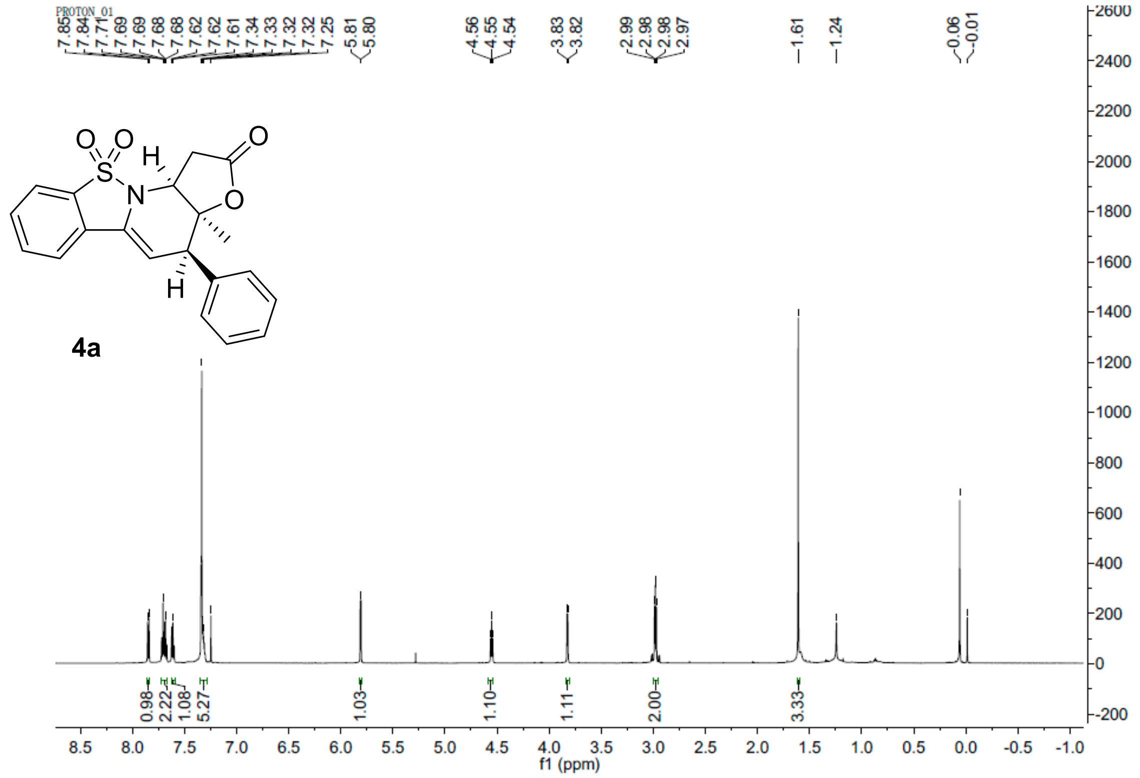

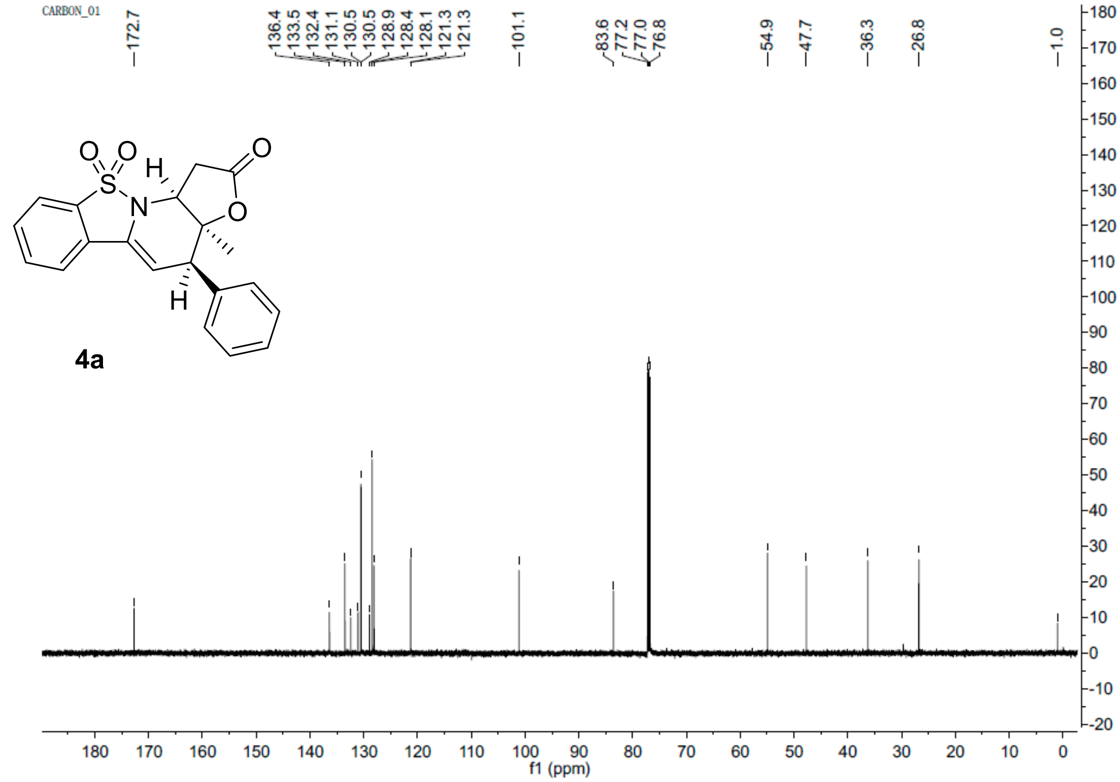

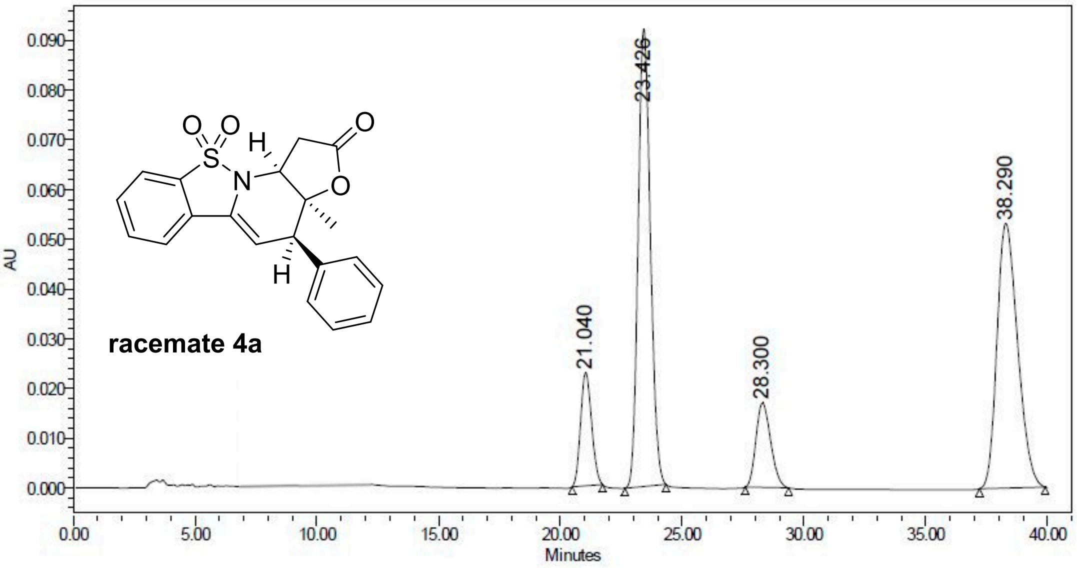

	RT	Area	% Area	Height
1	21.040	715013	9.18	22797
2	23.426	3212990	41.27	92060
3	28.300	716262	9.20	16994
4	38.290	3141649	40.35	53265

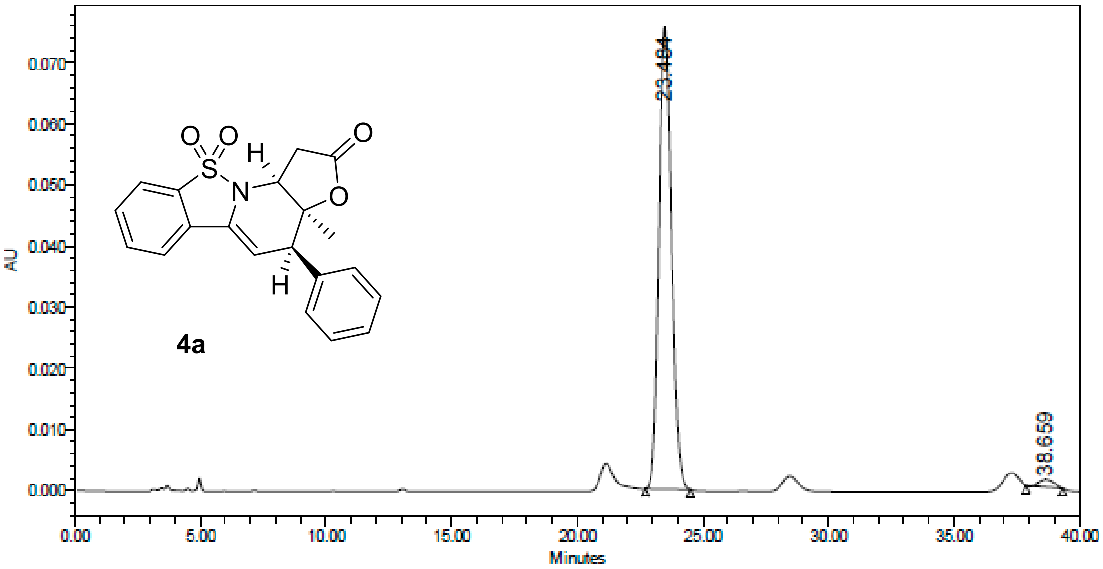

	RT	Area	% Area	Height
1	23.484	2635687	98.09	75481
2	38.659	51335	1.91	1217

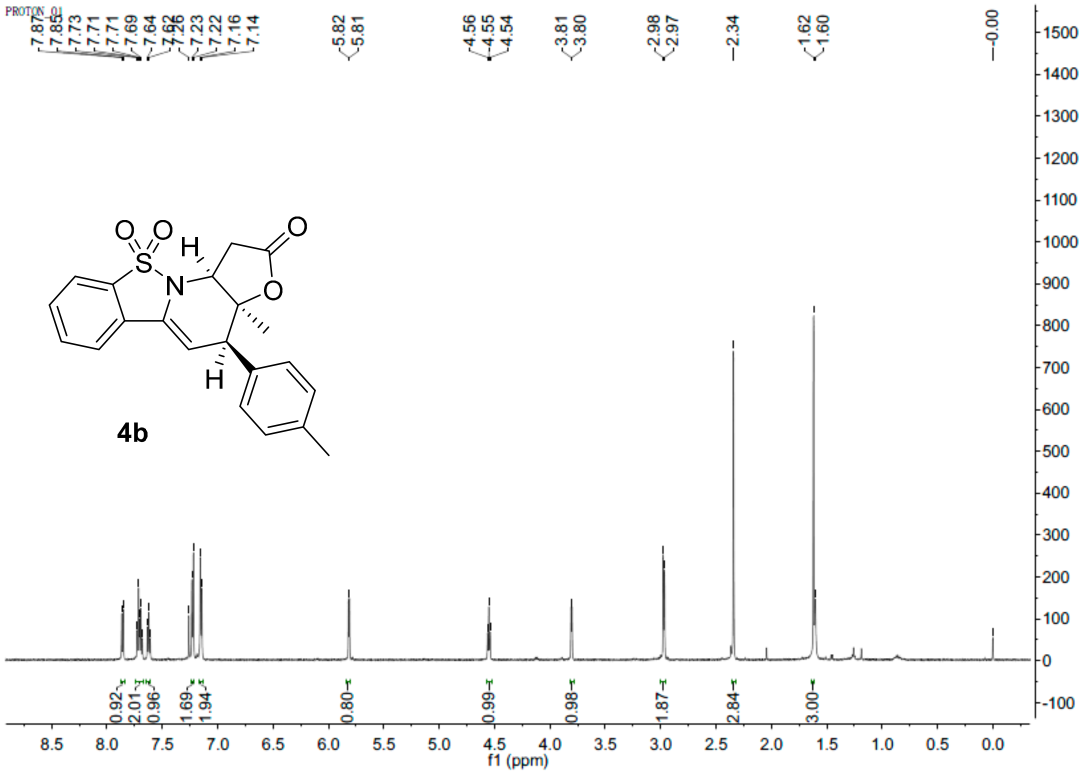
PROTON_01
7.87
7.85
7.73
7.71
7.71
7.69
7.64
7.23
7.22
7.16
7.14
5.82
5.81
4.56
4.55
4.54
3.81
3.80
2.98
2.97
2.34
1.62
1.60
0.00
4b
0.92
2.01
0.96
1.69
1.94
0.80
0.99
0.98
1.87
2.84
3.00
f1 (ppm)

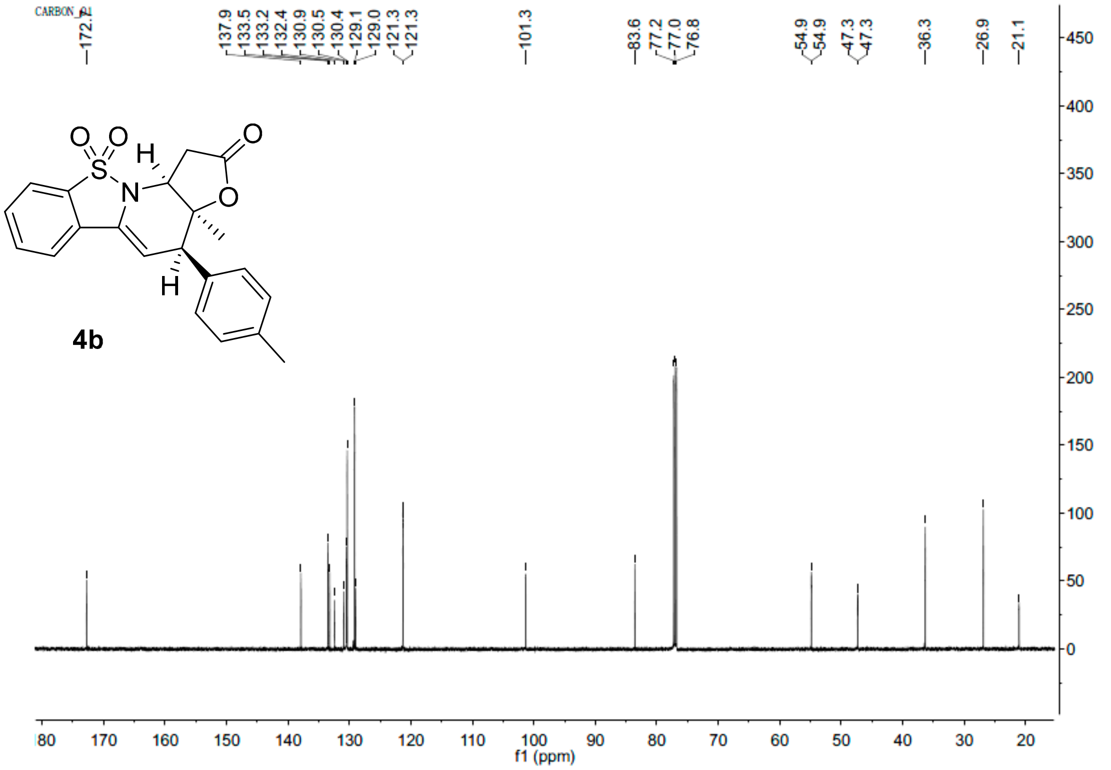
CARBON_01
172.1
137.9
133.5
133.2
132.4
130.9
130.5
130.4
129.1
129.0
121.3
121.3
101.3
83.6
77.2
77.0
76.8
54.9
54.9
47.3
47.3
36.3
26.9
21.1
4b
f1 (ppm)

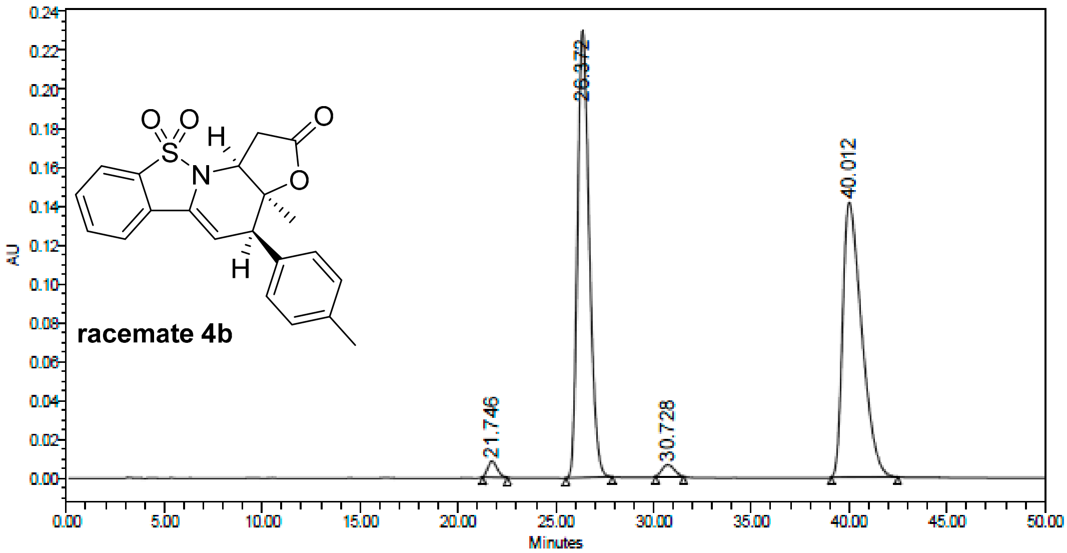

	RT	Area	% Area	Height
1	21.746	276315	1.41	8224
2	26.372	9562954	48.75	229757
3	30.728	258665	1.32	6046
4	40.012	9517600	48.52	140945

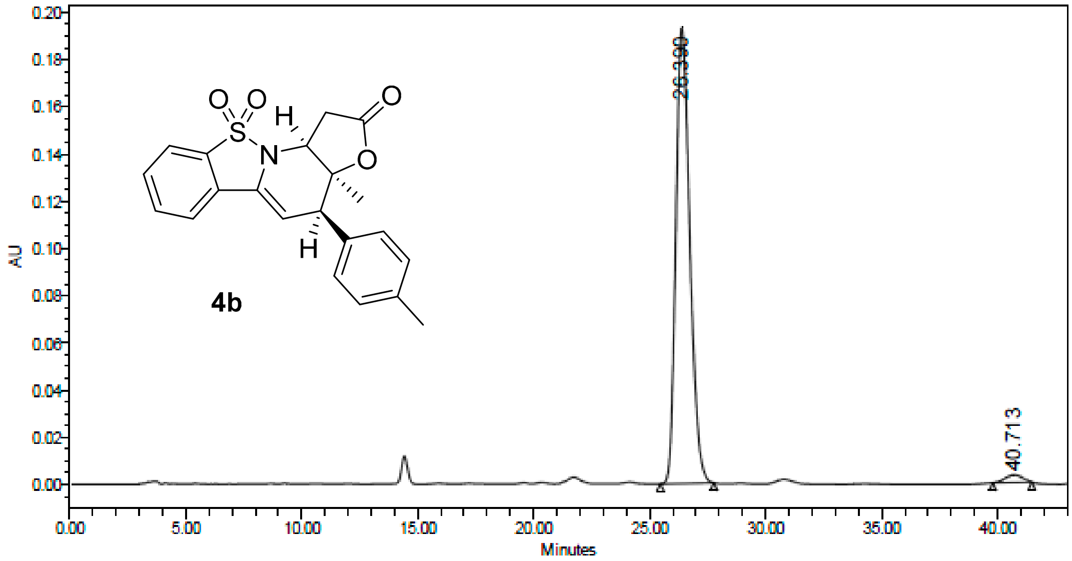

	RT	Area	% Area	Height
1	26.390	8054746	97.99	193261
2	40.713	165058	2.01	3145

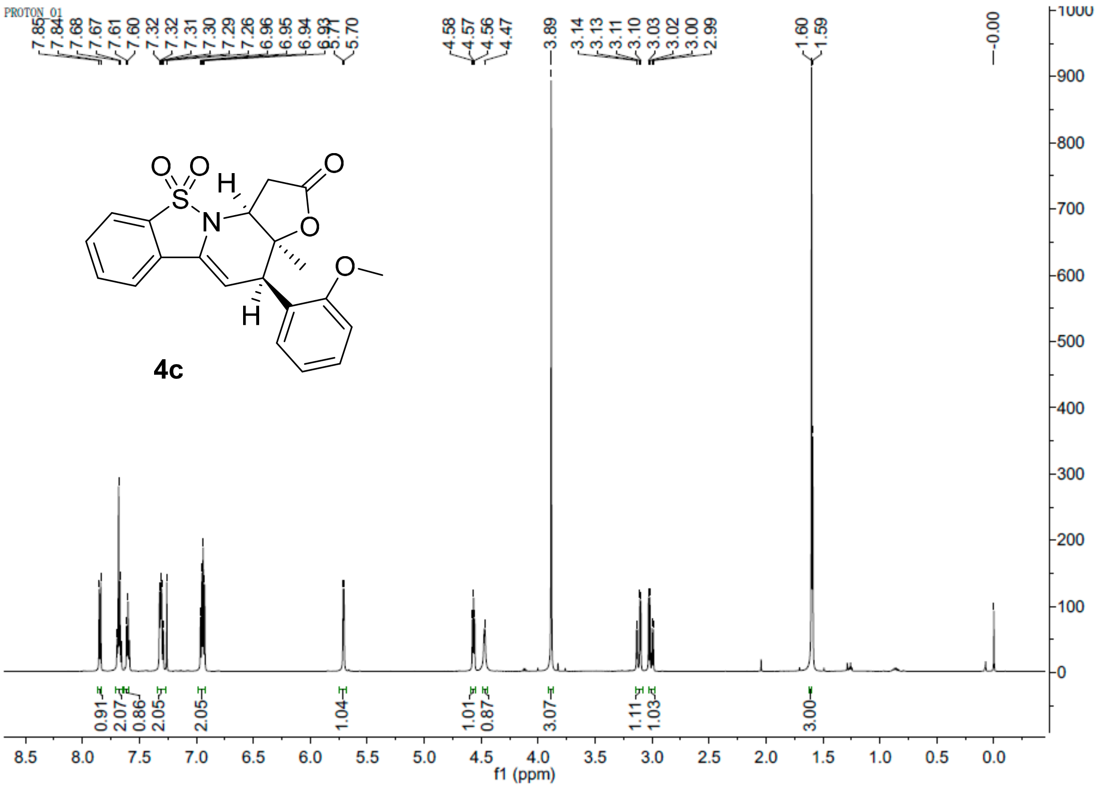
PROTON_01
7.85
7.84
7.68
7.67
7.61
7.60
7.32
7.32
7.31
7.30
7.29
7.26
6.96
6.95
6.94
6.93
5.71
5.70
4.58
4.57
4.56
4.47
3.89
3.14
3.13
3.11
3.10
3.03
3.02
3.00
2.99
1.60
1.59
0.00
4c
0.91
2.07
0.86
2.05
2.05
1.04
1.01
0.87
3.07
1.11
1.03
3.00
f1 (ppm)

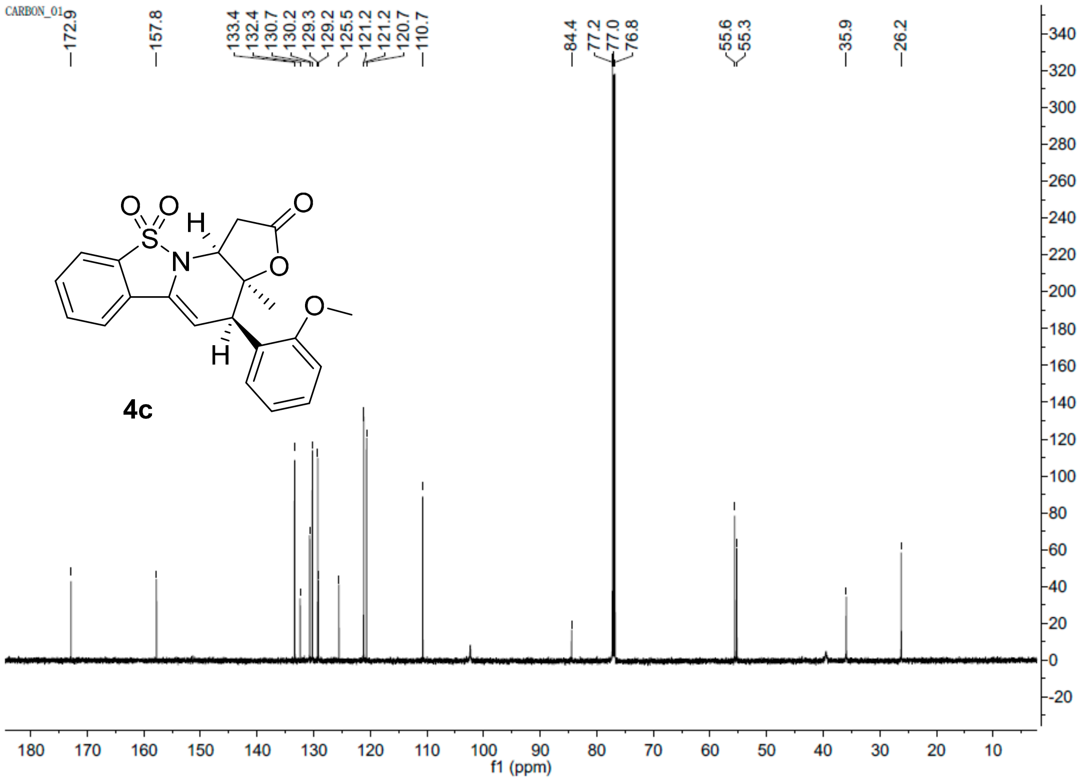
CARBON_01
172.9
157.8
133.4
132.4
130.7
130.2
129.3
129.2
125.5
121.2
121.2
120.7
110.7
84.4
77.2
77.0
76.8
55.6
55.3
35.9
26.2
4c
f1 (ppm)

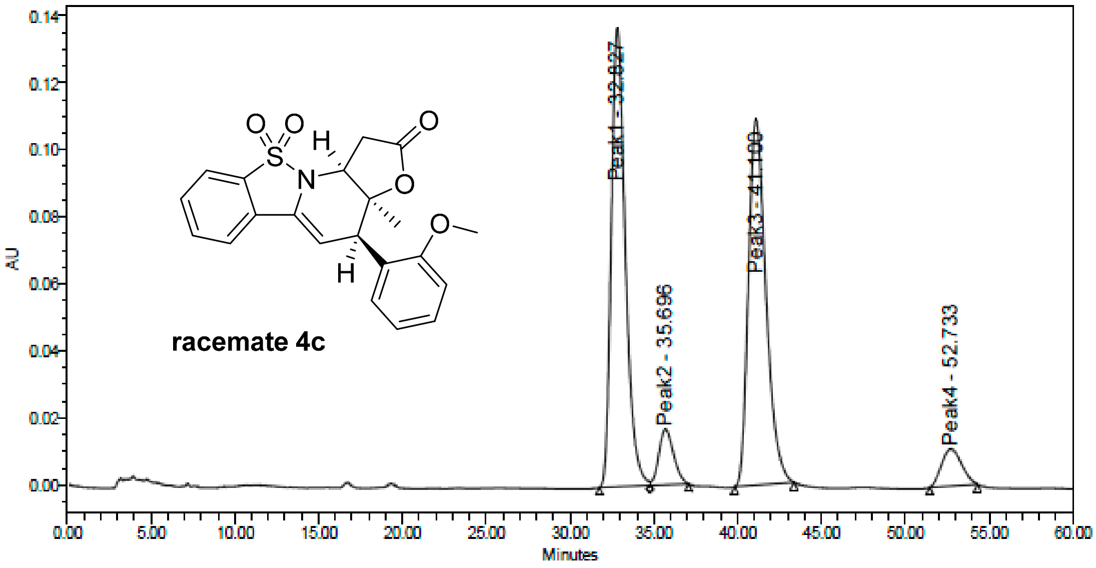

	Peak Name	RT	Area	% Area	Height
1	Peak1	32.827	7971683	44.73	136870
2	Peak2	35.696	1003578	5.63	16577
3	Peak3	41.100	7924881	44.47	109220
4	Peak4	52.733	921693	5.17	11076

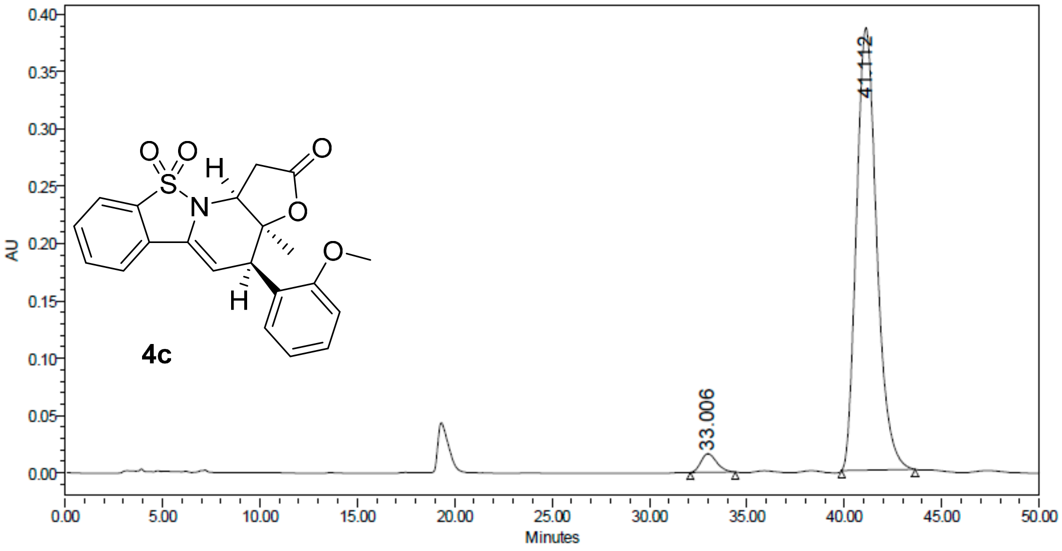

	RT	Area	% Area	Height
1	33.006	912001	3.16	16169
2	41.112	27950441	96.84	385884

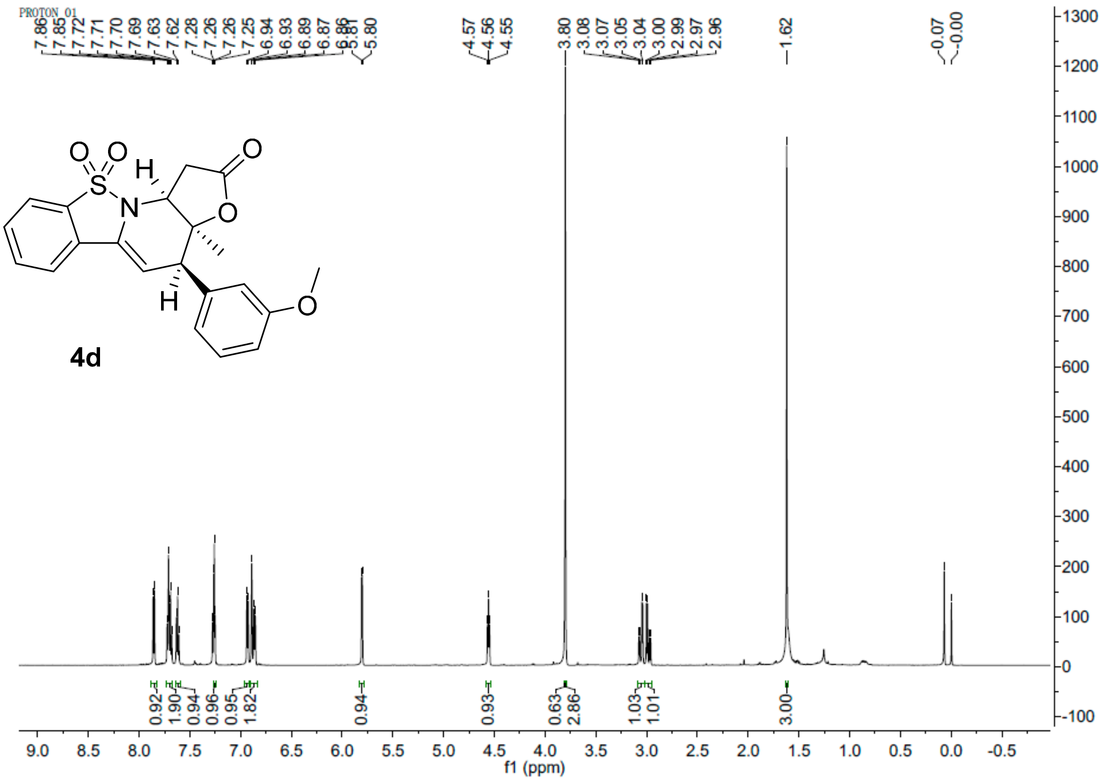
PROTON_01
7.86
7.85
7.72
7.71
7.70
7.69
7.63
7.62
7.28
7.26
7.26
7.25
6.94
6.93
6.89
6.87
6.86
5.81
5.80
4.57
4.56
4.55
3.80
3.08
3.07
3.05
3.04
3.00
2.99
2.97
2.96
1.62
0.07
0.00
4d
0.92
1.90
0.94
0.96
0.95
1.82
0.94
0.93
0.63
2.86
1.03
1.01
3.00
f1 (ppm)

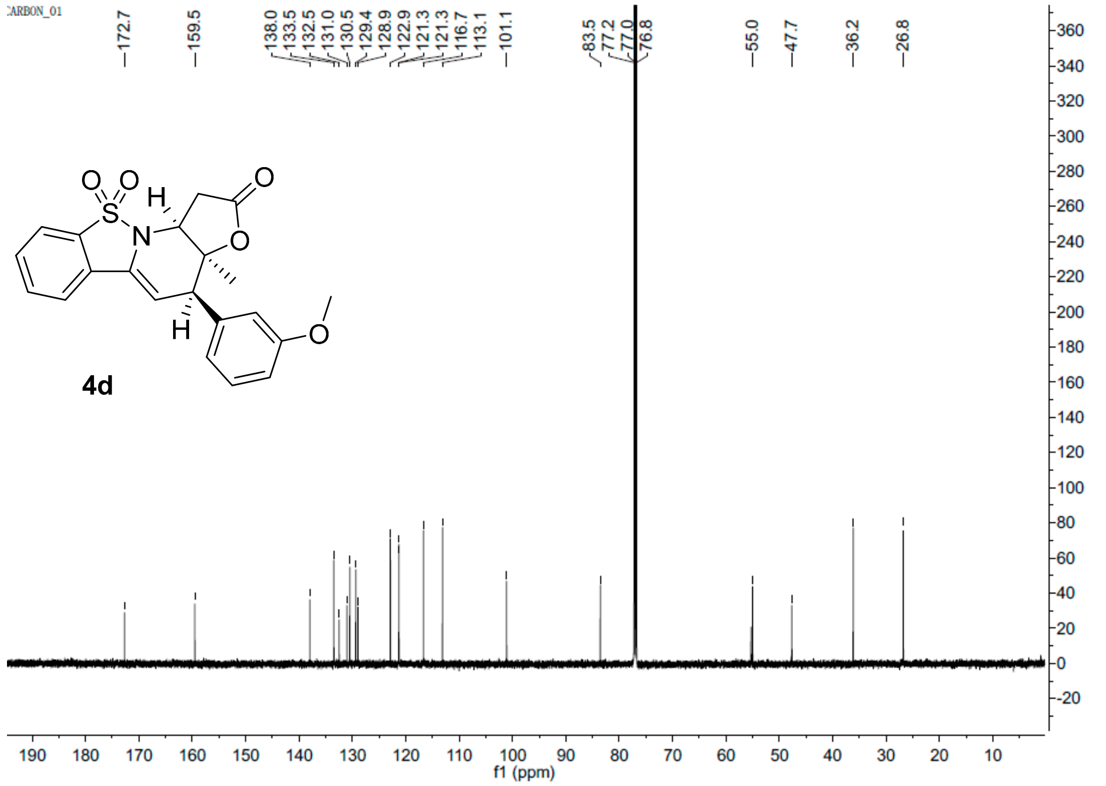
CARBON_01
172.7
159.5
138.0
133.5
132.5
131.0
130.5
129.4
128.9
122.9
121.3
121.3
116.7
113.1
101.1
83.5
77.2
77.0
76.8
55.0
47.7
36.2
26.8
4d
f1 (ppm)

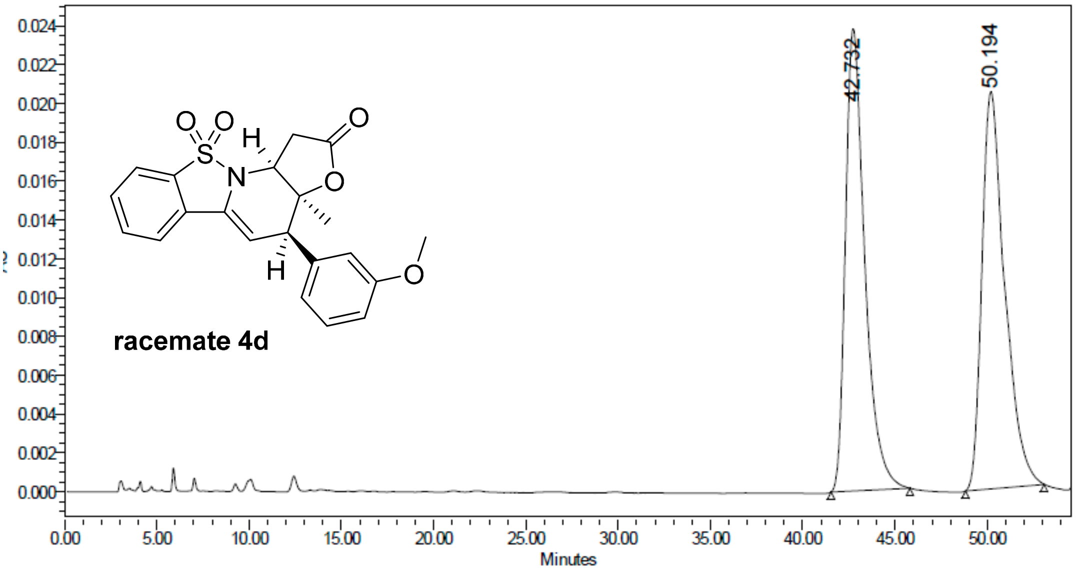

	RT	Area	% Area	Height
1	42.732	1734197	49.58	23804
2	50.194	1763332	50.42	20459

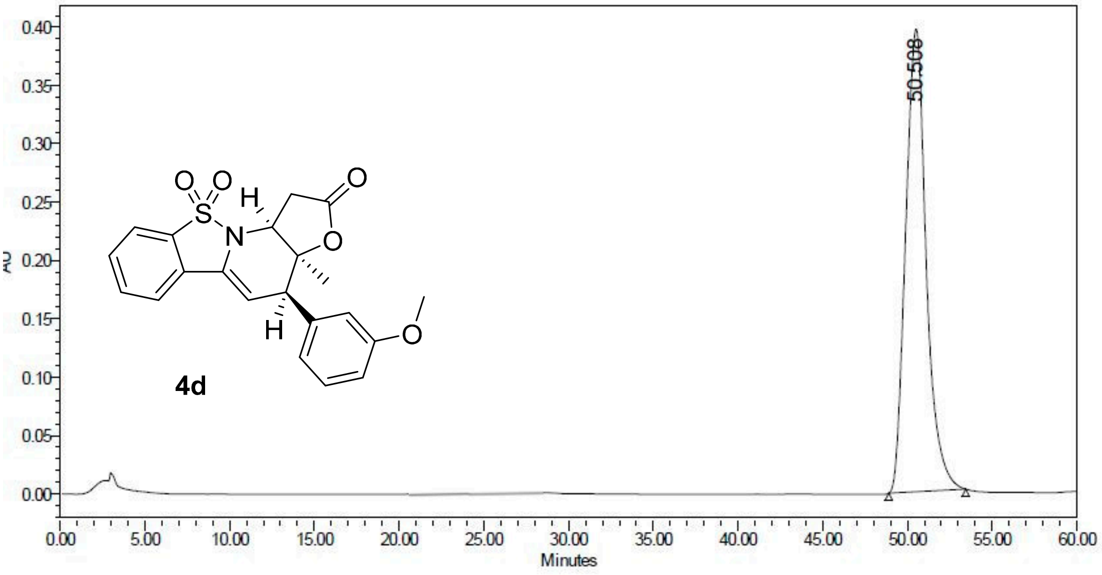

	RT	Area	% Area	Height
1	50.508	33483180	100.00	396374

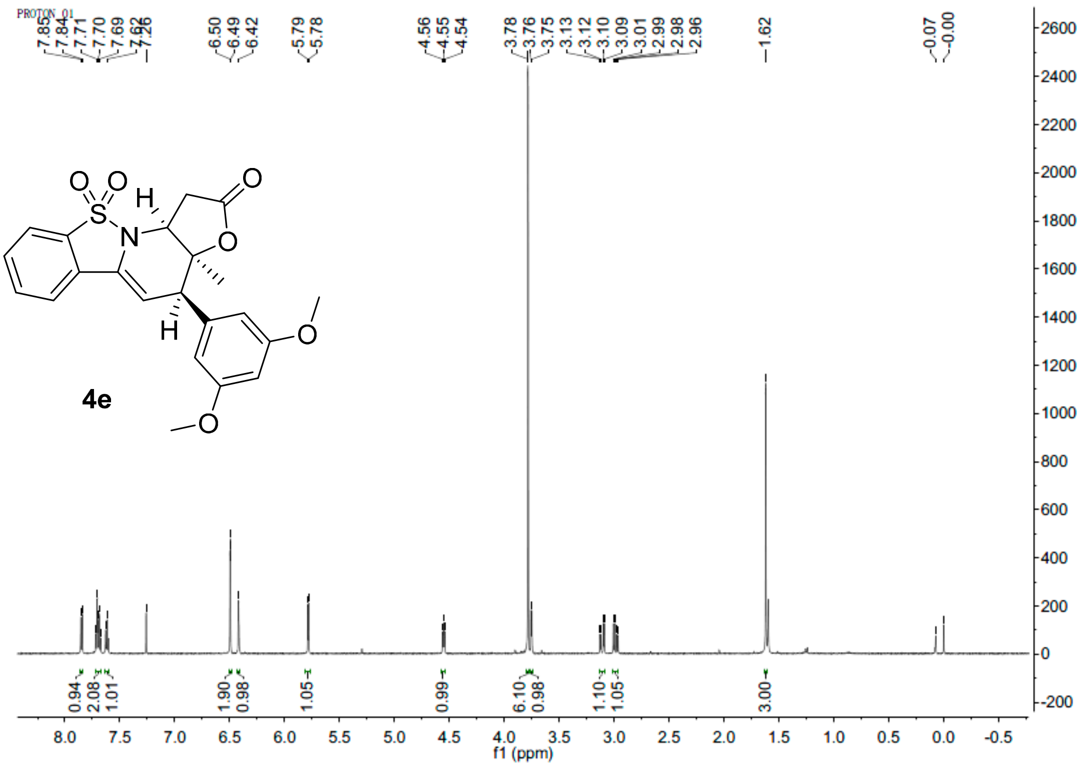

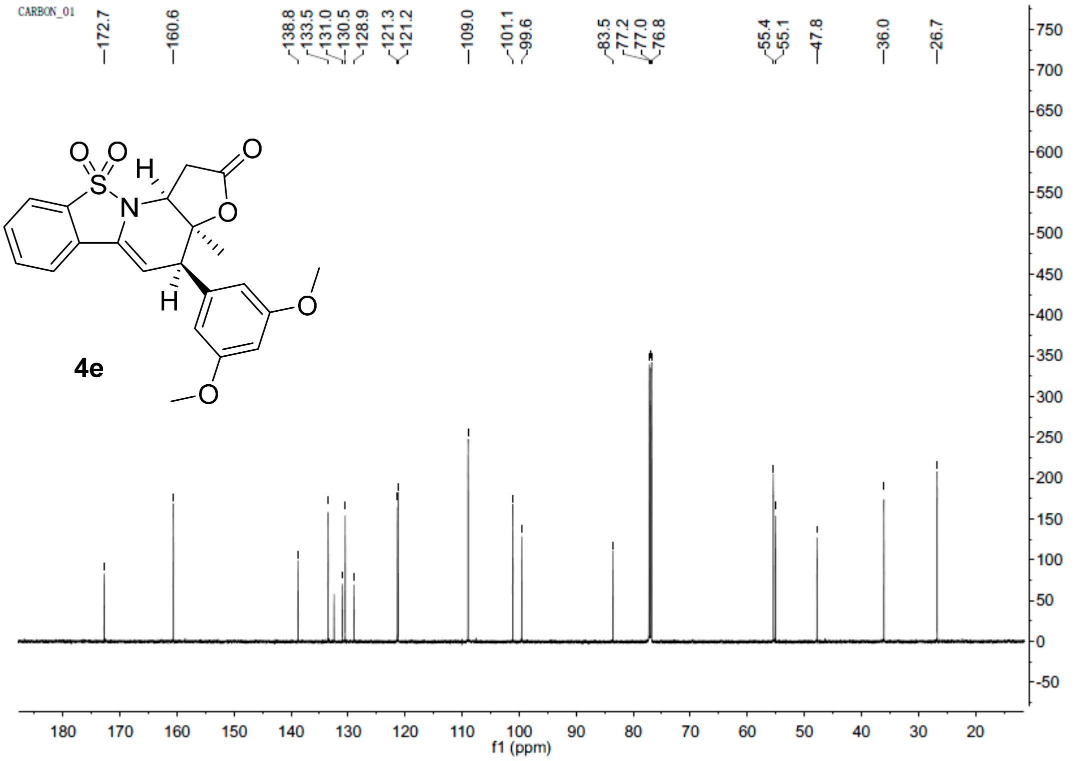

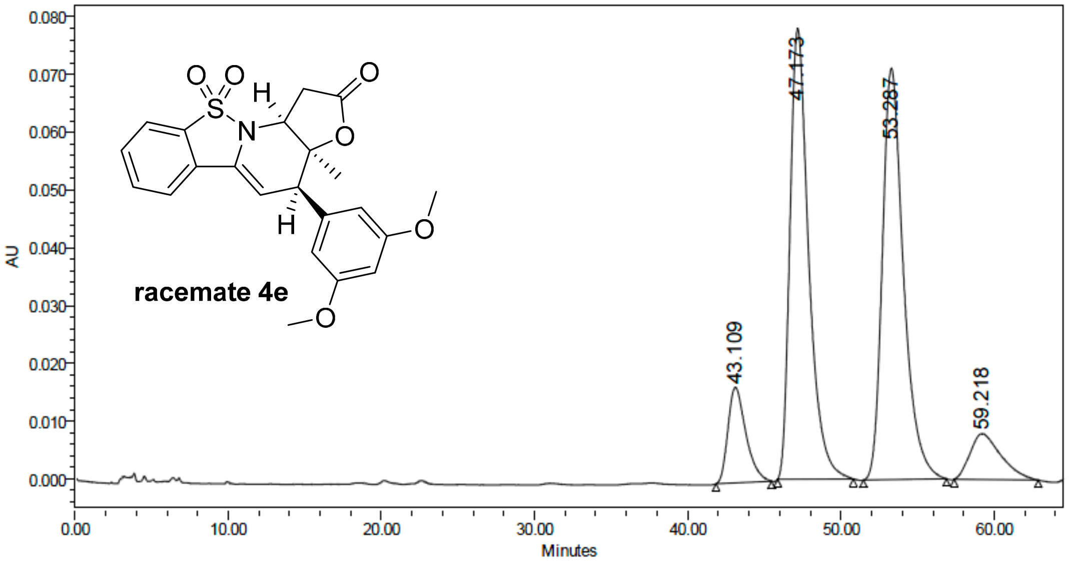

	RT	Area	% Area	Height
1	43.109	1346765	8.47	16496
2	47.173	6611212	41.58	78021
3	53.287	6823393	42.92	71199
4	59.218	1118237	7.03	7953

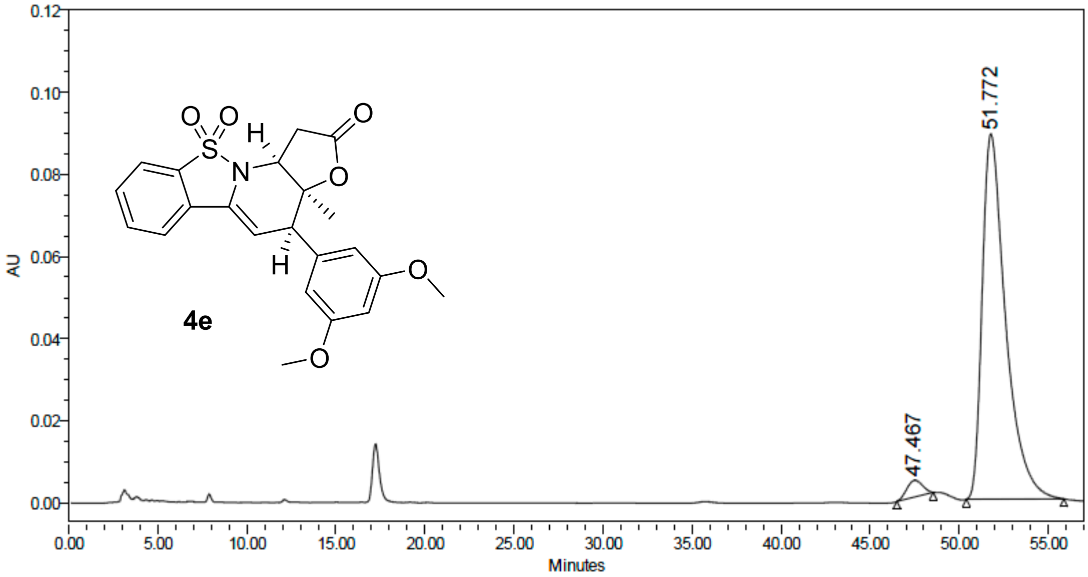

	RT	Area	% Area	Height
1	47.467	246945	2.95	4059
2	51.772	8132592	97.05	88859

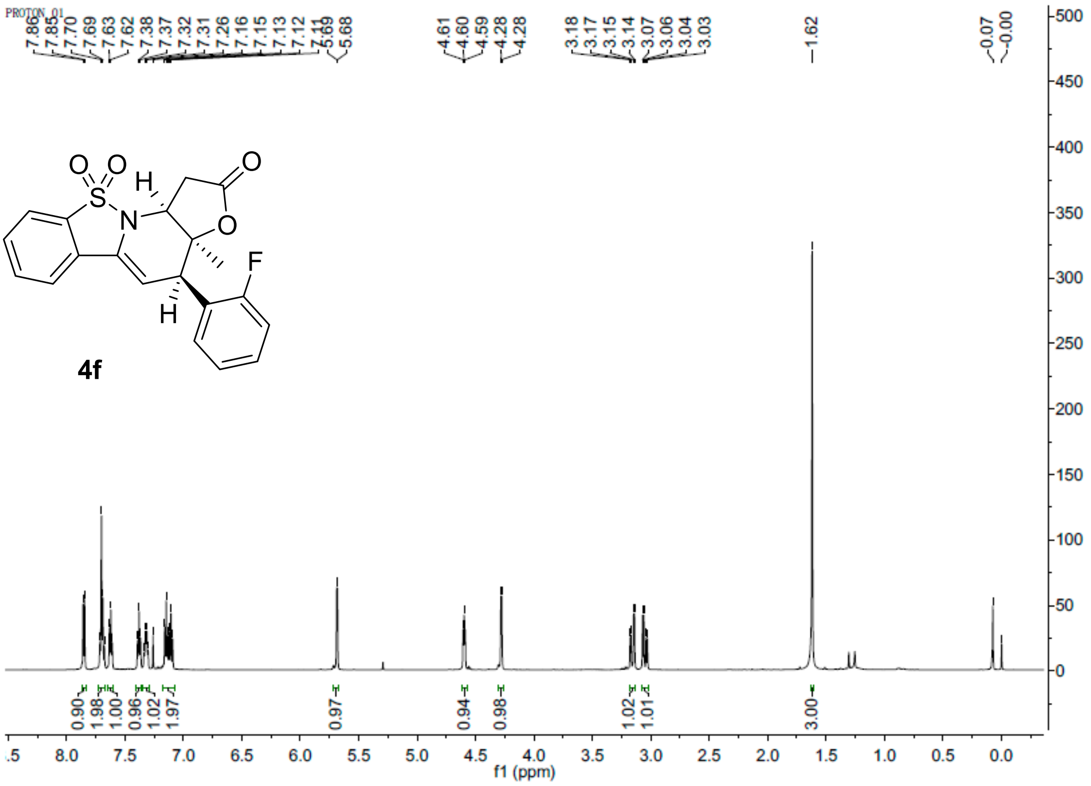

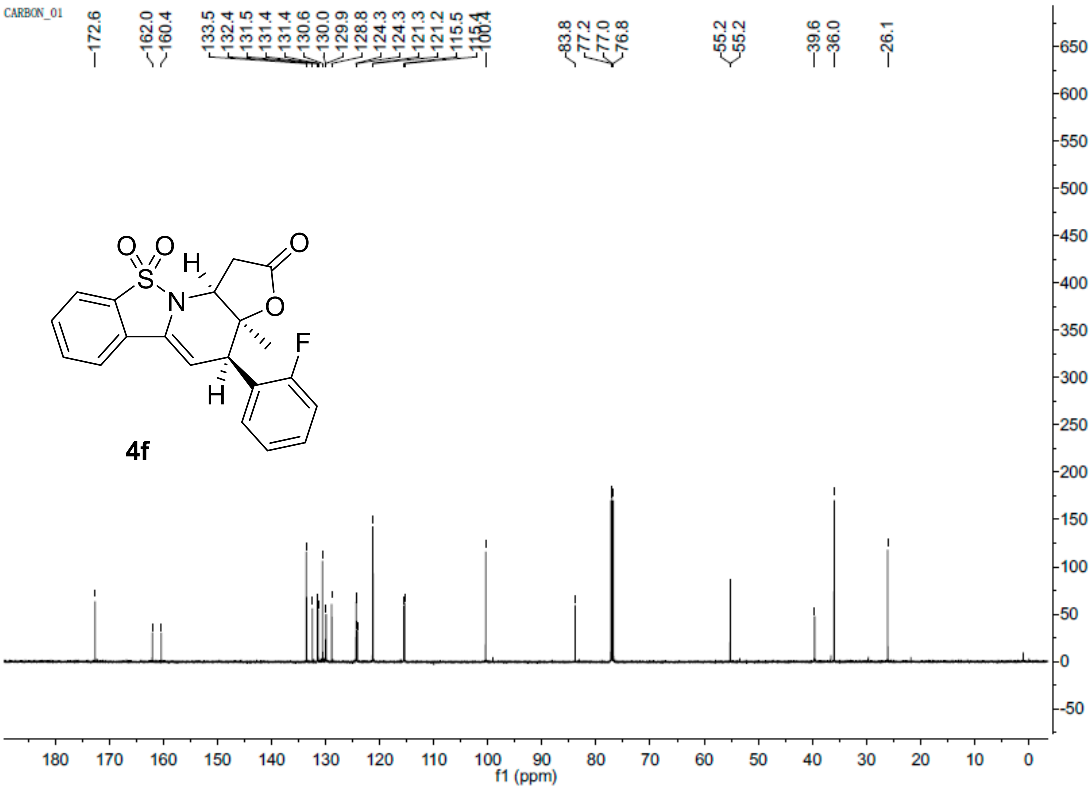

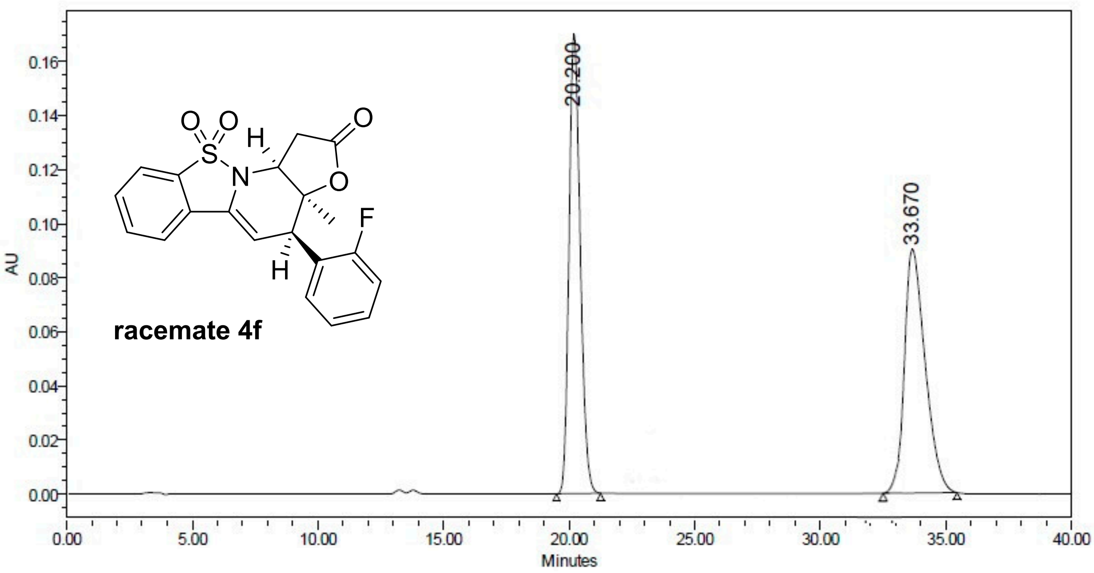

	RT	Area	% Area	Height
1	20.200	5250167	50.43	170013
2	33.670	5160455	49.57	90352

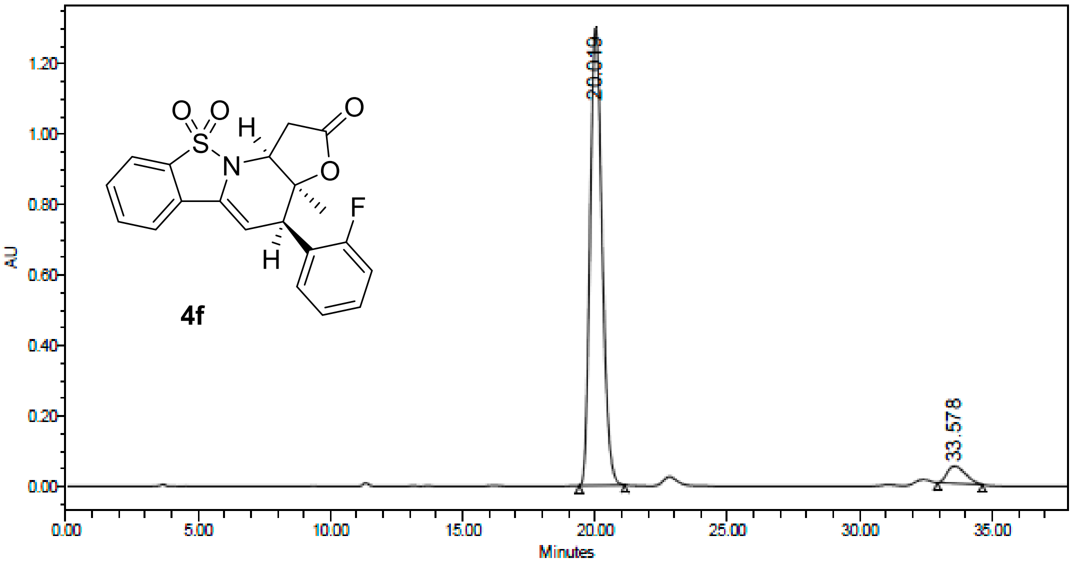

	RT	Area	% Area	Height
1	20.019	40831708	94.51	1299700
2	33.578	2371705	5.49	49007

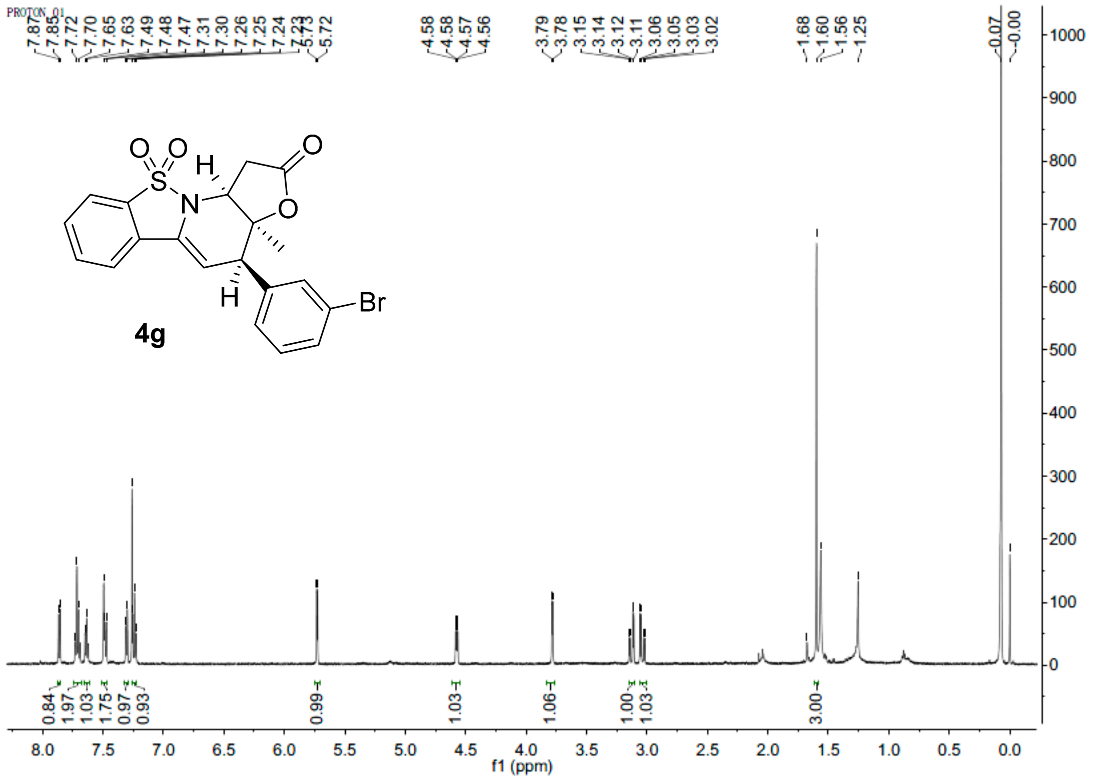
PROTON_01
7.87 7.85 7.72 7.70 7.65 7.63 7.49 7.48 7.47 7.31 7.30 7.26 7.25 7.24 7.23 5.73 5.72
4.58 4.58 4.57 4.56
3.79 3.78 3.15 3.14 3.12 3.11 3.06 3.05 3.03 3.02
1.68 1.60 1.56 1.25
0.07 -0.00
0.84 1.97 1.03 1.75 0.97 0.93
0.99
1.03
1.06
1.00 1.03
3.00
4g
Br
f1 (ppm)

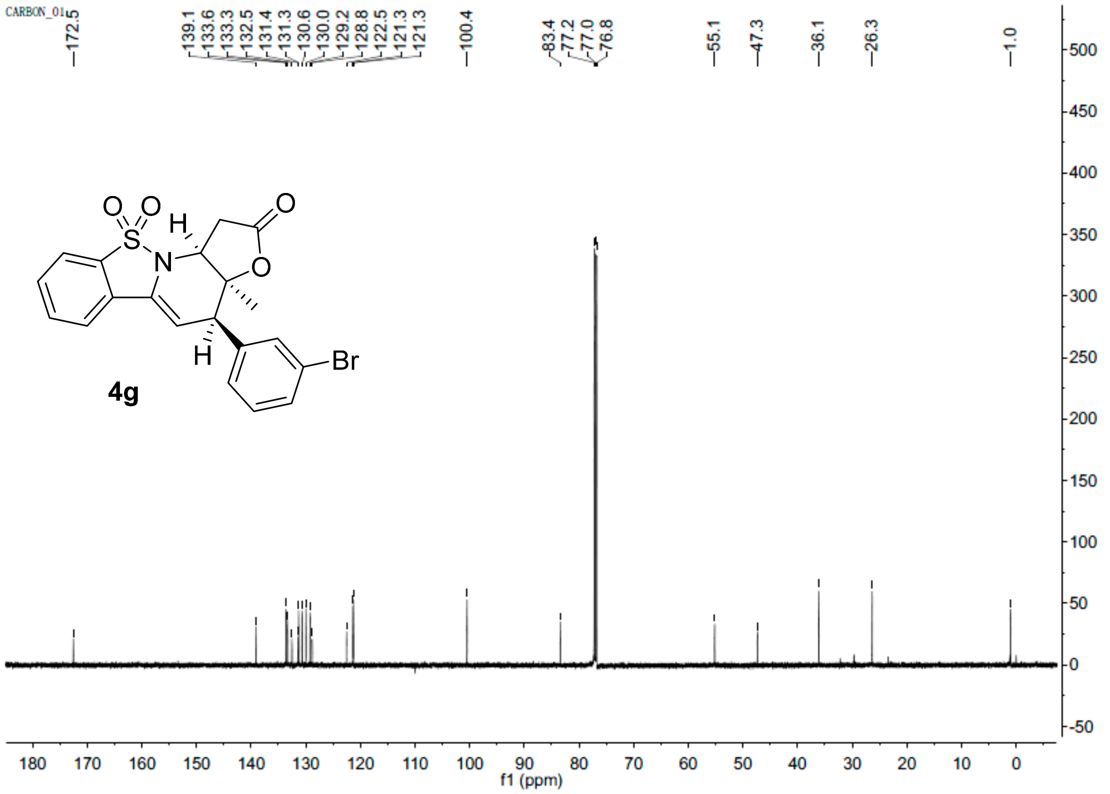
CARBON_01
172.5
139.1 133.6 133.3 132.5 131.4 131.3 130.6 130.0 129.2 128.8 122.5 121.3 121.3
100.4
83.4 77.2 77.0 76.8
55.1
47.3
36.1
26.3
1.0
4g
Br
f1 (ppm)

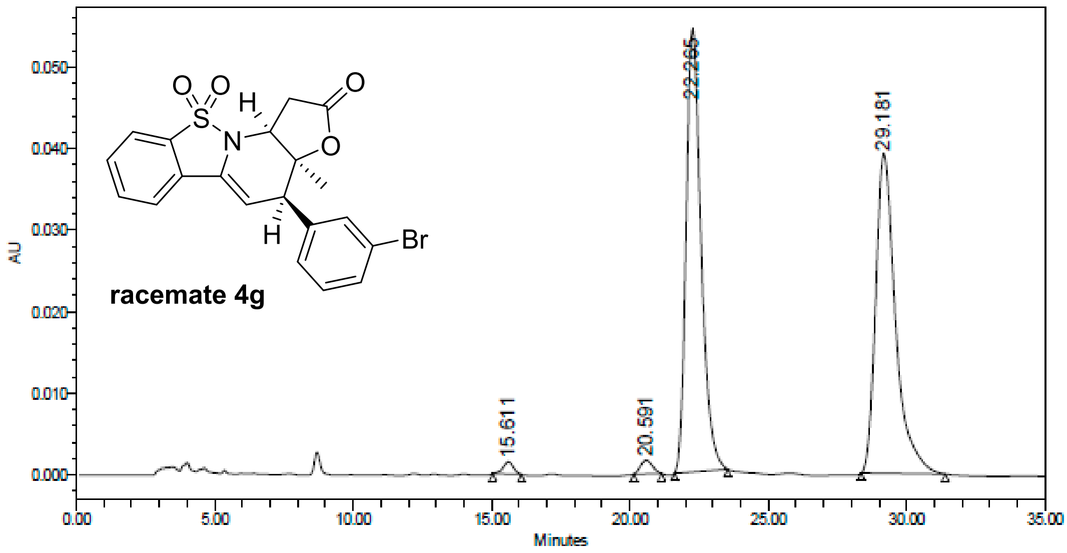

	RT	Area	% Area	Height
1	15.611	38976	0.95	1475
2	20.591	48658	1.19	1636
3	22.265	2040259	49.93	54265
4	29.181	1958268	47.92	39116

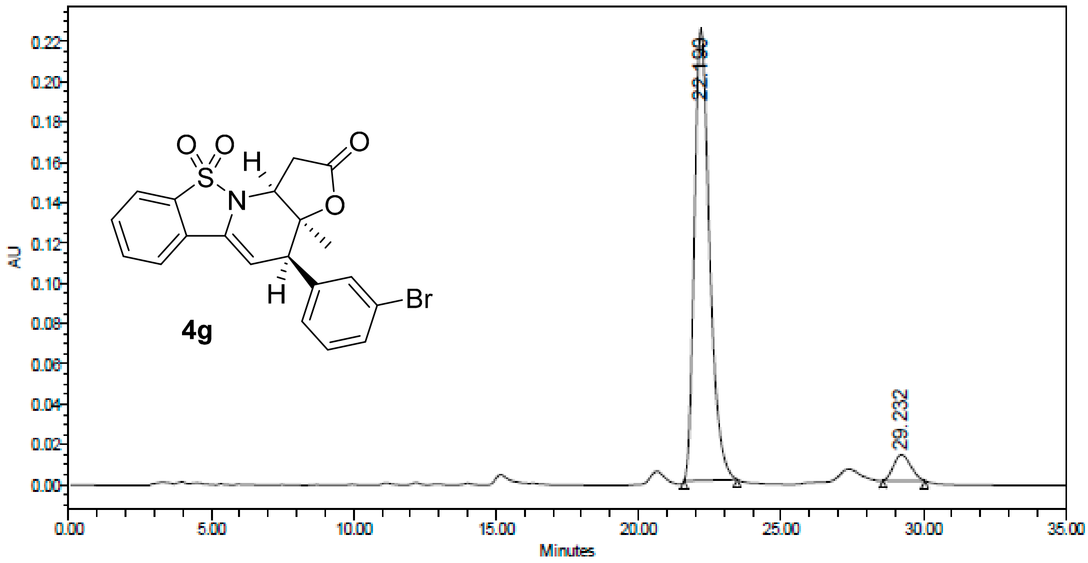

	RT	Area	% Area	Height
1	22.190	8284859	93.87	224216
2	29.232	540744	6.13	12918

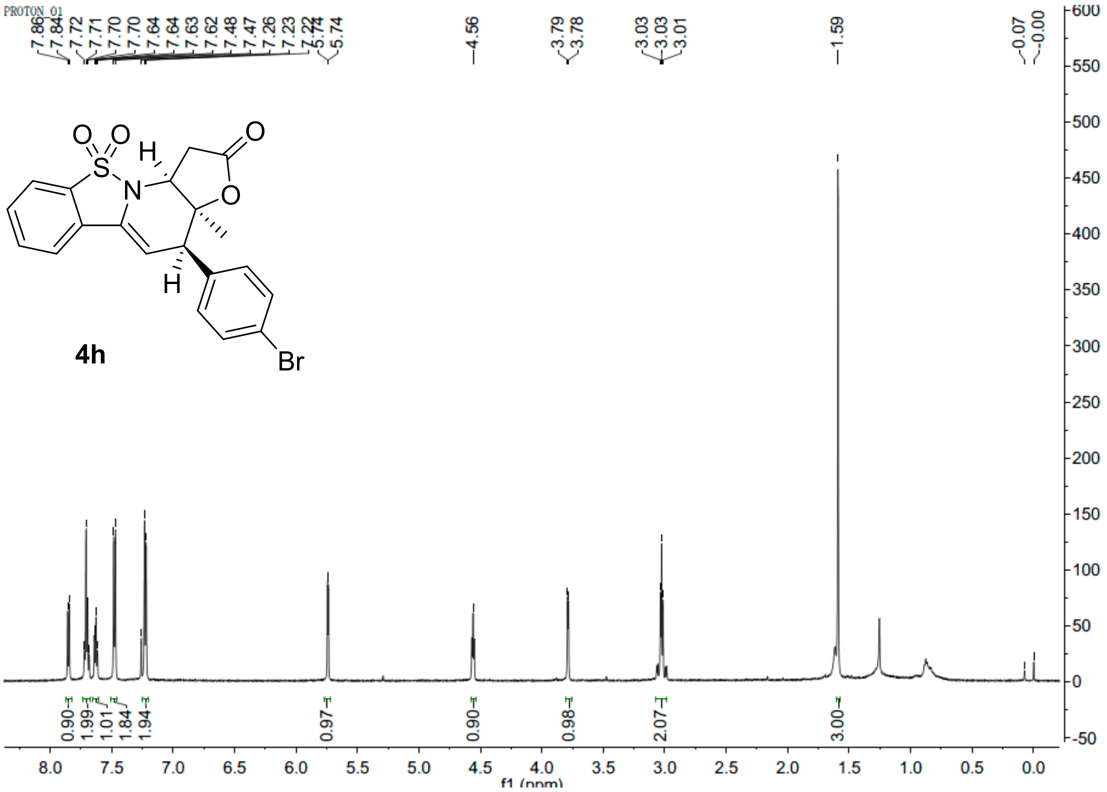
PROTON_01
4h
f1 (ppm)

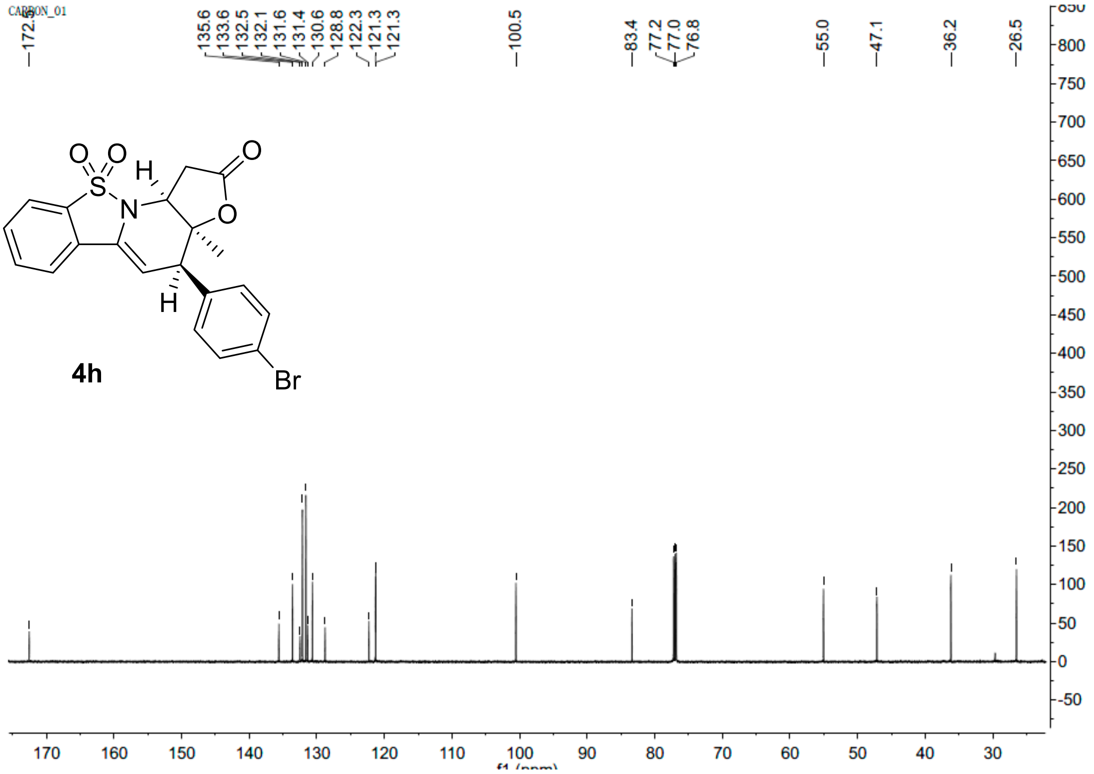
CARBON_01
4h
f1 (ppm)

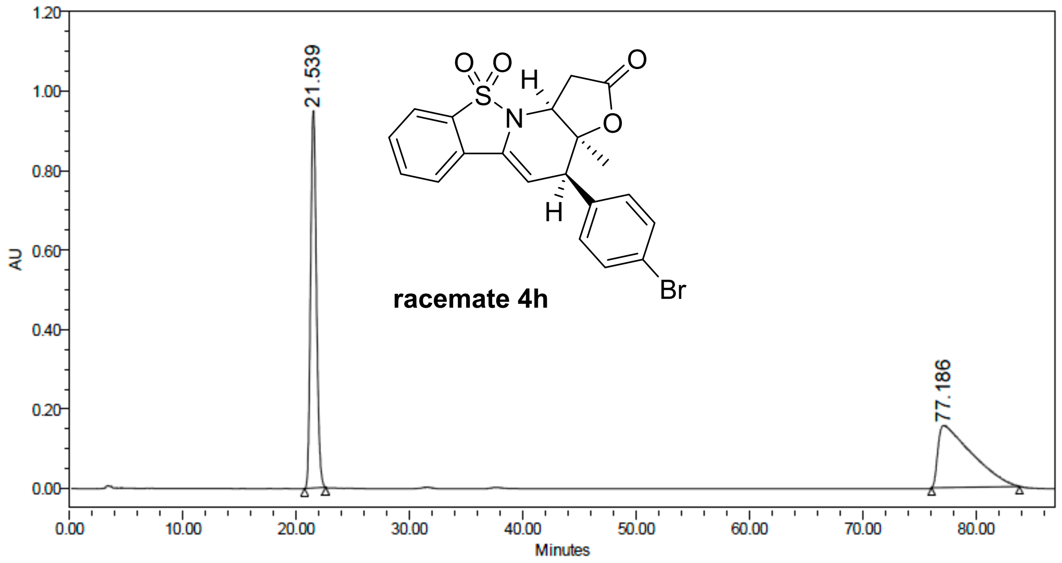

	RT	Area	% Area	Height
1	21.539	33943648	51.20	949857
2	77.186	32347438	48.80	156180

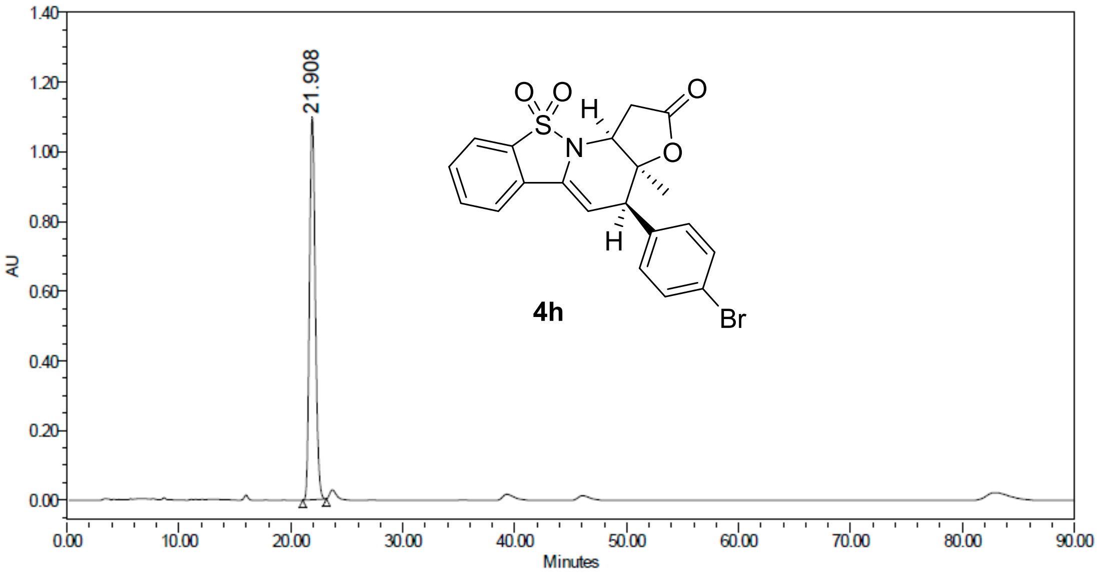

	RT	Area	% Area	Height
1	21.908	39559832	100.00	1098527

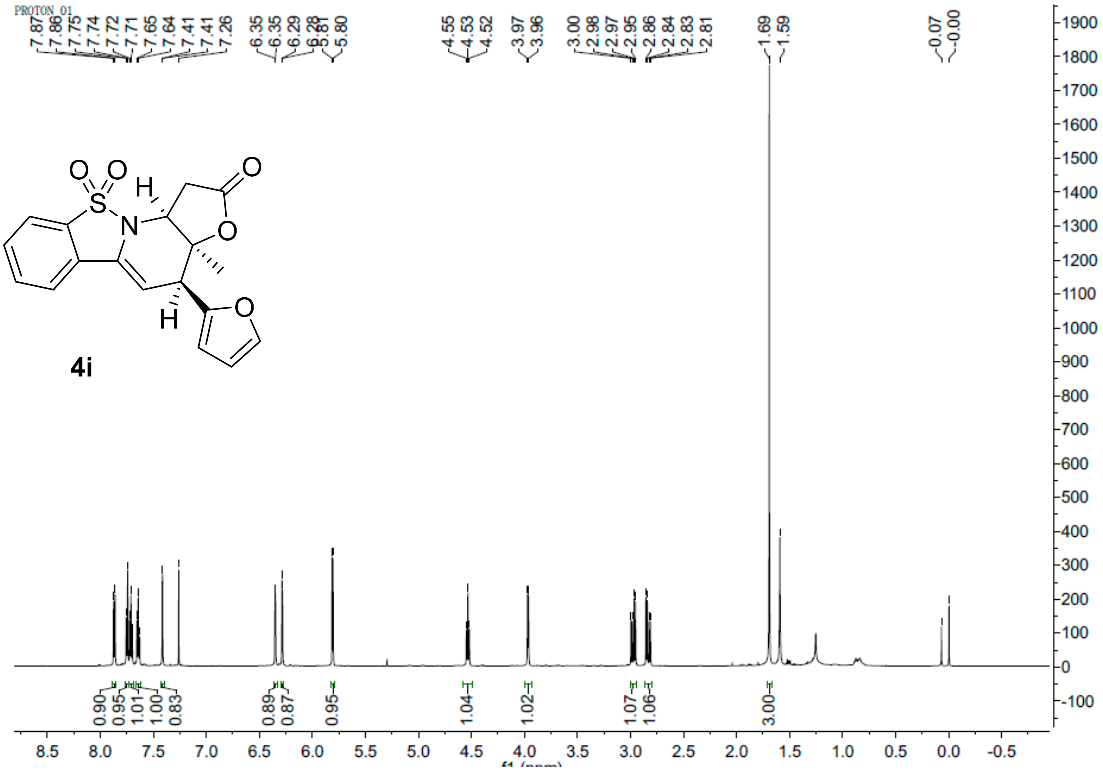
PROTON_01
7.87 7.86 7.75 7.74 7.72 7.71 7.65 7.64 7.41 7.41 7.26
6.35 6.35 6.29 6.28 5.81 5.80
4.55 4.53 4.52
3.97 3.96
3.00 2.98 2.97 2.95 2.86 2.84 2.83 2.81
1.69 1.59
0.07 -0.00
4i
0.90 0.95 1.01 1.00 0.83 0.89 0.87 0.95 1.04 1.02 1.07 1.06 3.00
f1 (ppm)

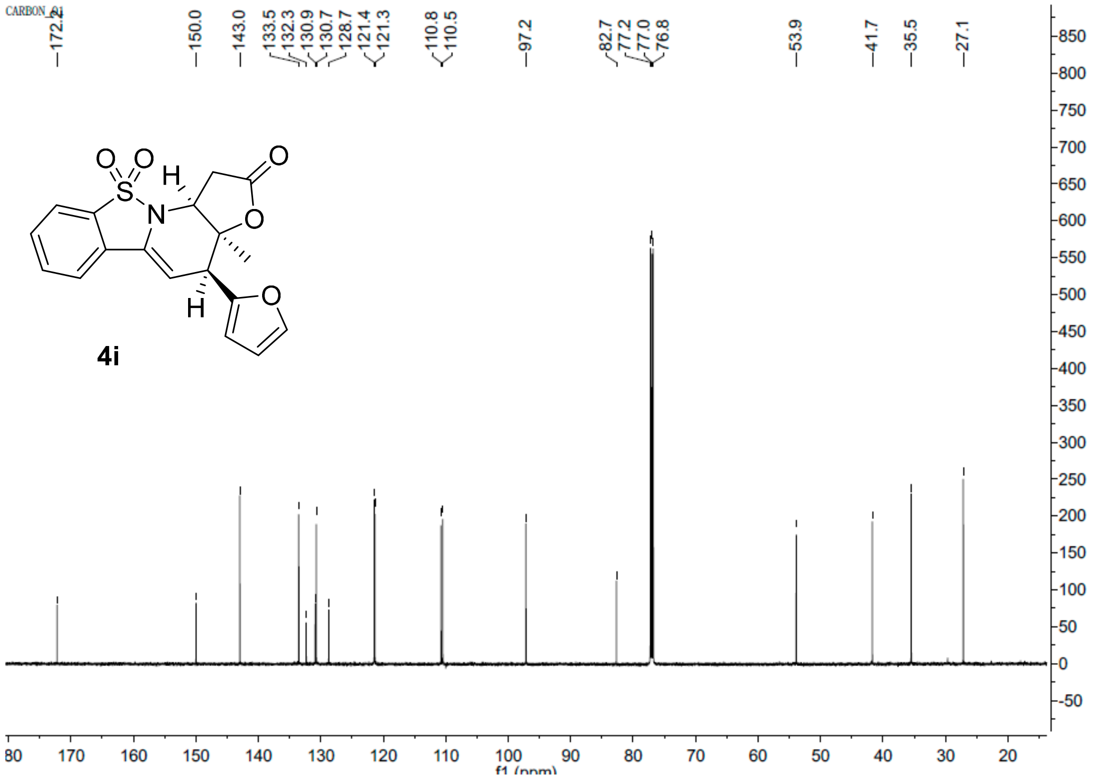
CARBON_01
172.2
150.0
143.0
133.5 132.3 130.9 130.7 128.7 121.4 121.3
110.8 110.5
97.2
82.7 77.2 77.0 76.8
53.9
41.7
35.5
27.1
4i
f1 (ppm)

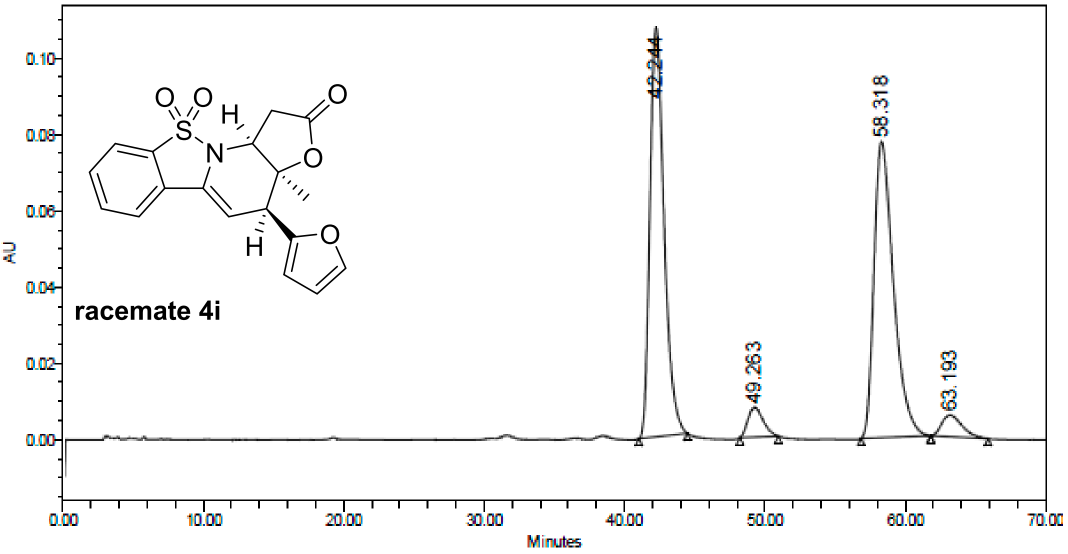

	RT	Area	% Area	Height
1	42.244	7482839	46.43	107305
2	49.263	584841	3.63	7774
3	58.318	7482430	46.43	77582
4	63.193	566023	3.51	5687

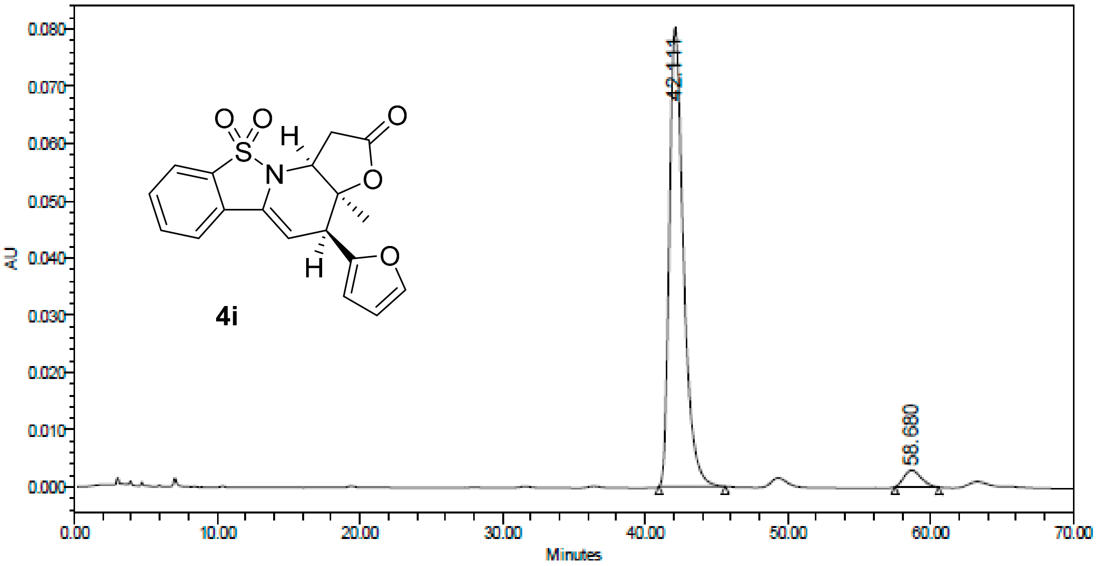

	RT	Area	% Area	Height
1	42.111	5506236	95.74	80189
2	58.680	245288	4.26	2895

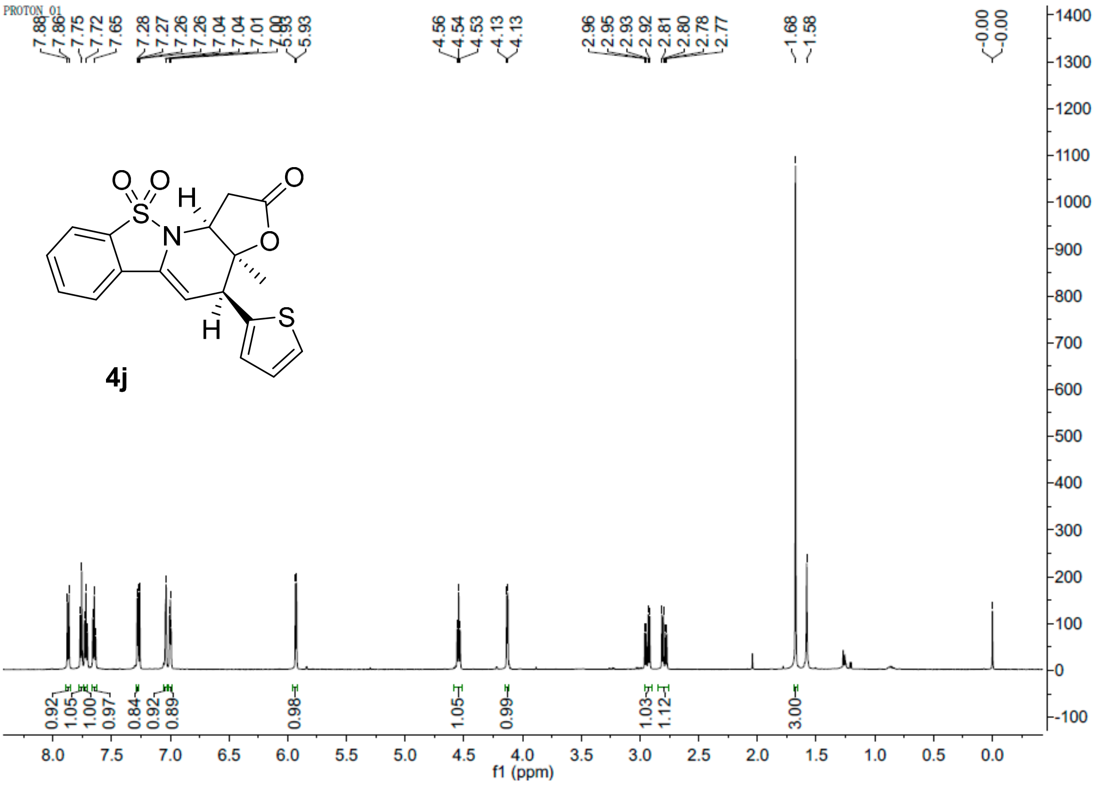
PROTON_01
7.88
7.86
7.75
7.72
7.65
7.28
7.27
7.26
7.26
7.04
7.04
7.01
7.00
5.93
5.93
4.56
4.54
4.53
4.13
4.13
2.96
2.95
2.93
2.92
2.81
2.80
2.78
2.77
1.68
1.58
0.00
0.00
4j
0.92
1.05
1.00
0.97
0.84
0.92
0.89
0.98
1.05
0.99
1.03
1.12
3.00
f1 (ppm)

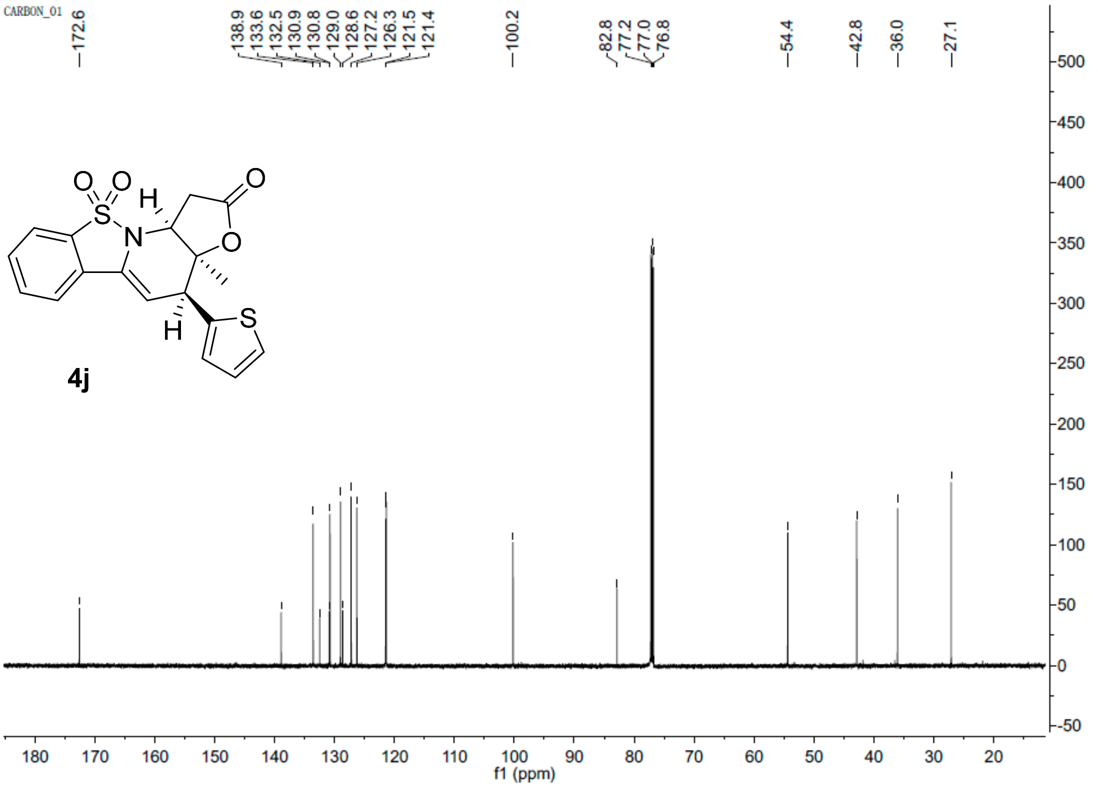
CARBON_01
172.6
138.9
133.6
132.5
130.9
130.8
129.0
128.6
127.2
126.3
121.5
121.4
100.2
82.8
77.2
77.0
76.8
54.4
42.8
36.0
27.1
4j
f1 (ppm)

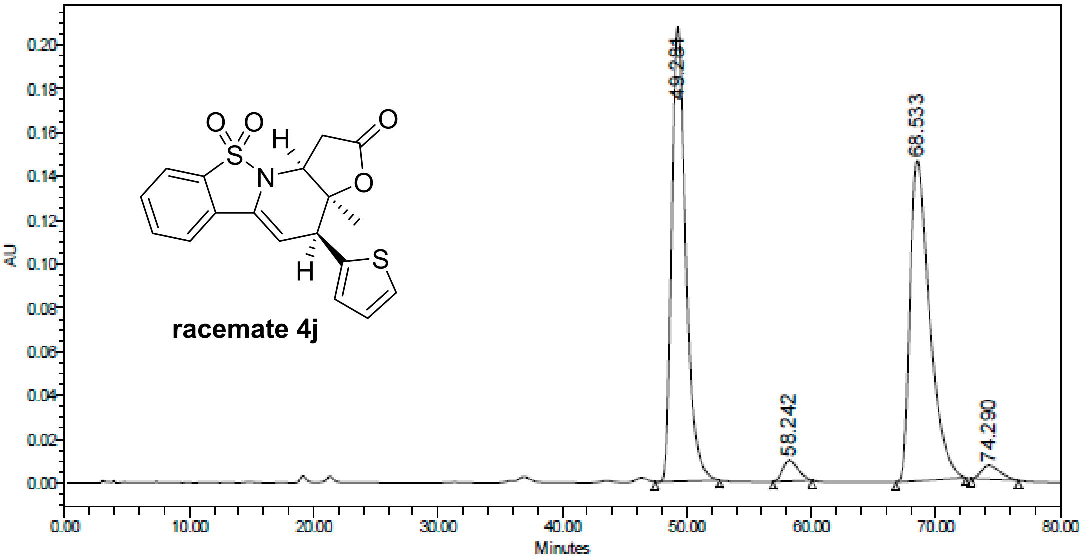

	RT	Area	% Area	Height
1	49.281	16380653	48.23	207160
2	58.242	808300	2.38	9421
3	68.533	16107054	47.43	146183
4	74.290	666806	1.96	6305

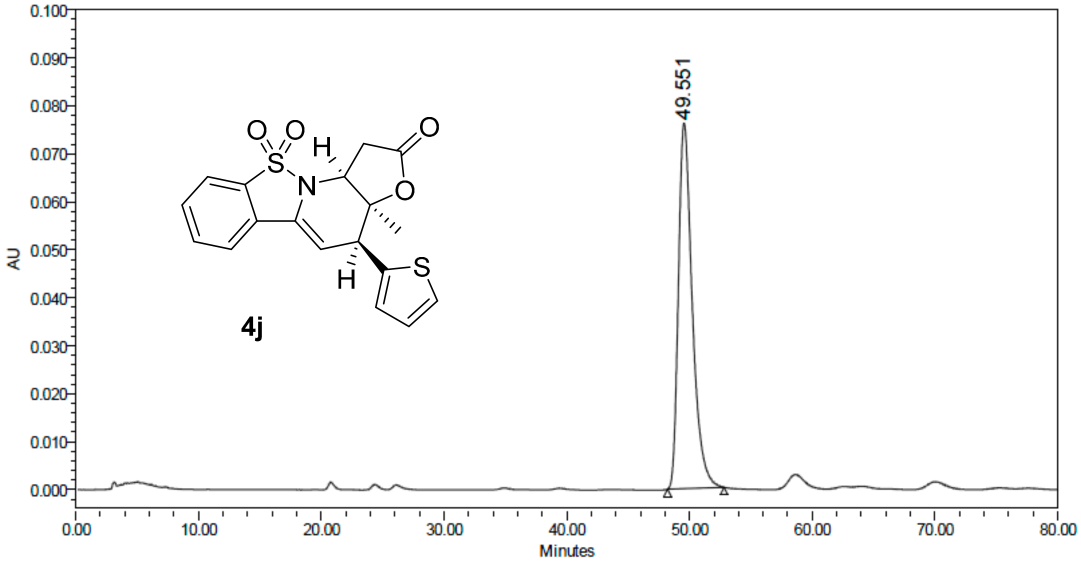

	RT	Area	% Area	Height
1	49.551	5961579	100.00	76135

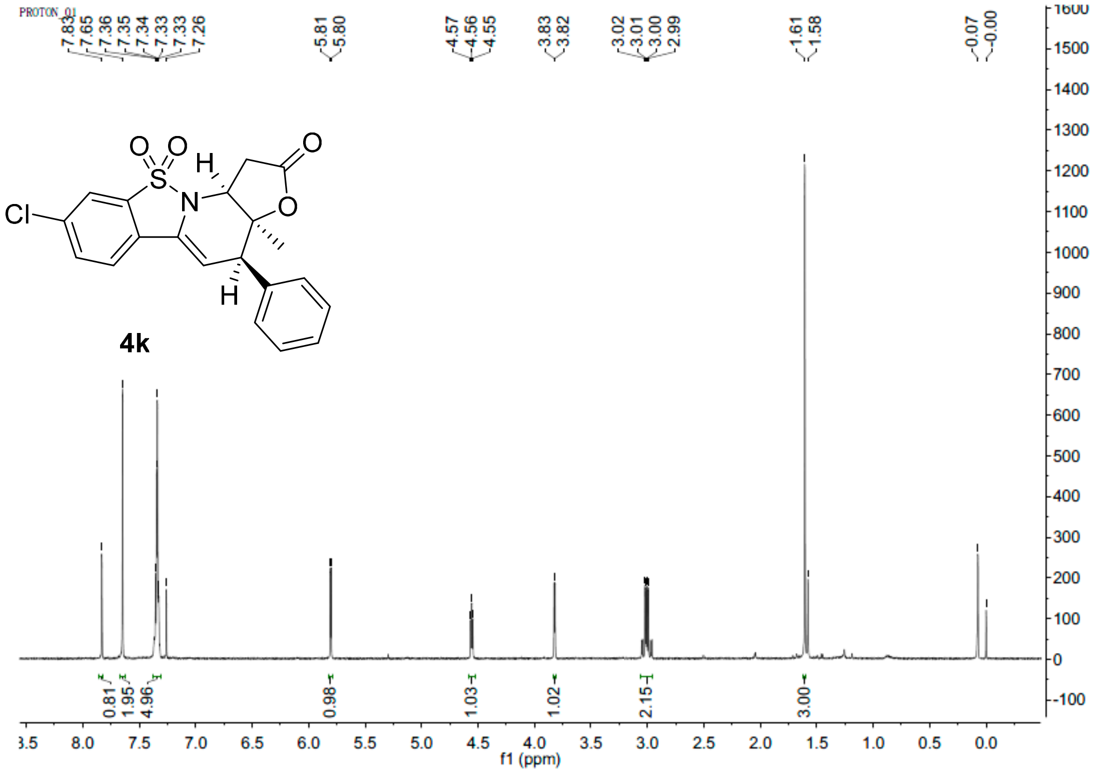
PROTON_01
7.83
7.65
7.36
7.35
7.34
7.33
7.33
7.26
5.81
5.80
4.57
4.56
4.55
3.83
3.82
3.02
3.01
3.00
2.99
1.61
1.58
0.07
0.00
Cl
O
S
N
H
4k
0.81
1.95
4.96
0.98
1.03
1.02
2.15
3.00
f1 (ppm)

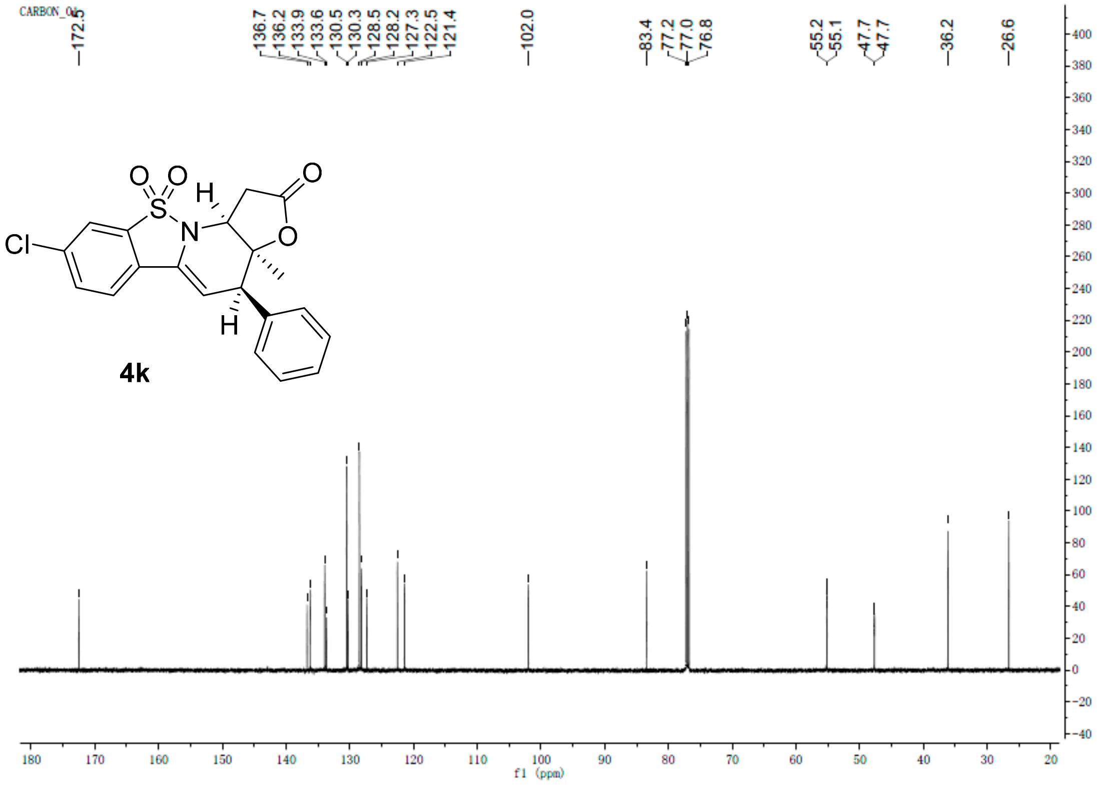
CARBON_01
172.5
136.7
136.2
133.9
133.6
130.5
130.3
128.5
128.2
127.3
122.5
121.4
102.0
83.4
77.2
77.0
76.8
55.2
55.1
47.7
47.7
36.2
26.6
Cl
O
S
N
H
4k
f1 (ppm)

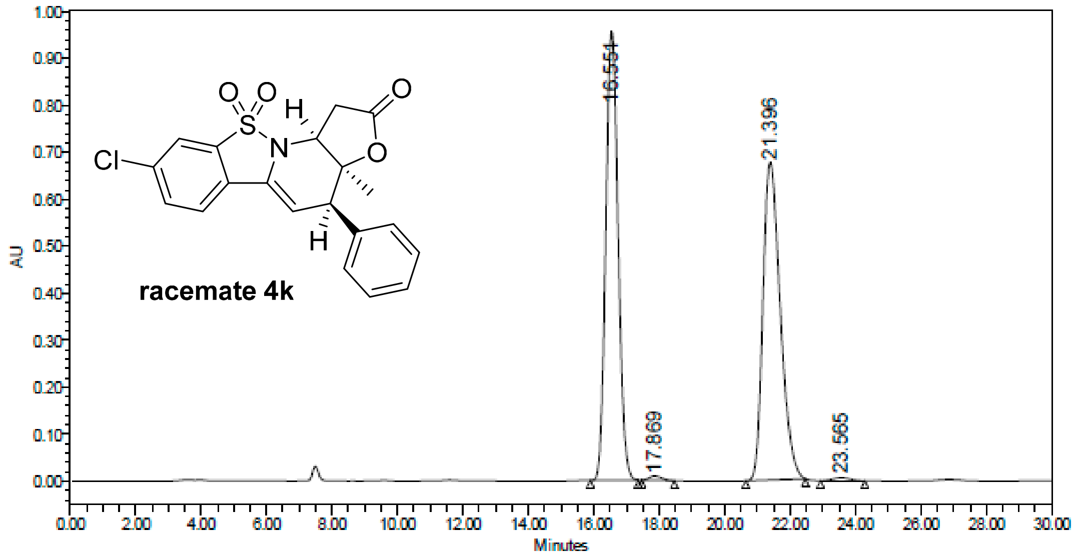

	RT	Area	% Area	Height
1	16.551	24740656	49.45	954355
2	17.869	255013	0.51	9600
3	21.396	24802676	49.57	675931
4	23.565	232287	0.46	6382

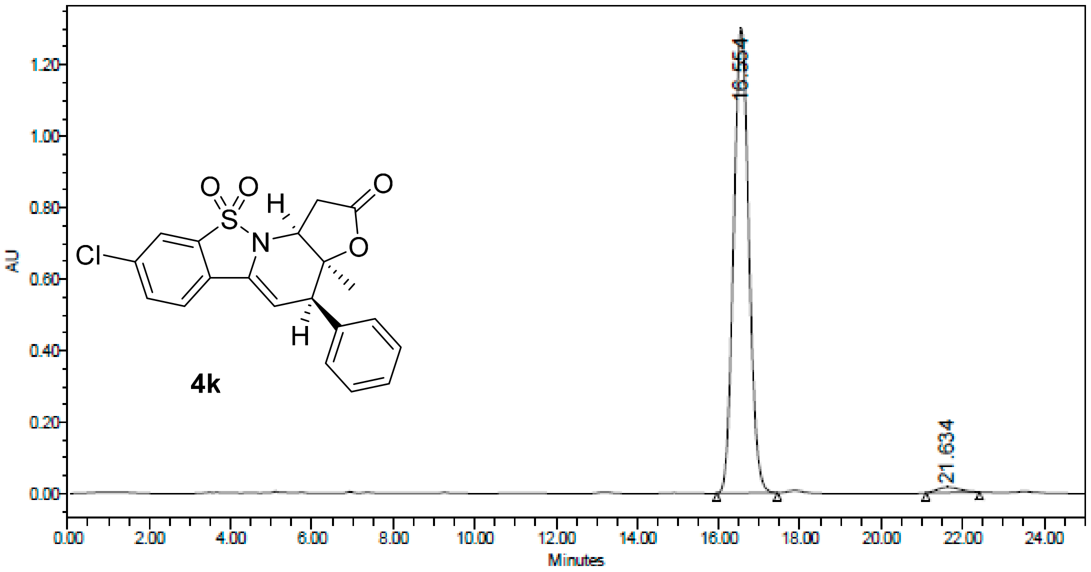

	RT	Area	% Area	Height
1	16.554	33957861	98.34	1301504
2	21.634	574111	1.66	14976

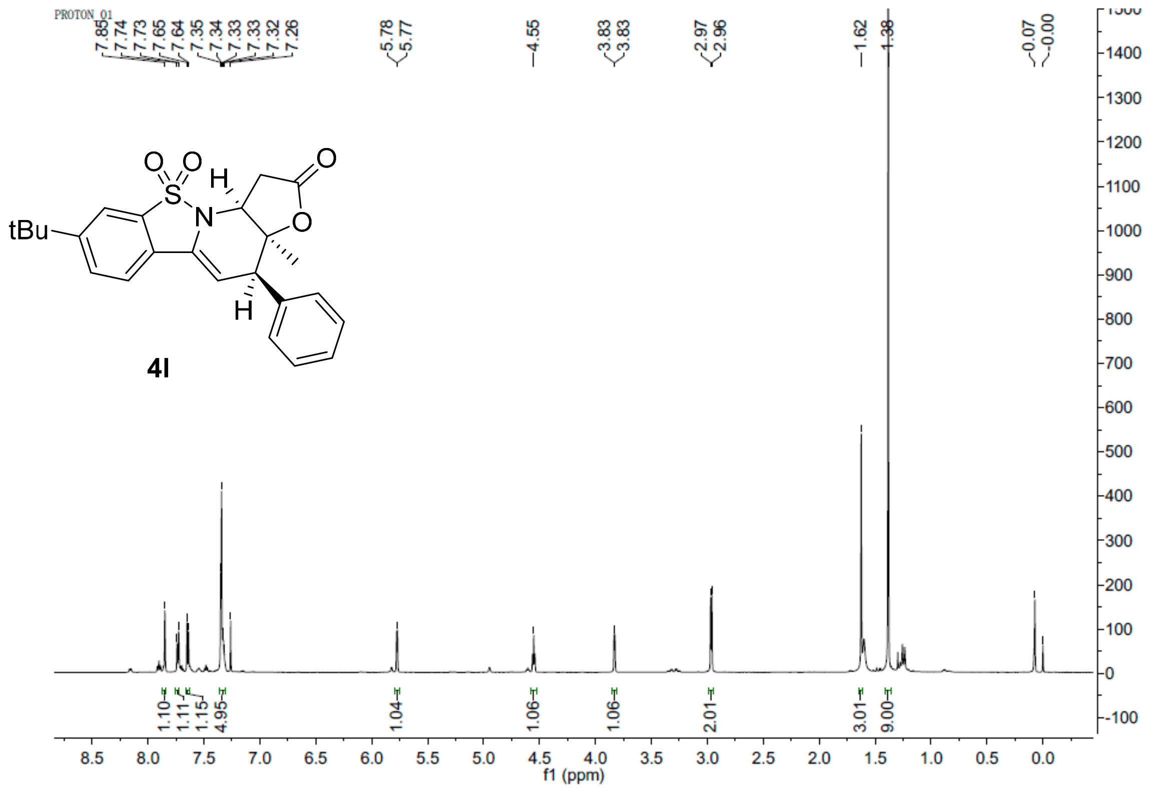
PROTON_01
7.85
7.74
7.73
7.65
7.64
7.35
7.34
7.33
7.33
7.32
7.26
5.78
5.77
4.55
3.83
3.83
2.97
2.96
1.62
1.38
0.07
-0.00
O
O
S
N
H
O
O
tBu
H
4l
1.10
1.11
1.15
4.95
1.04
1.06
1.06
2.01
3.01
9.00
f1 (ppm)

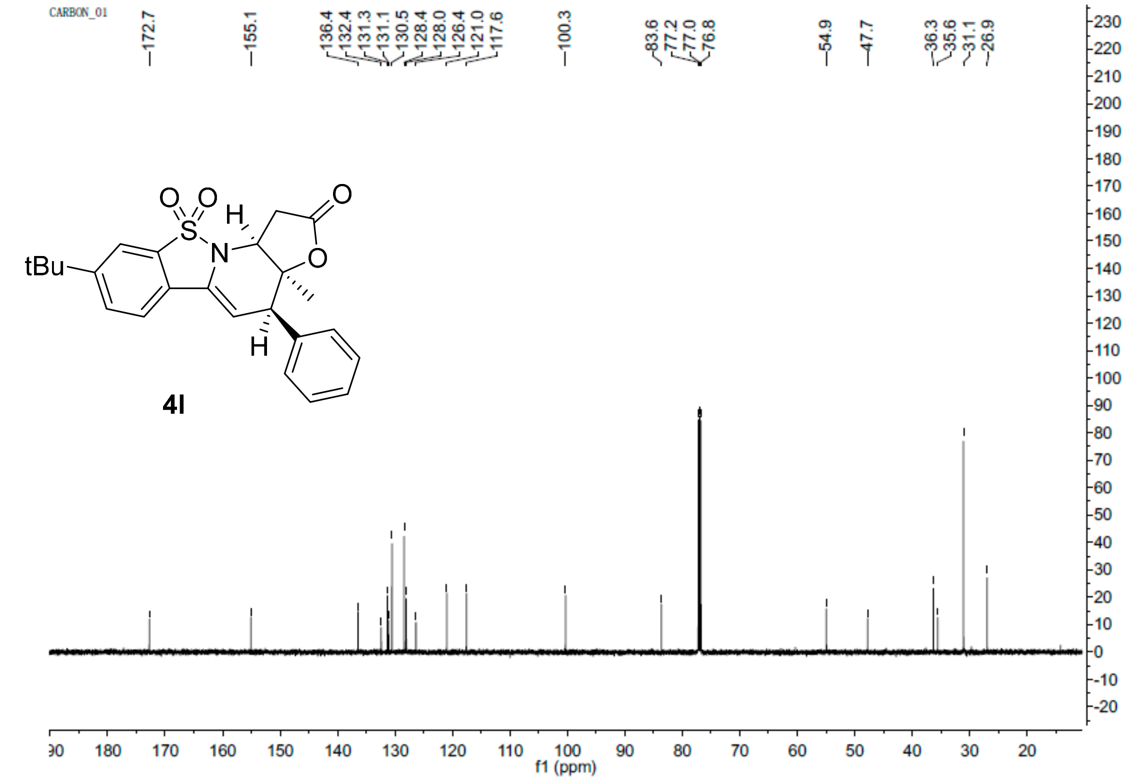
CARBON_01
172.7
155.1
136.4
132.4
131.3
131.1
130.5
128.4
128.0
126.4
121.0
117.6
100.3
83.6
77.2
77.0
76.8
54.9
47.7
36.3
35.6
31.1
26.9
O
O
S
N
H
O
O
tBu
H
4l
f1 (ppm)

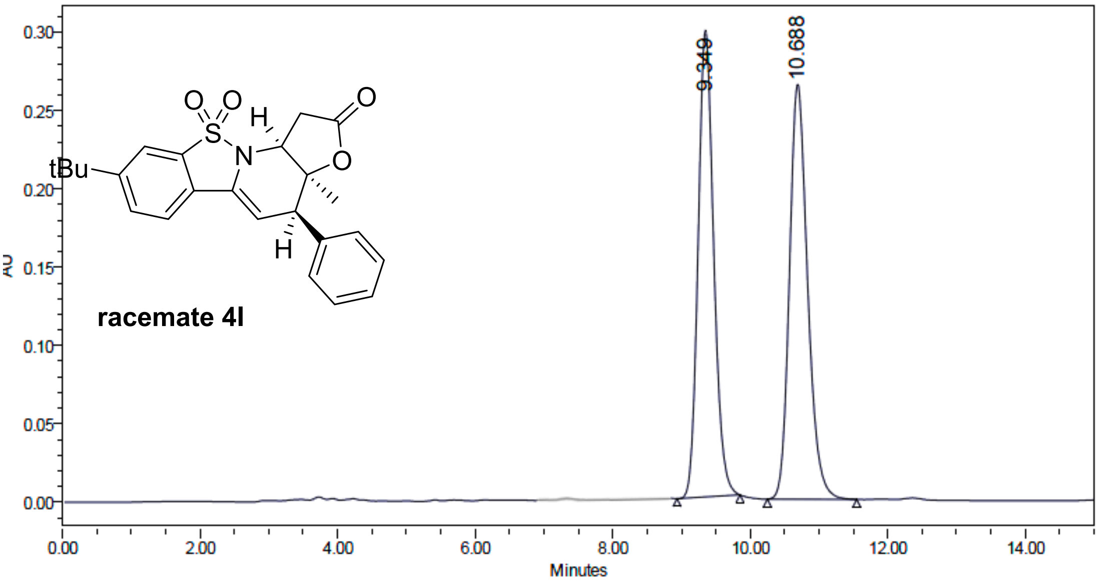

	RT	Area	% Area	Height
1	9.349	4698821	49.10	298266
2	10.688	4871030	50.90	265172

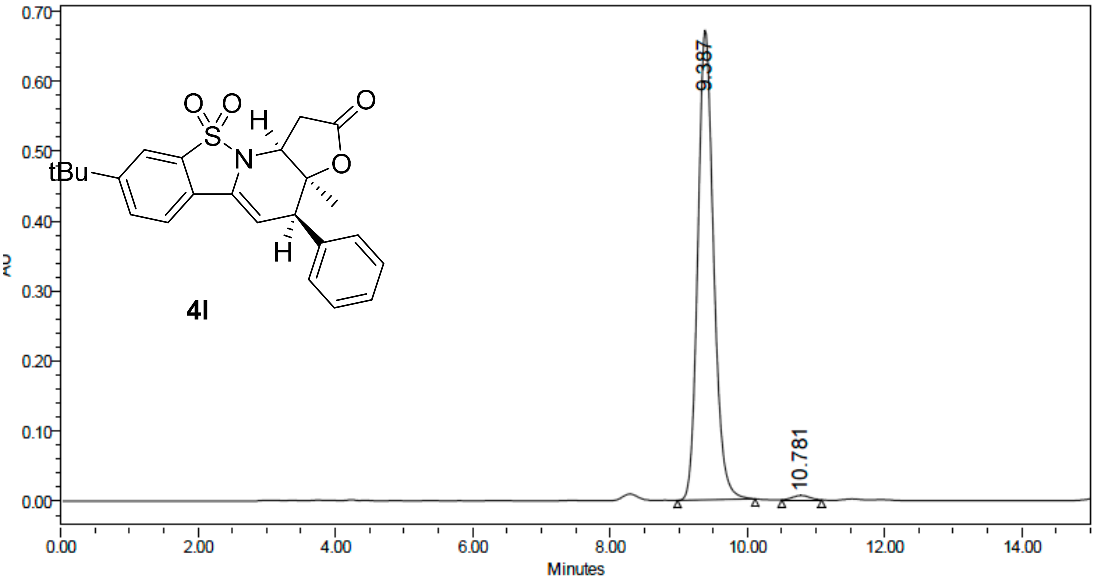

	RT	Area	% Area	Height
1	9.387	10755950	99.01	672355
2	10.781	107179	0.99	6236

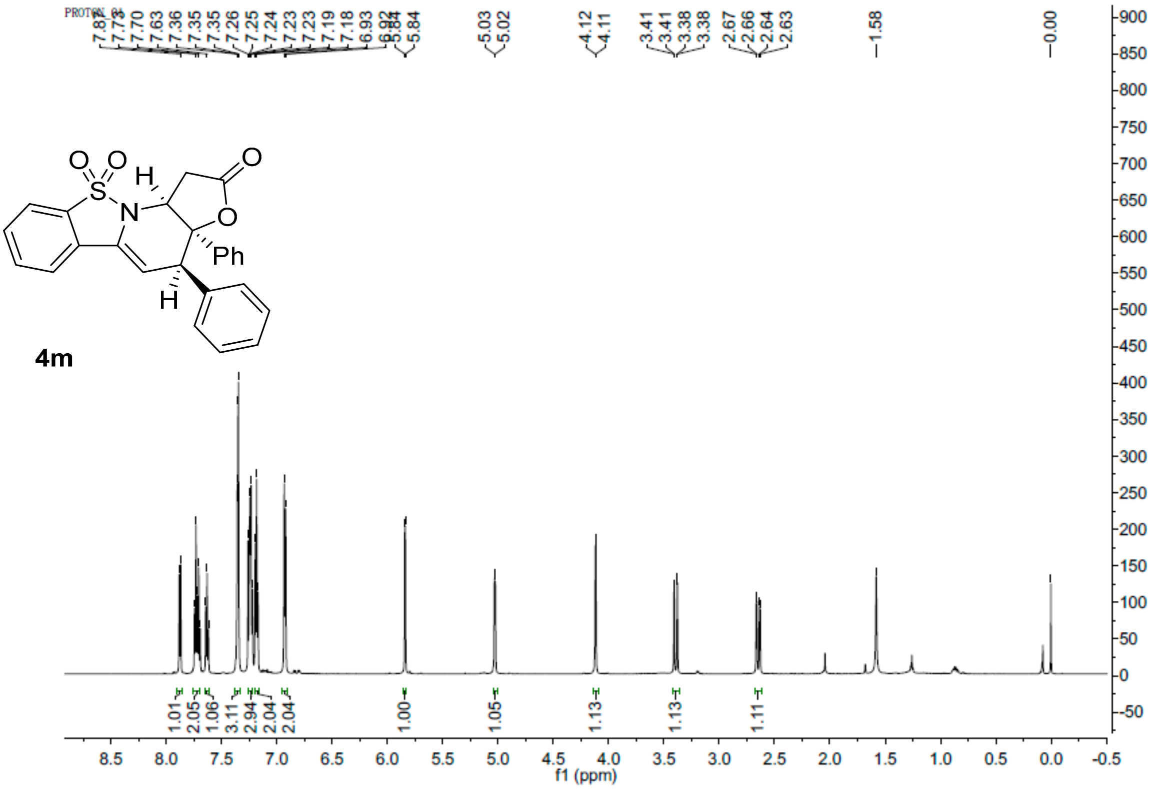
PROTON_01
7.87 7.86 7.73 7.70 7.63 7.36 7.35 7.35 7.26 7.25 7.24 7.23 7.23 7.19 7.18 6.93 6.92 5.84 5.84
5.03 5.02
4.12 4.11
3.41 3.41 3.38 3.38
2.67 2.66 2.64 2.63
1.58
0.00
4m
Ph
1.01 2.05 1.06 3.11 2.94 2.04 2.04 1.00 1.05 1.13 1.13 1.11
f1 (ppm)

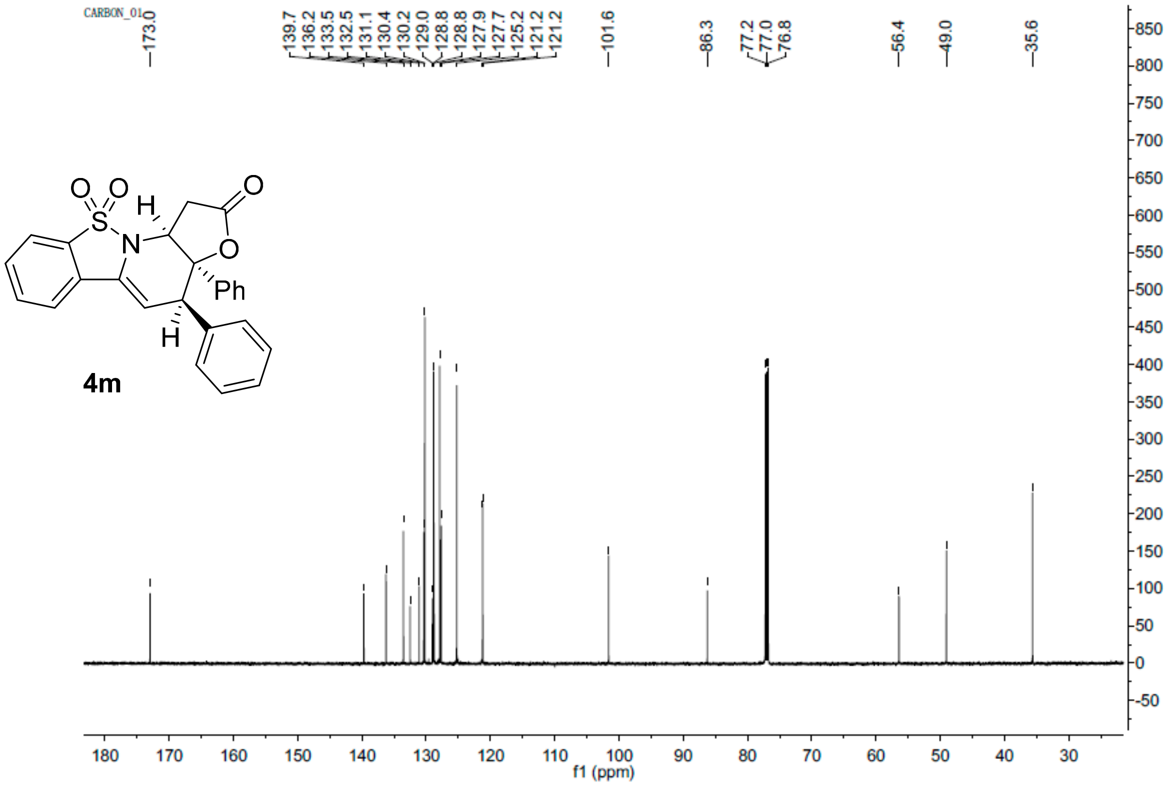
CARBON_01
173.0
139.7 136.2 133.5 132.5 131.1 130.4 130.2 129.0 128.8 128.8 127.9 127.7 125.2 121.2 121.2
101.6
86.3
77.2 77.0 76.8
56.4
49.0
35.6
4m
Ph
f1 (ppm)

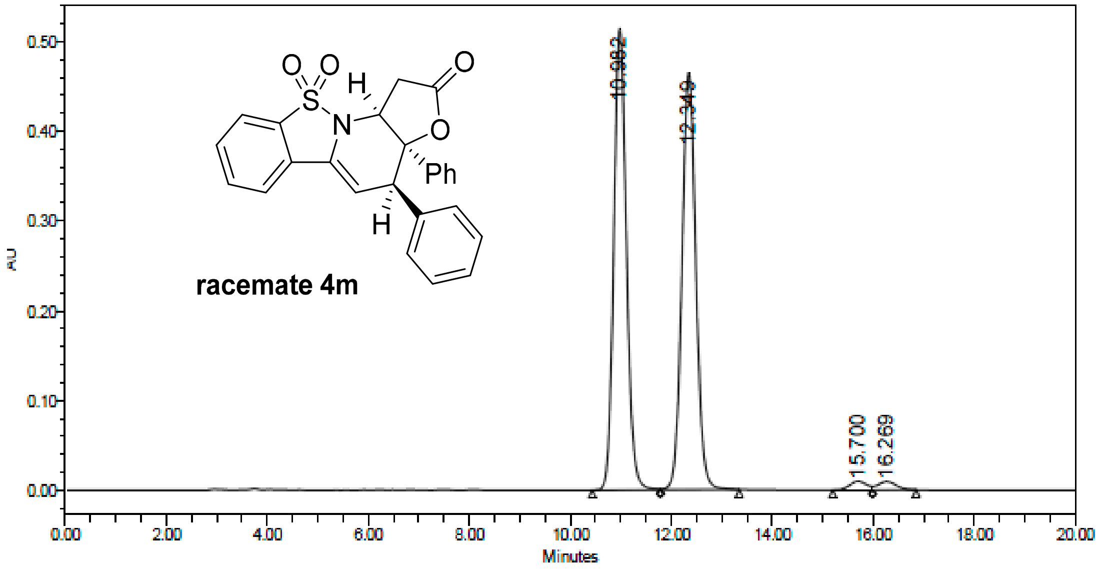

	RT	Area	% Area	Height
1	10.982	8871416	48.66	514878
2	12.349	8896318	48.80	464827
3	15.700	227038	1.25	9658
4	16.269	235925	1.29	9316

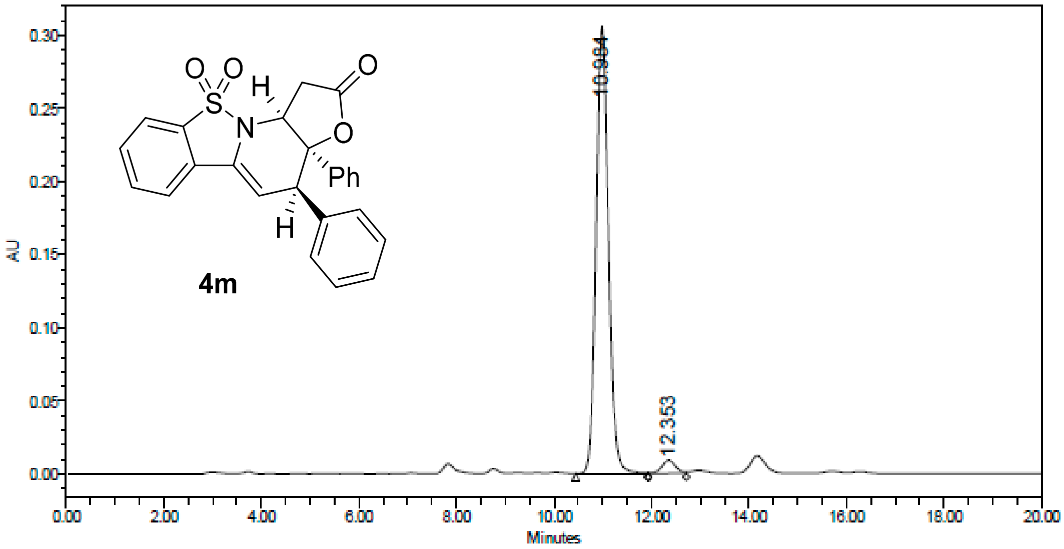

	RT	Area	% Area	Height
1	10.984	5320336	96.83	305045
2	12.353	174016	3.17	8846

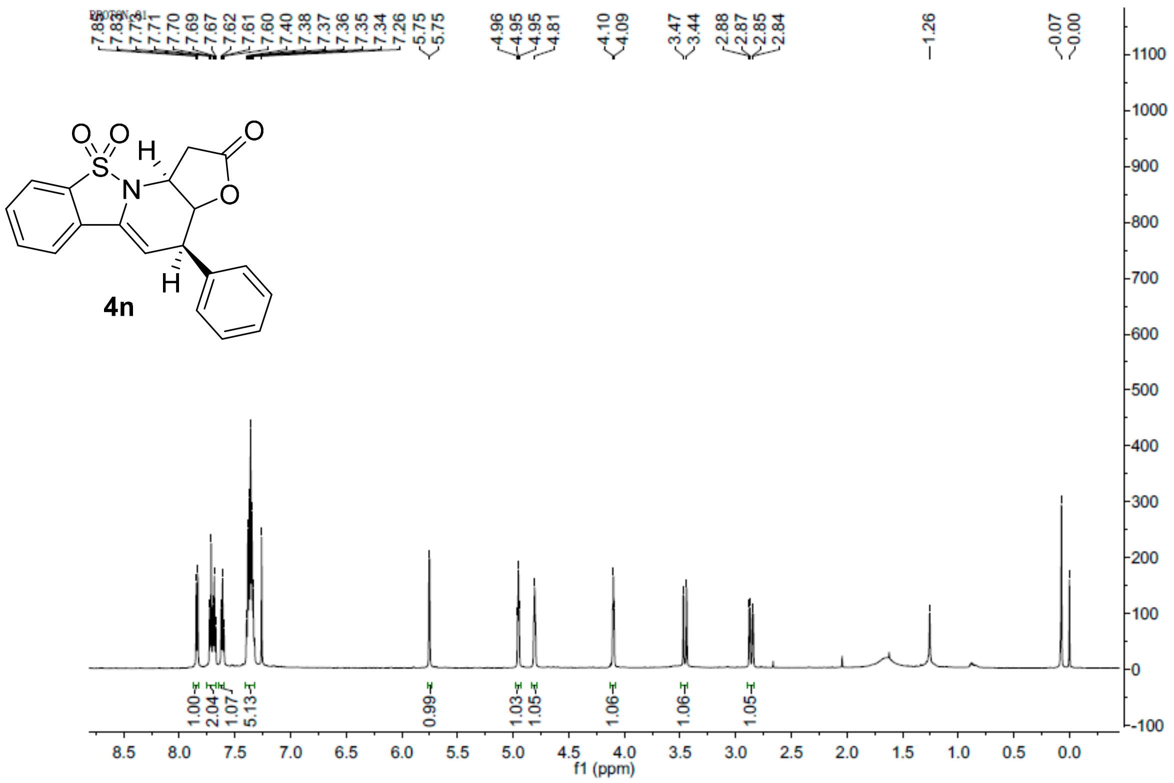

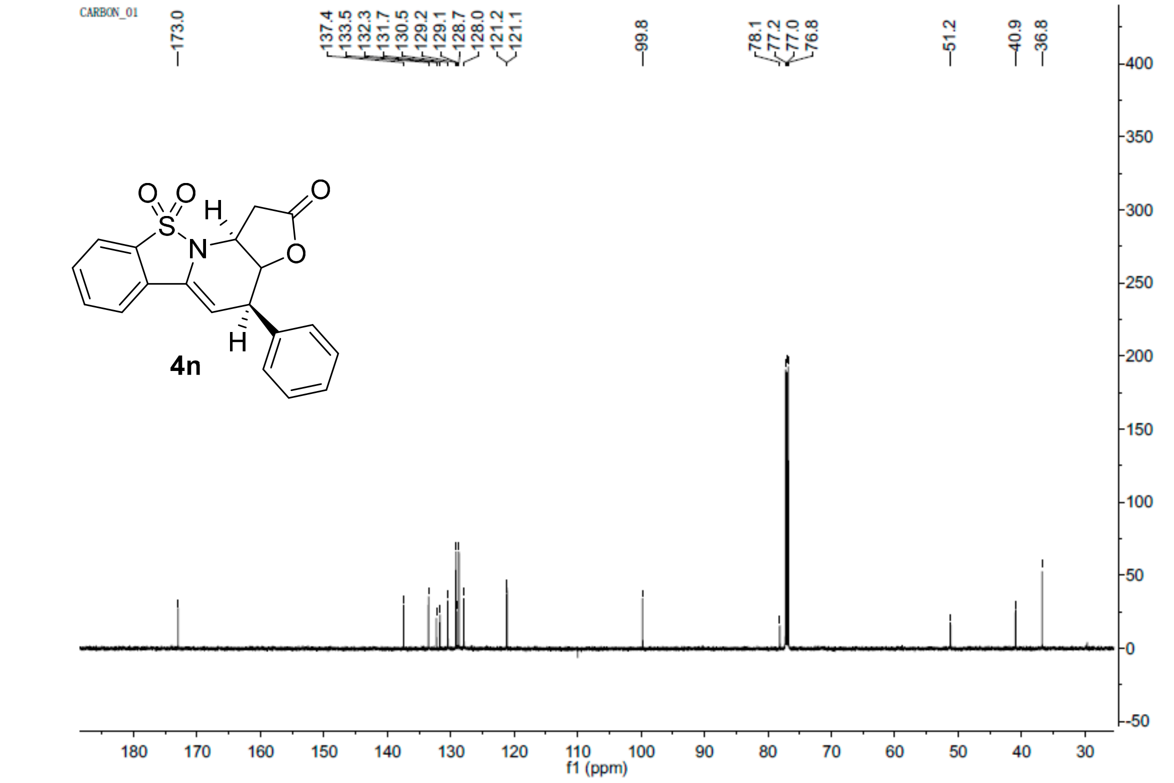

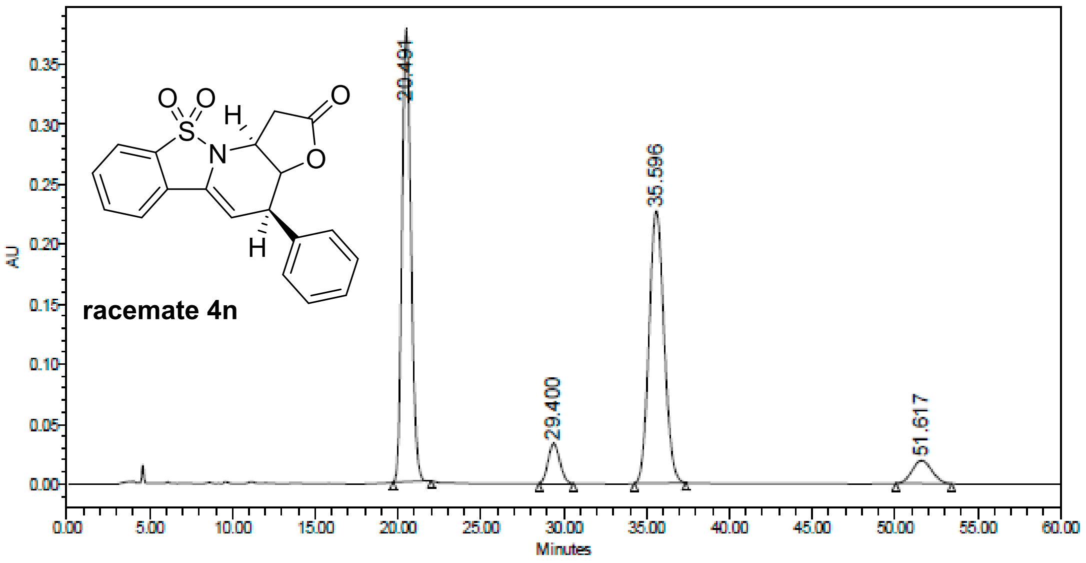

	RT	Area	% Area	Height
1	20.491	14232480	44.84	377691
2	29.400	1630739	5.14	33314
3	35.596	14277406	44.98	227085
4	51.617	1603183	5.05	19330

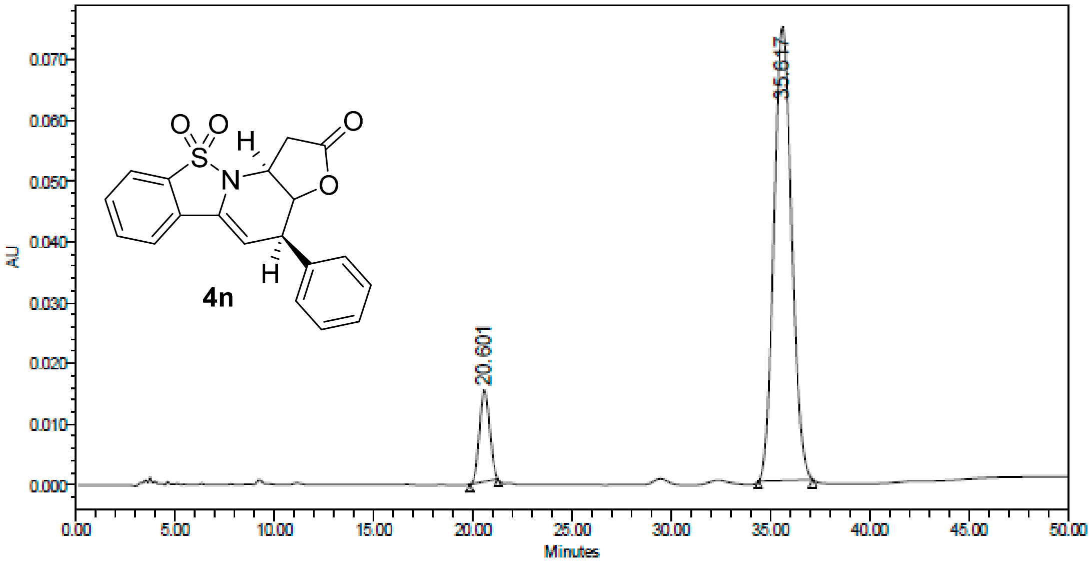

	RT	Area	% Area	Height
1	20.601	536588	10.35	15013
2	35.617	4650127	89.65	74588

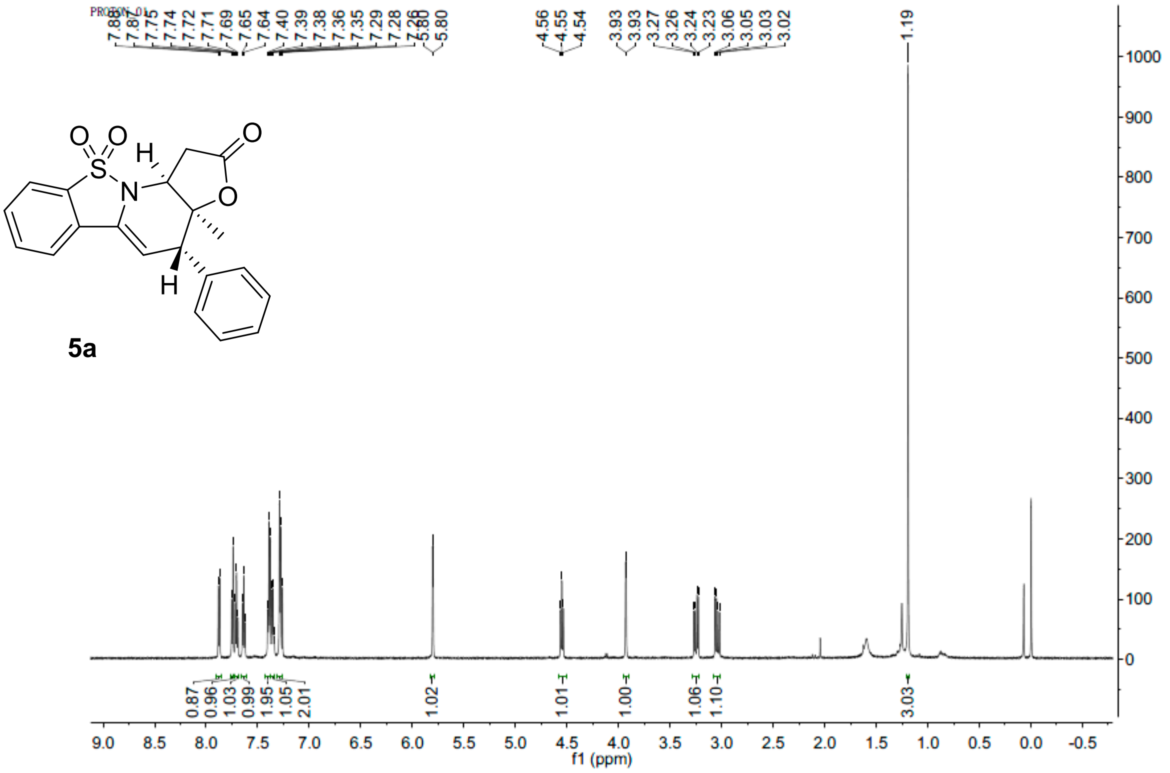
PROTON_01
7.87
7.75
7.74
7.72
7.71
7.69
7.65
7.64
7.40
7.39
7.38
7.36
7.35
7.29
7.28
5.80
4.56
4.55
4.54
3.93
3.93
3.27
3.26
3.24
3.23
3.06
3.05
3.03
3.02
1.19
0.87
0.96
1.03
0.99
1.95
1.05
2.01
1.02
1.01
1.00
1.06
1.10
3.03
f1 (ppm)

5a

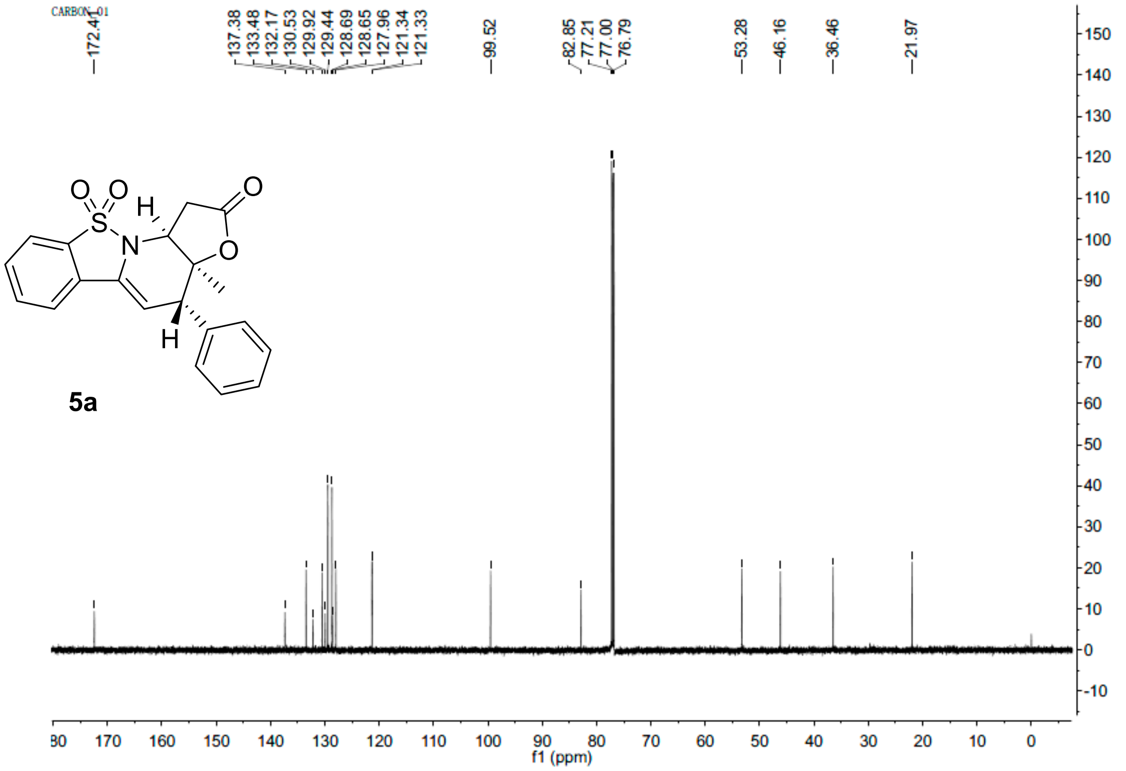
CARBON_01
172.41
137.38
133.48
132.17
130.53
129.92
129.44
128.69
128.65
127.96
121.34
121.33
99.52
82.85
77.21
77.00
76.79
53.28
46.16
36.46
21.97
f1 (ppm)

5a

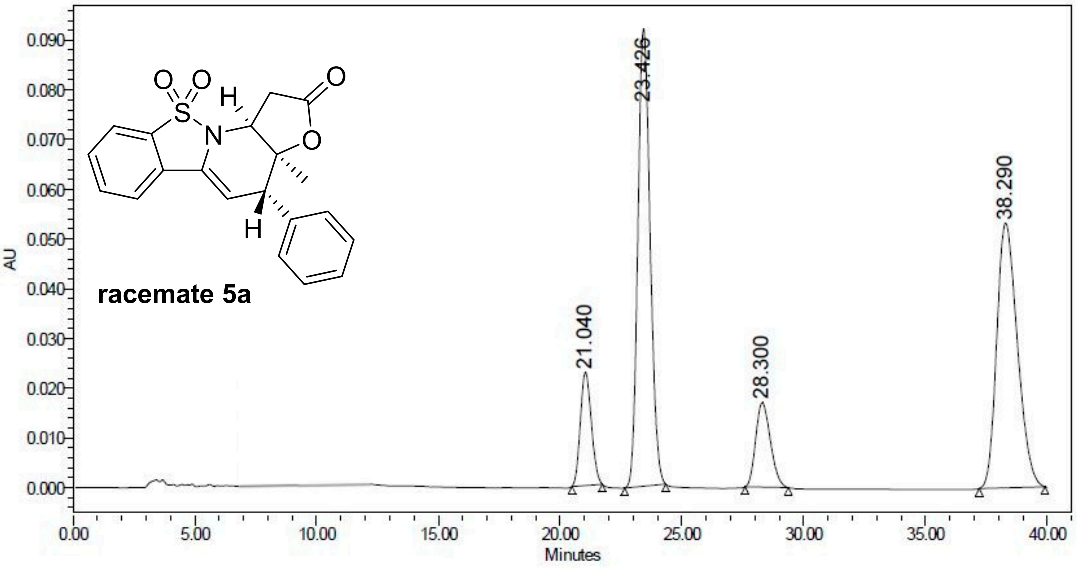

	RT	Area	% Area	Height
1	21.040	715013	9.18	22797
2	23.426	3212990	41.27	92060
3	28.300	716262	9.20	16994
4	38.290	3141649	40.35	53265

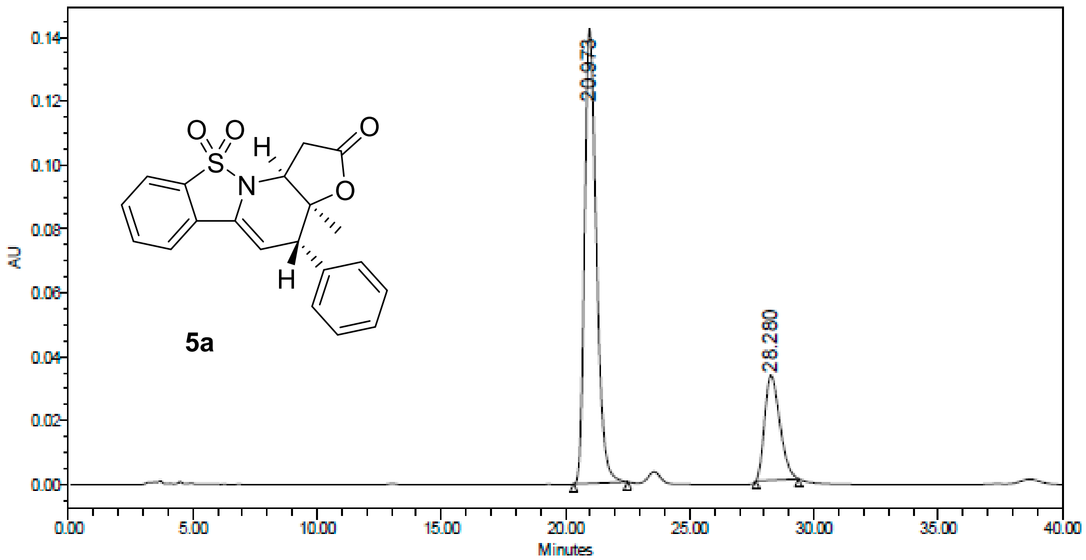

	RT	Area	% Area	Height
1	20.973	4706598	77.12	142165
2	28.280	1396014	22.88	32668

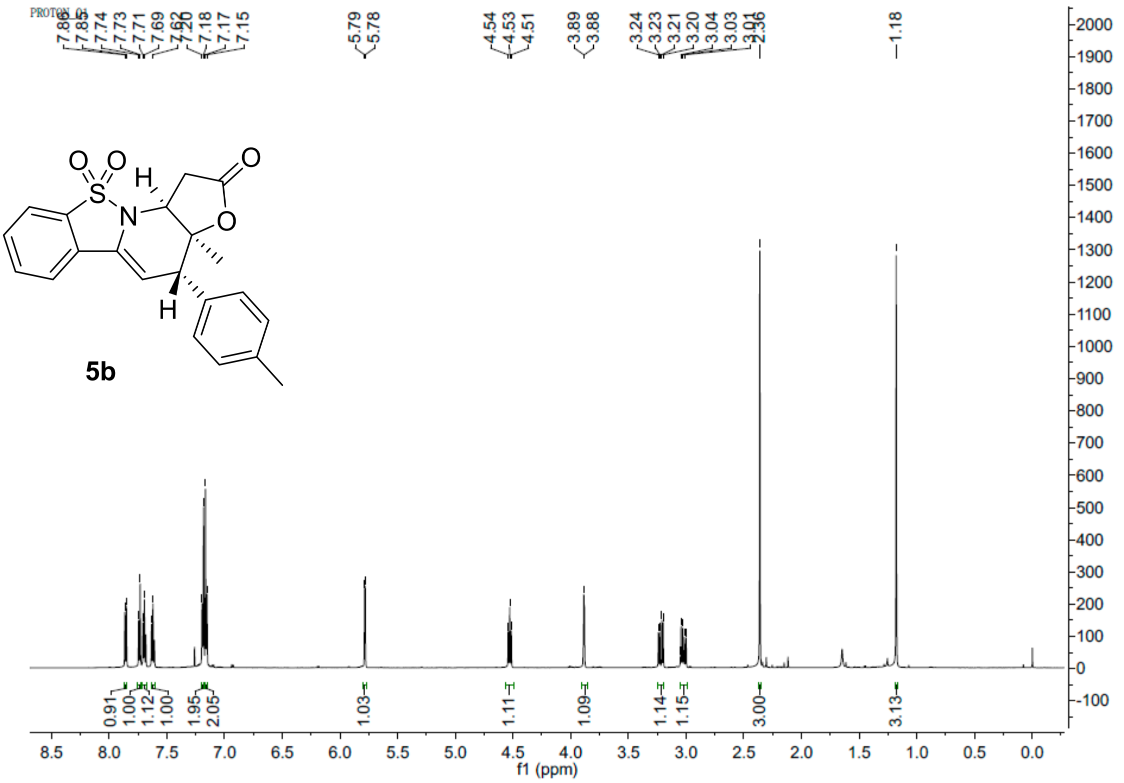
PROTON_01
5b
f1 (ppm)

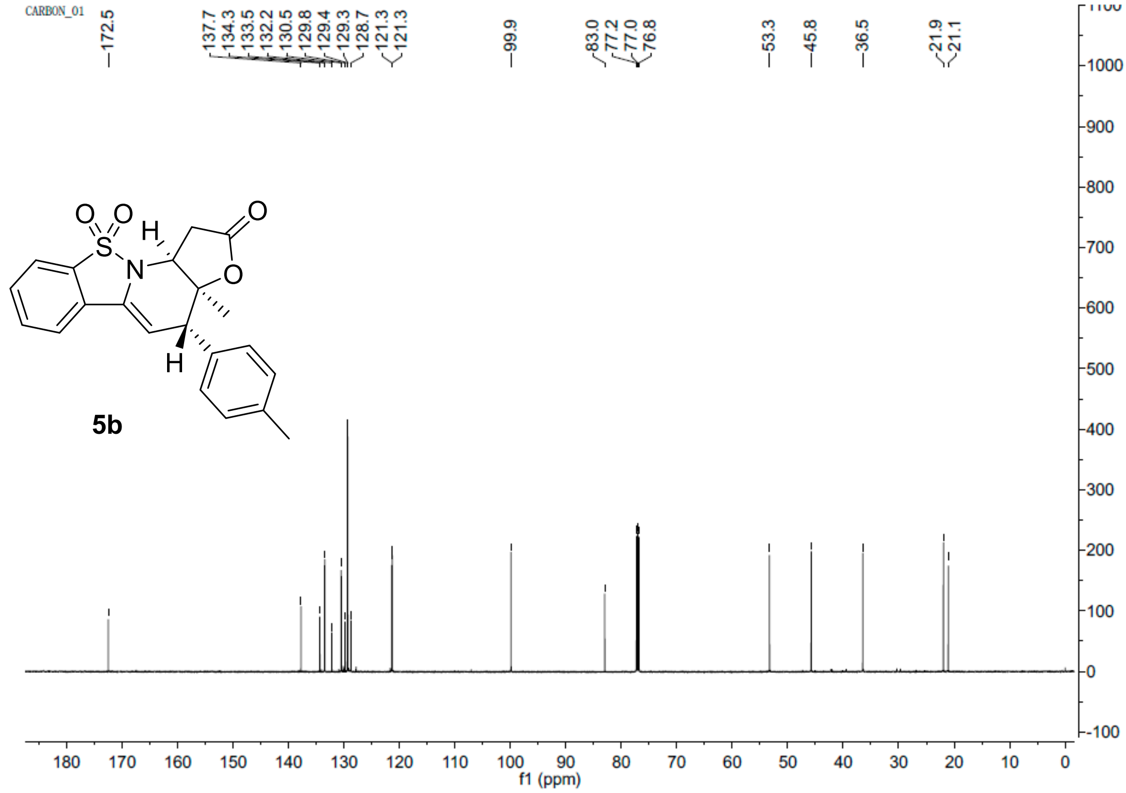
CARBON_01
5b
f1 (ppm)

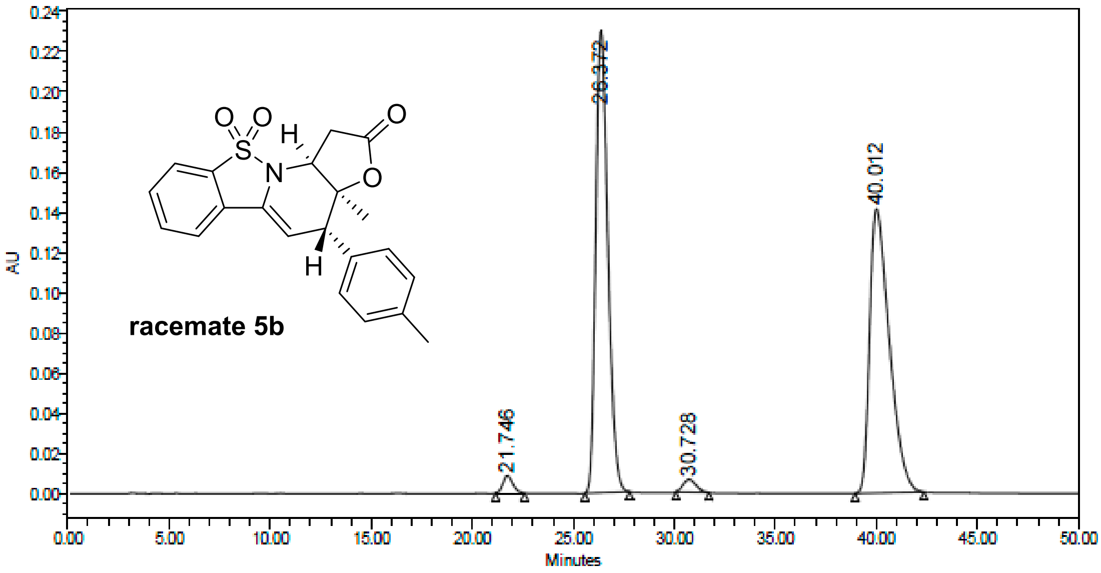

	RT	Area	% Area	Height
1	21.746	298751	1.52	8549
2	26.372	9539691	48.49	229589
3	30.728	283220	1.44	6299
4	40.012	9550840	48.55	141247

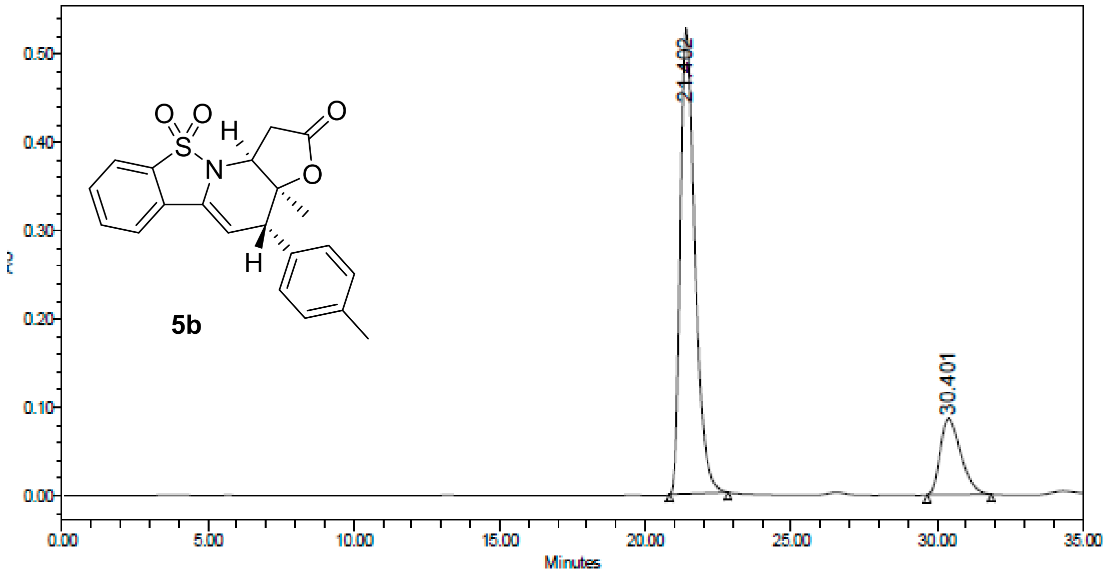

	RT	Area	% Area	Height
1	21.402	18484106	81.34	527128
2	30.401	4239659	18.66	85597

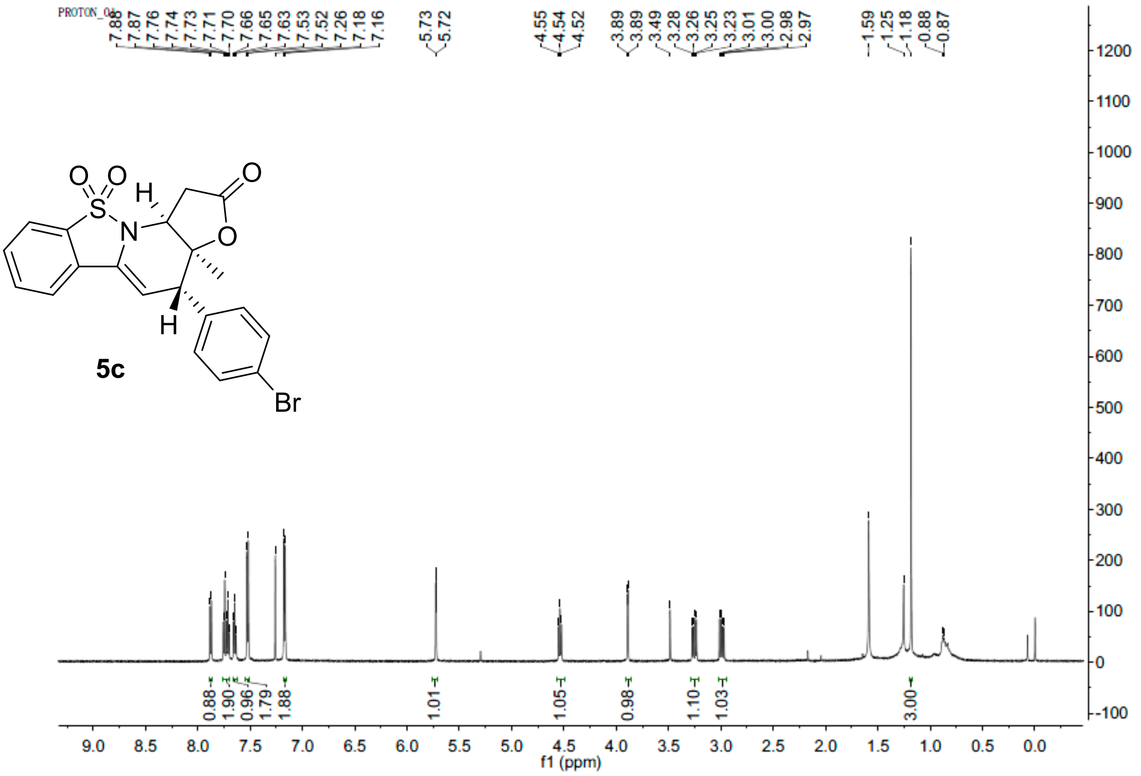
PROTON_01
7.88
7.87
7.76
7.74
7.73
7.71
7.70
7.66
7.65
7.63
7.53
7.52
7.26
7.18
7.16
5.73
5.72
4.55
4.54
4.52
3.89
3.89
3.49
3.28
3.26
3.25
3.23
3.01
3.00
2.98
2.97
1.59
1.25
1.18
0.88
0.87
5c
Br
0.88
1.90
0.96
1.79
1.88
1.01
1.05
0.98
1.10
1.03
3.00
f1 (ppm)

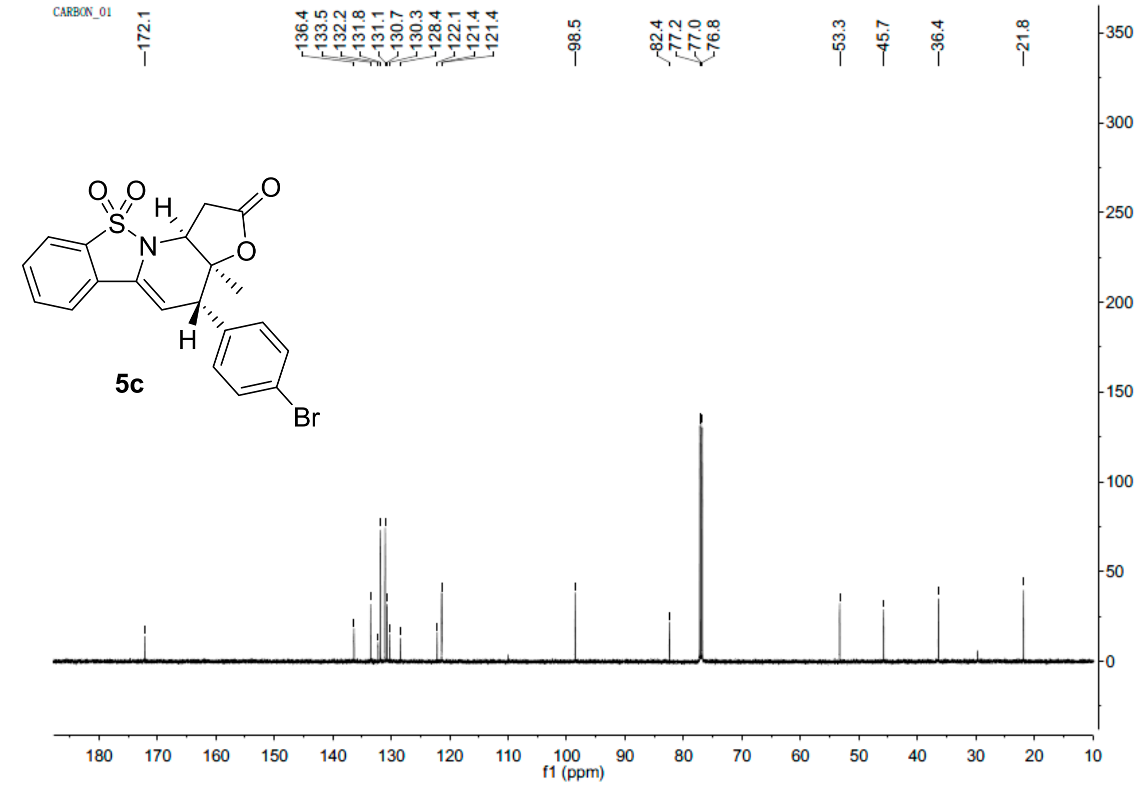
CARBON_01
172.1
136.4
133.5
132.2
131.8
131.1
130.7
130.3
128.4
122.1
121.4
121.4
98.5
82.4
77.2
77.0
76.8
53.3
45.7
36.4
21.8
5c
Br
f1 (ppm)

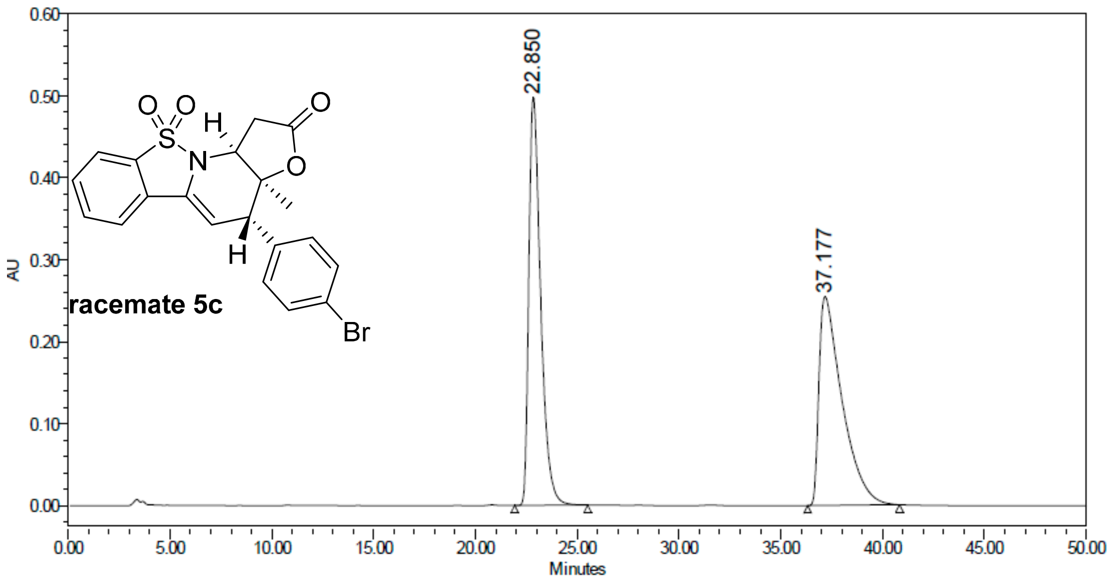

	RT	Area	% Area	Height
1	22.850	20133558	50.05	497551
2	37.177	20091897	49.95	254692

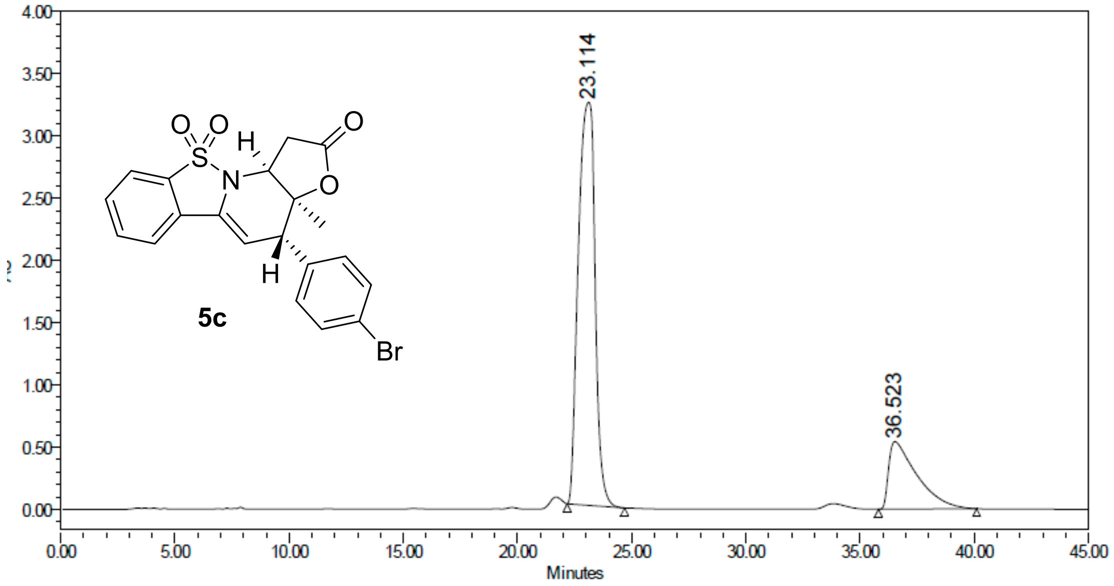

	RT	Area	% Area	Height
1	23.114	165666082	78.00	3237204
2	36.523	46722257	22.00	543690

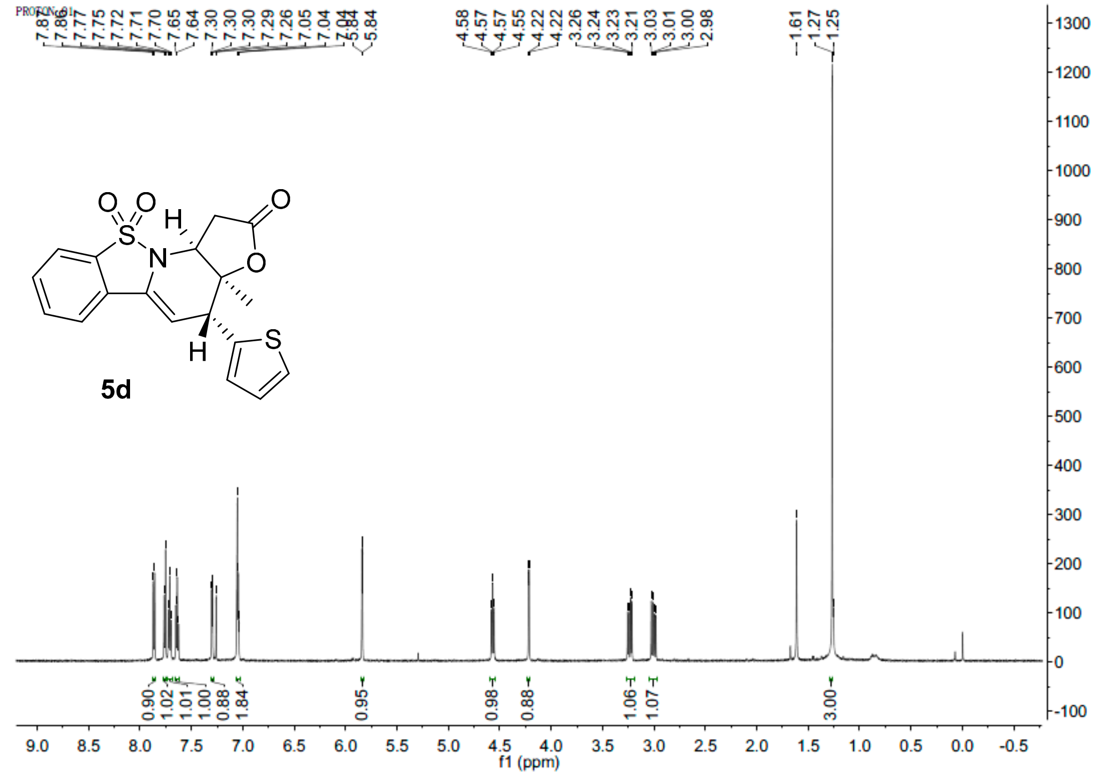
PROTON_01
7.87 7.86 7.77 7.75 7.72 7.71 7.70 7.65 7.64 7.30 7.30 7.30 7.29 7.26 7.05 7.04
5.84
4.58 4.57 4.57 4.55 4.22 4.22 3.26 3.24 3.23 3.21 3.03 3.01 3.00 2.98
1.61 1.27 1.25
5d
0.90 1.02 1.01 1.00 0.88 1.84 0.95 0.98 0.88 1.06 1.07 3.00
f1 (ppm)

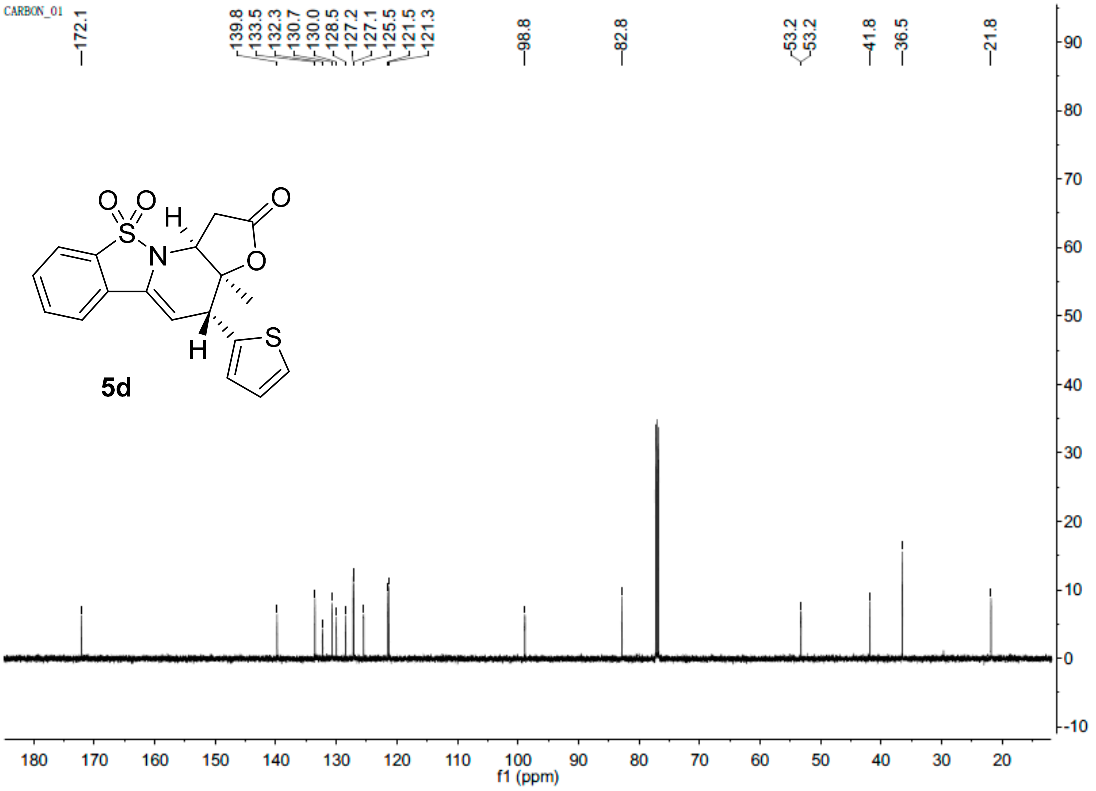
CARBON_01
172.1
139.8 133.5 132.3 130.7 130.0 128.5 127.2 127.1 125.5 121.5 121.3
98.8
82.8
53.2 53.2
41.8 36.5
21.8
5d
f1 (ppm)

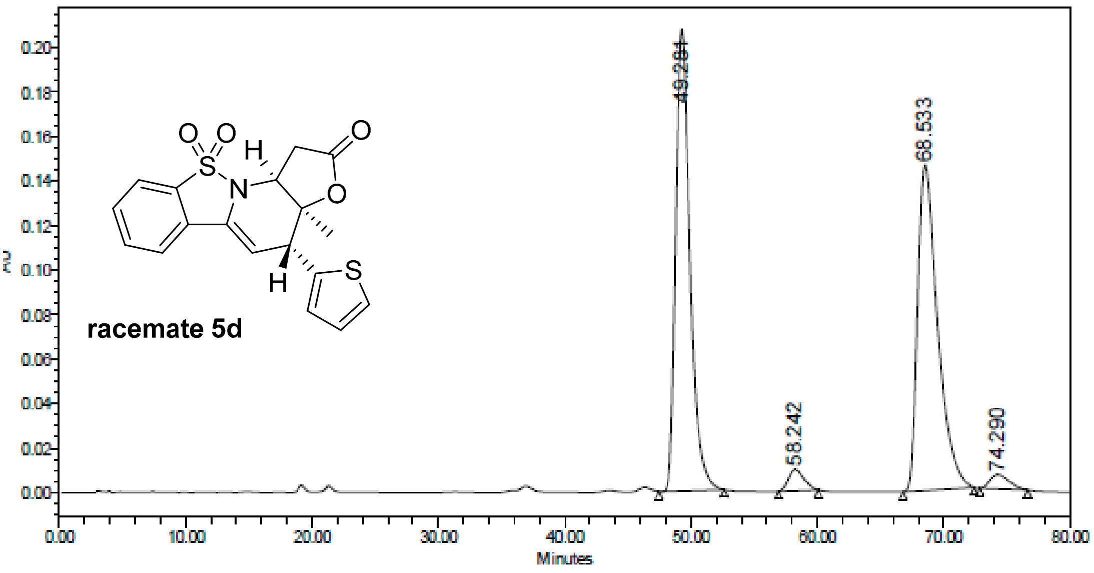

	RT	Area	% Area	Height
1	49.281	16380653	48.23	207160
2	58.242	808300	2.38	9421
3	68.533	16107054	47.43	146183
4	74.290	666806	1.96	6305

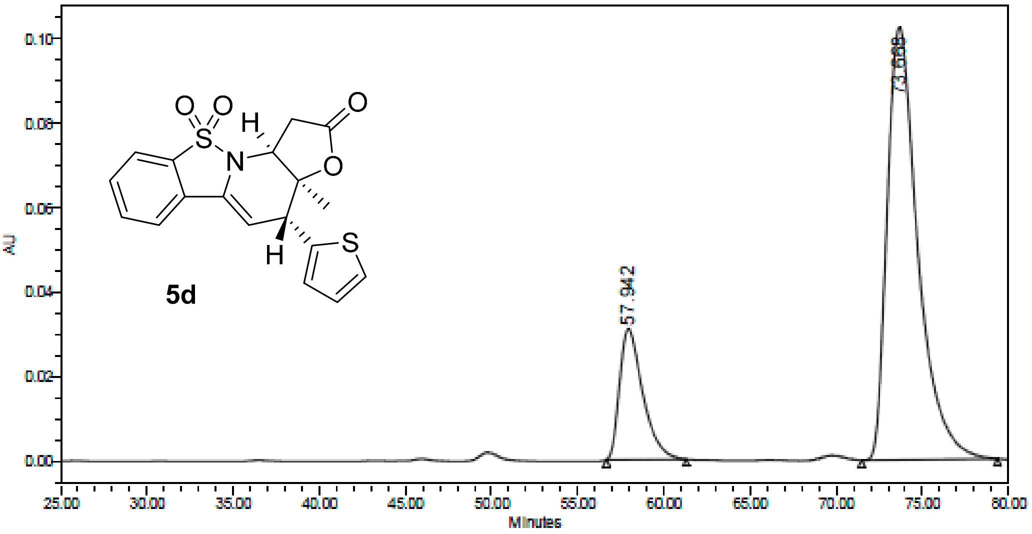

	RT	Area	% Area	Height
1	57.942	2894642	18.58	30941
2	73.668	12687729	81.42	102286

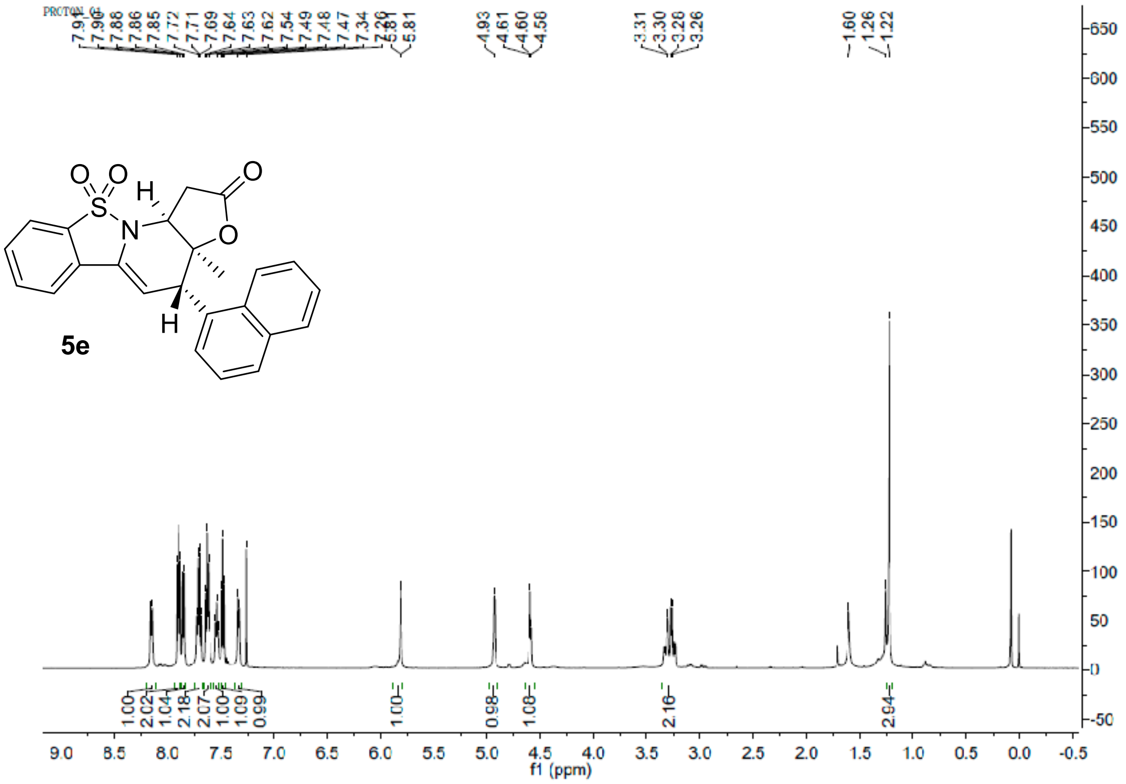
PROTON_01
7.91 7.90 7.88 7.86 7.85 7.72 7.71 7.69 7.64 7.63 7.62 7.54 7.49 7.48 7.47 7.34 7.26 5.81 5.81
4.93 4.61 4.60 4.58
3.31 3.30 3.28 3.26
1.60 1.26 1.22
5e
1.00 2.02 1.04 2.18 2.07 1.00 1.09 0.99
1.00
0.98
1.08
2.16
2.94
f1 (ppm)

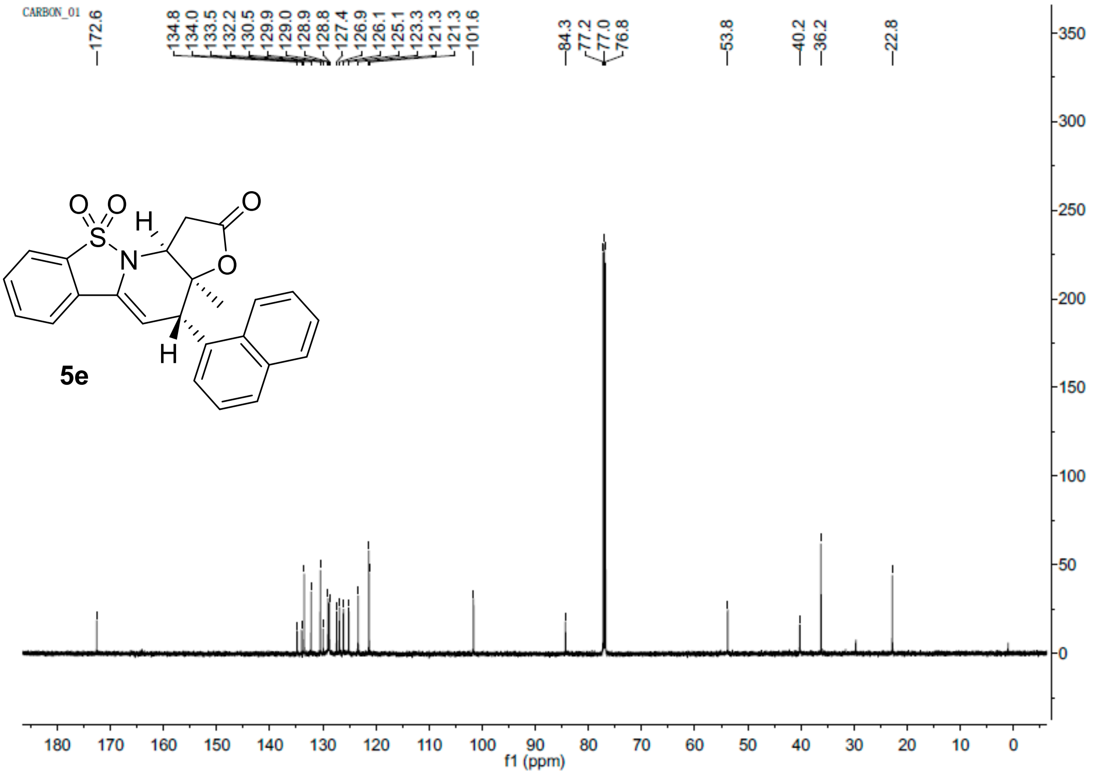
CARBON_01
172.6
134.8 134.0 133.5 132.2 130.5 129.9 129.0 128.9 128.8 127.4 126.9 126.1 125.1 123.3 121.3 121.3 101.6
84.3 77.2 77.0 76.8
53.8
40.2 36.2
22.8
5e
f1 (ppm)

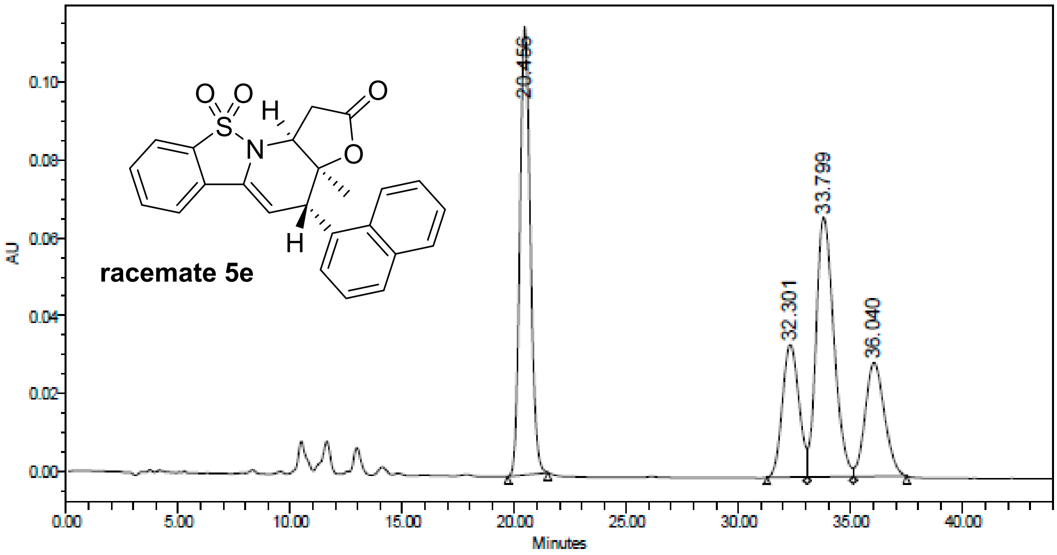

	RT	Area	% Area	Height
1	20.456	3723779	34.19	114965
2	32.301	1744441	16.02	34017
3	33.799	3670741	33.70	66507
4	36.040	1752980	16.09	29183

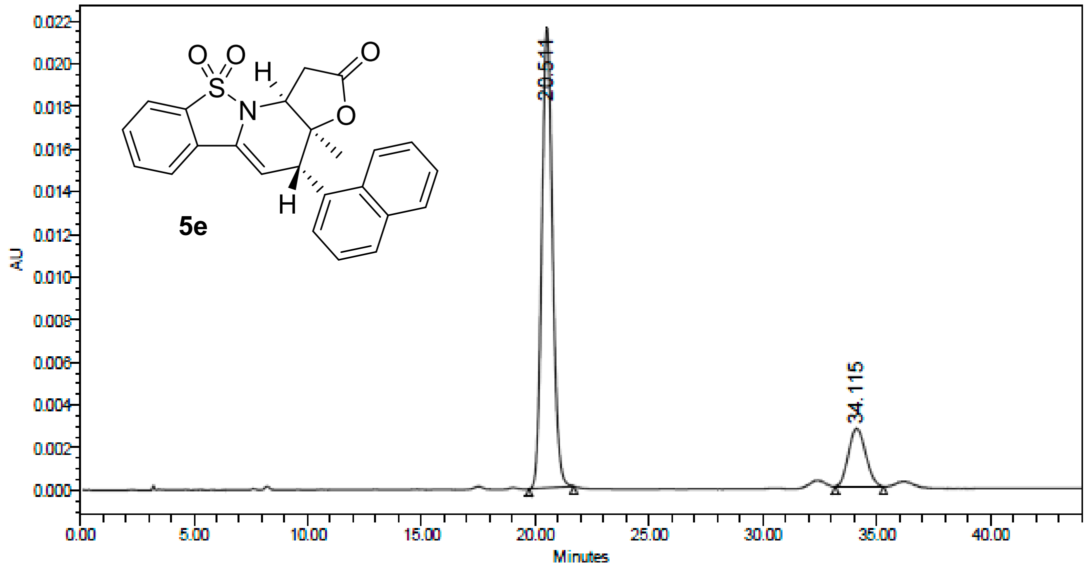

	RT	Area	% Area	Height
1	20.511	705440	83.11	21595
2	34.115	143350	16.89	2729

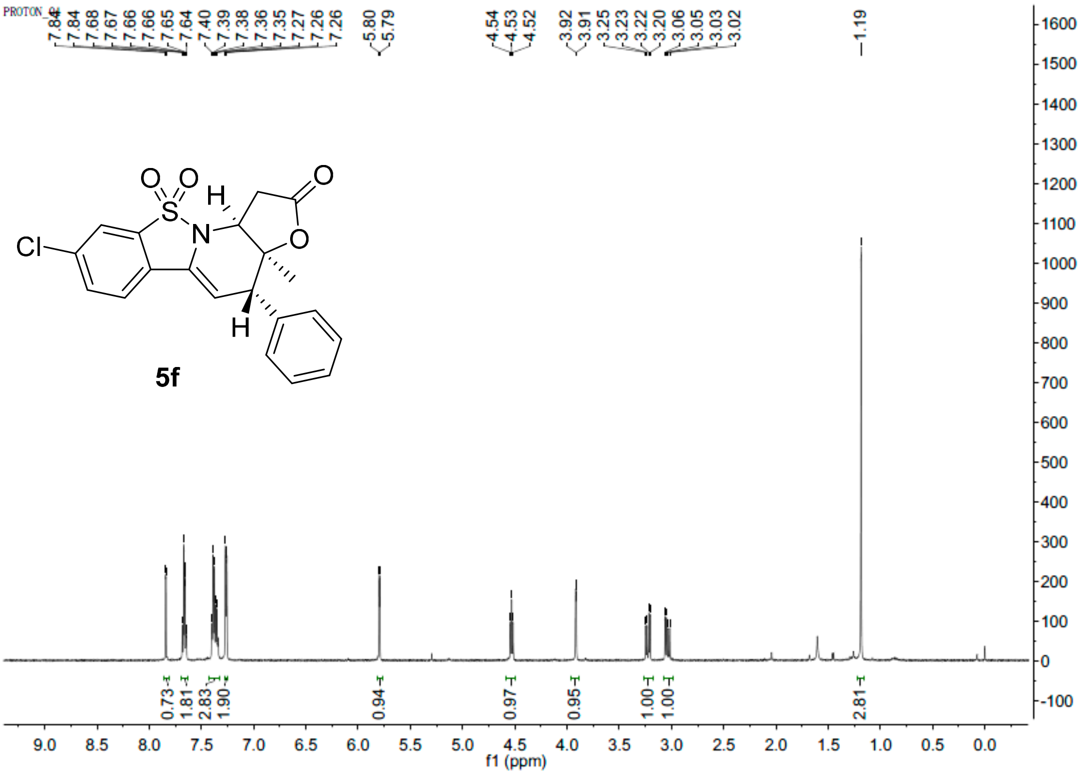
PROTON_01
7.84
7.84
7.68
7.67
7.66
7.66
7.65
7.64
7.40
7.39
7.38
7.36
7.35
7.27
7.26
7.26
5.80
5.79
4.54
4.53
4.52
3.92
3.91
3.25
3.23
3.22
3.20
3.06
3.05
3.03
3.02
1.19
Cl
S
O
O
N
H
O
O
H
5f
0.73
1.81
2.83
1.90
0.94
0.97
0.95
1.00
1.00
2.81
f1 (ppm)

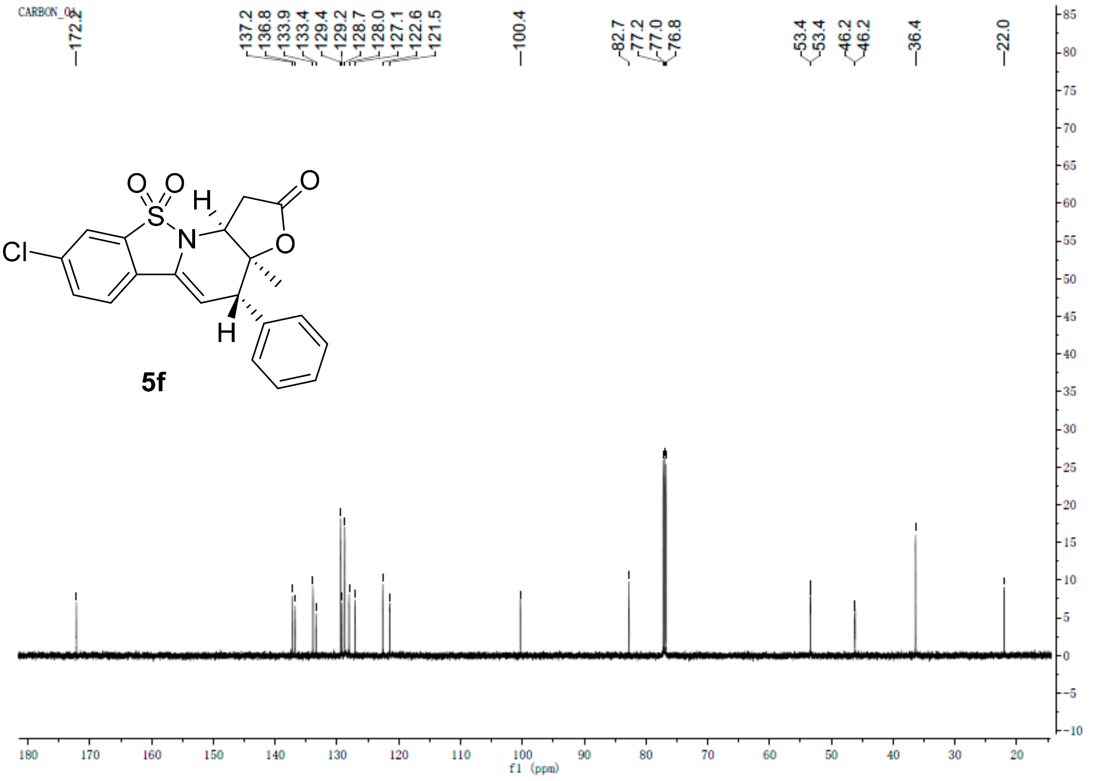
CARBON_01
172.2
137.2
136.8
133.9
133.4
129.4
129.2
128.7
128.0
127.1
122.6
121.5
100.4
82.7
77.2
77.0
76.8
53.4
53.4
46.2
46.2
36.4
22.0
Cl
S
O
O
N
H
O
O
H
5f
f1 (ppm)

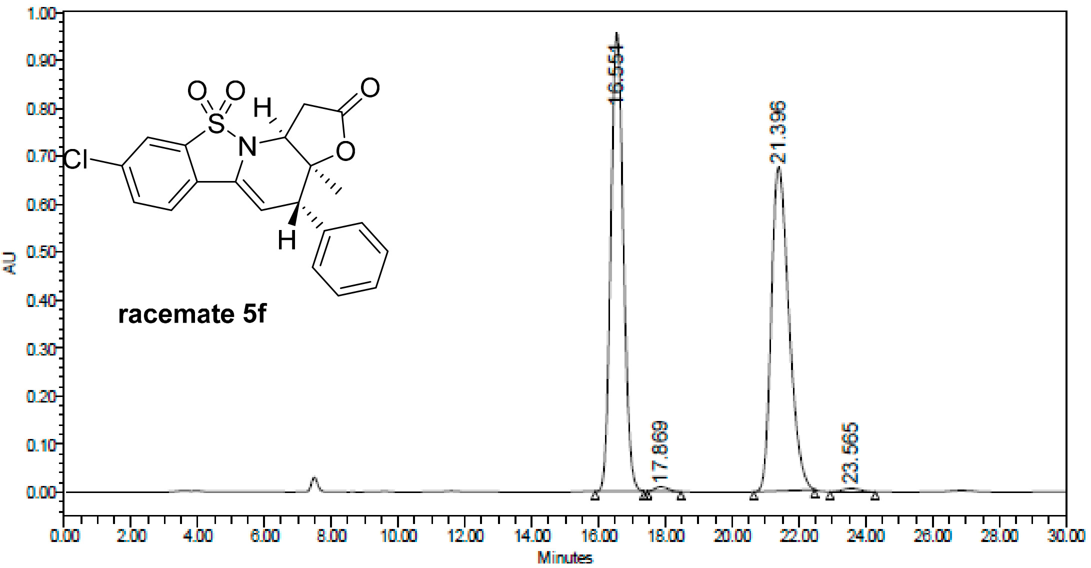

	RT	Area	% Area	Height
1	16.551	24740656	49.45	954355
2	17.869	255013	0.51	9600
3	21.396	24802676	49.57	675931
4	23.565	232287	0.46	6382

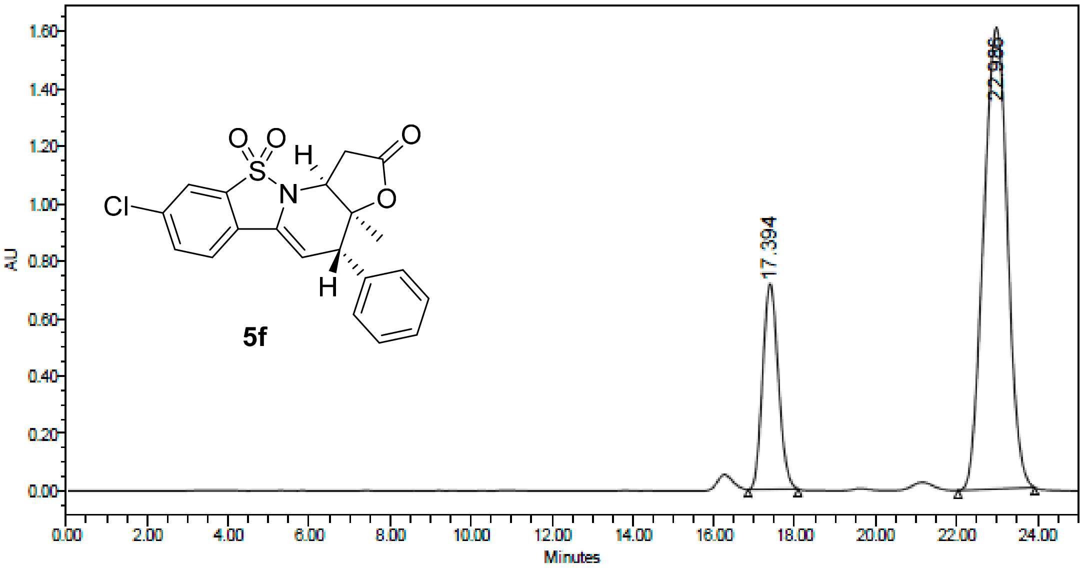

	RT	Area	% Area	Height
1	17.394	19144128	22.51	714822
2	22.986	65888719	77.49	1605184

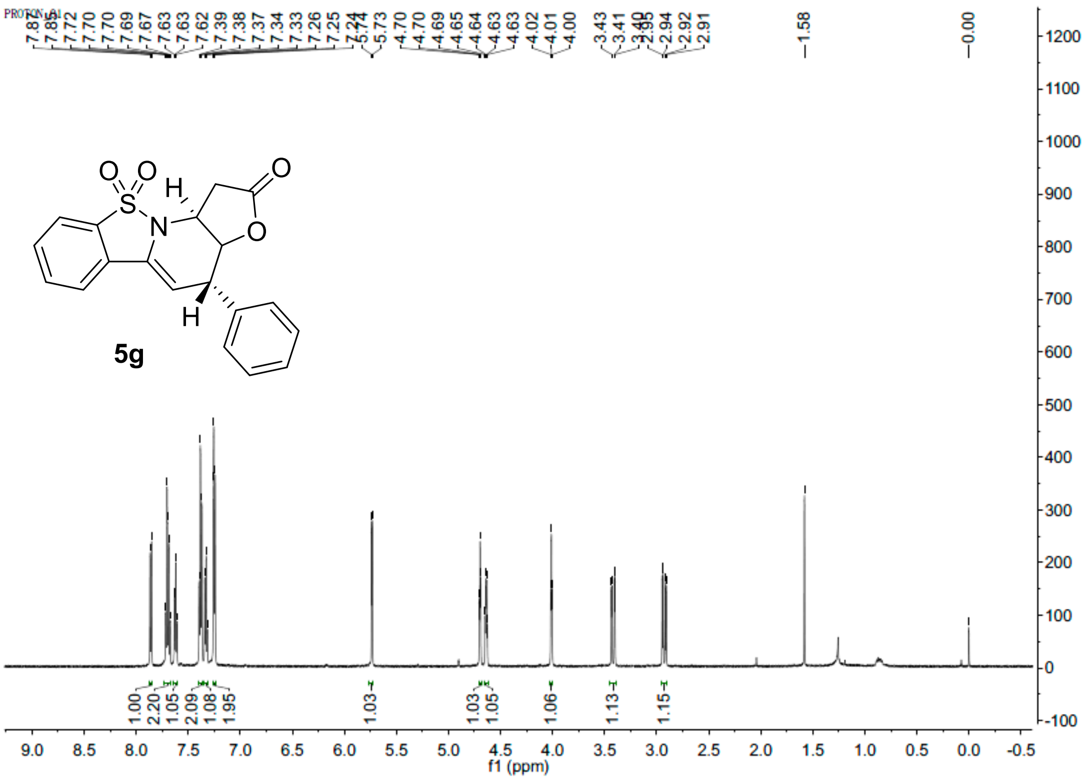
PROTON_01
5g
1.00
2.20
1.05
2.09
1.08
1.95
1.03
1.03
1.05
1.06
1.13
1.15
f1 (ppm)

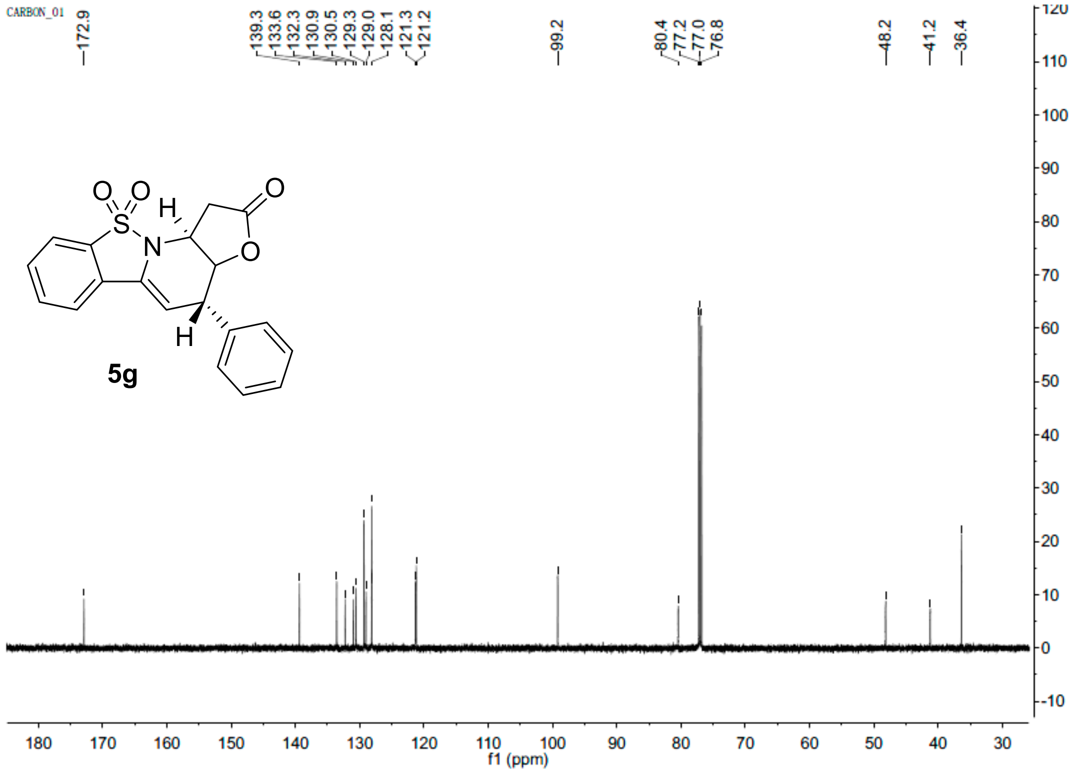
CARBON_01
172.9
139.3
133.6
132.3
130.9
130.5
129.3
129.0
128.1
121.3
121.2
99.2
80.4
77.2
77.0
76.8
48.2
41.2
36.4
5g
f1 (ppm)

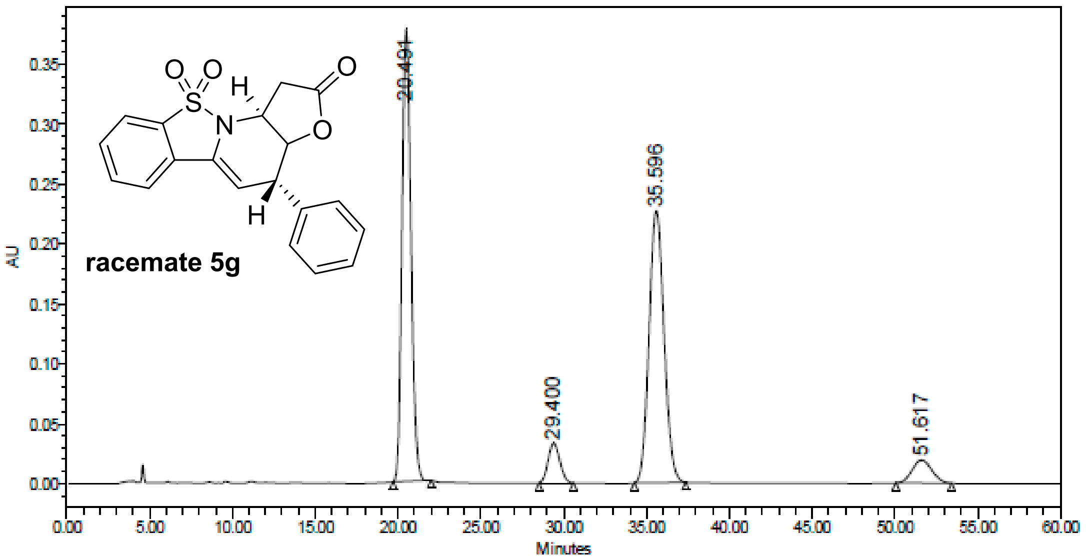

	RT	Area	% Area	Height
1	20.491	14232480	44.84	377691
2	29.400	1630739	5.14	33314
3	35.596	14277406	44.98	227085
4	51.617	1603183	5.05	19330

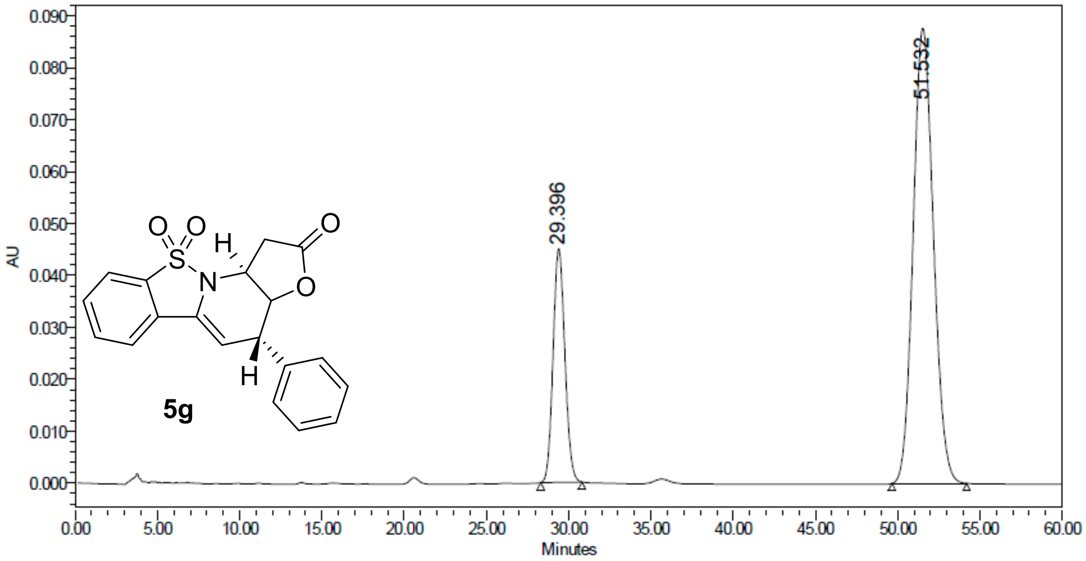

	RT	Area	% Area	Height
1	29.396	2245952	23.02	45059
2	51.532	7511547	76.98	87662

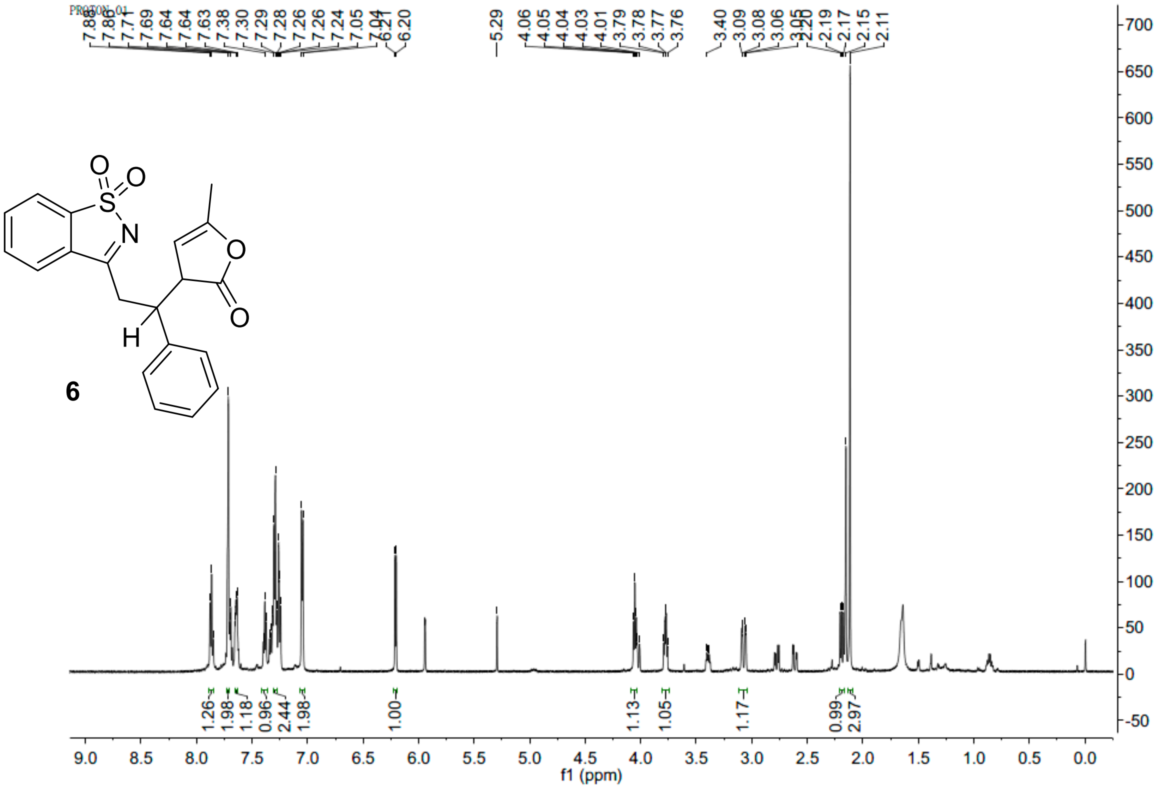
PROTON_01
7.86
7.86
7.71
7.69
7.64
7.64
7.63
7.38
7.30
7.29
7.28
7.26
7.26
7.24
7.05
7.04
6.21
6.20
5.29
4.06
4.05
4.04
4.03
4.01
3.79
3.78
3.77
3.76
3.40
3.09
3.08
3.06
3.05
2.20
2.19
2.17
2.15
2.11
1.26
1.98
1.18
0.96
2.44
1.98
1.00
1.13
1.05
1.17
0.99
2.97
6
f1 (ppm)

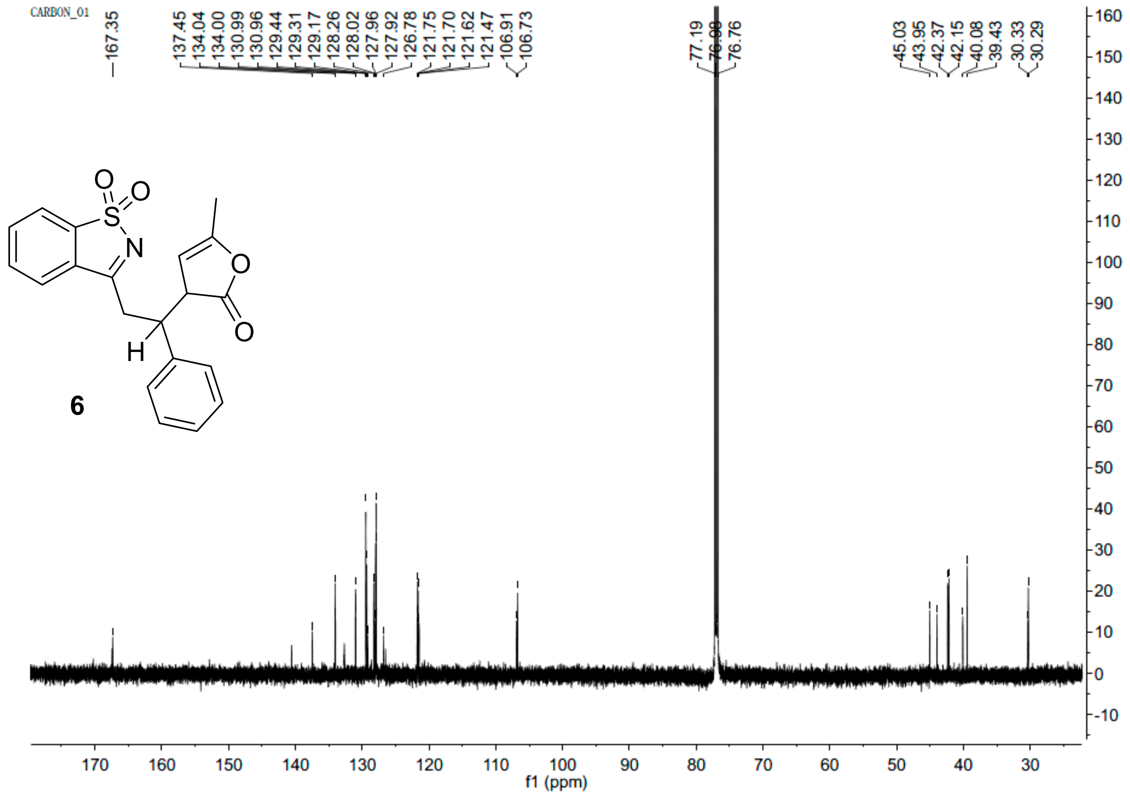
CARBON_01
167.35
137.45
134.04
134.00
130.99
130.96
129.44
129.31
129.17
128.26
128.02
127.96
127.92
126.78
121.75
121.70
121.62
121.47
106.91
106.73
77.19
76.98
76.76
45.03
43.95
42.37
42.15
40.08
39.43
30.33
30.29
6
f1 (ppm)

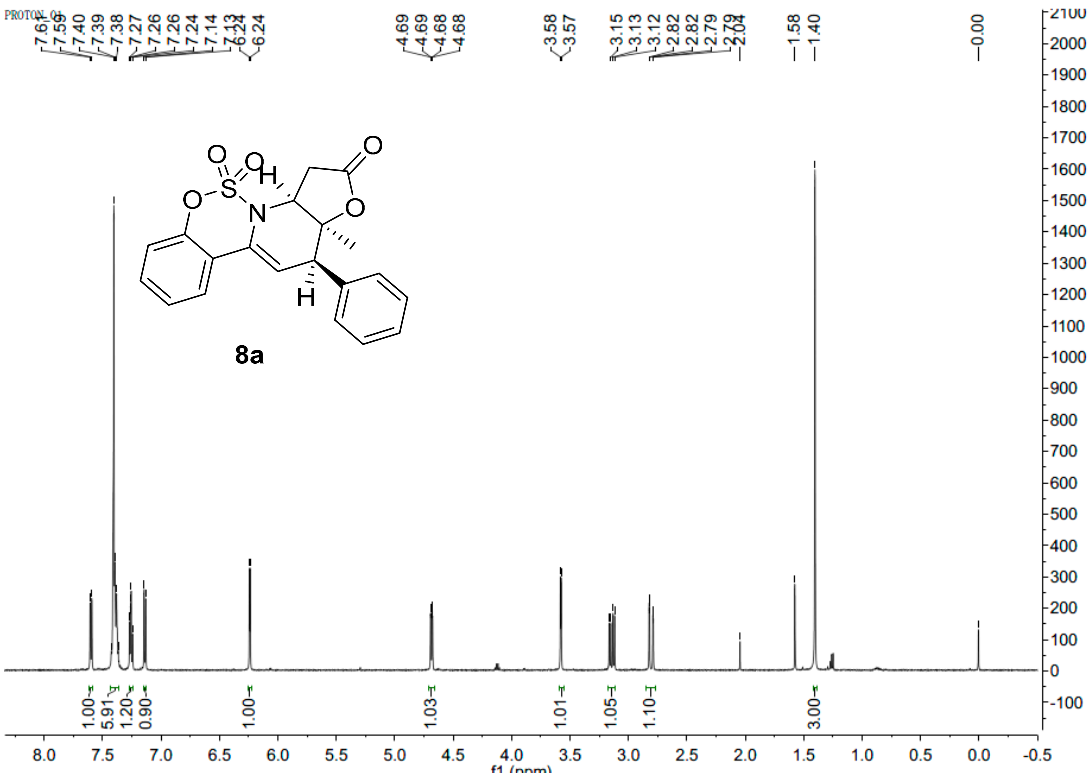
PROTON_01
7.61
7.59
7.40
7.39
7.38
7.27
7.26
7.26
7.24
7.14
7.13
6.24
6.24
4.69
4.69
4.68
4.68
3.58
3.57
3.15
3.13
3.12
2.82
2.82
2.79
2.79
2.04
1.58
1.40
0.00
8a
1.00
5.91
1.20
0.90
1.00
1.03
1.01
1.05
1.10
3.00
f1 (ppm)

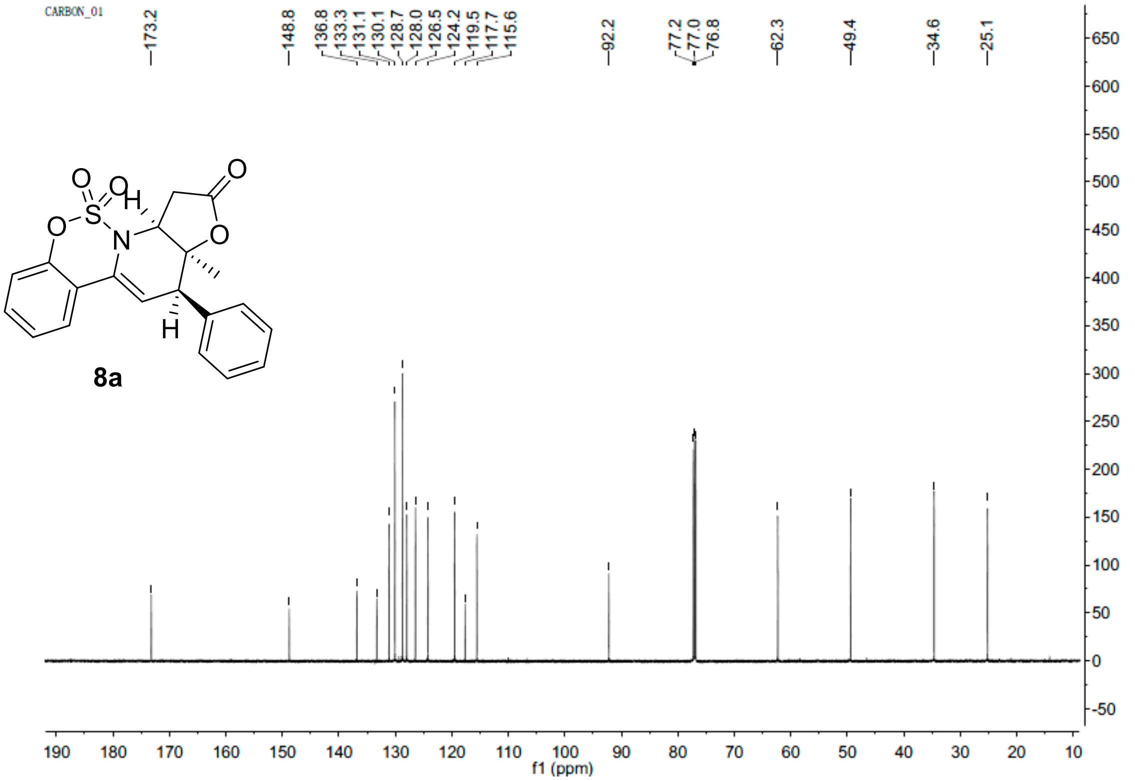
CARBON_01
173.2
148.8
136.8
133.3
131.1
130.1
128.7
128.0
126.5
124.2
119.5
117.7
115.6
92.2
77.2
77.0
76.8
62.3
49.4
34.6
25.1
8a
f1 (ppm)

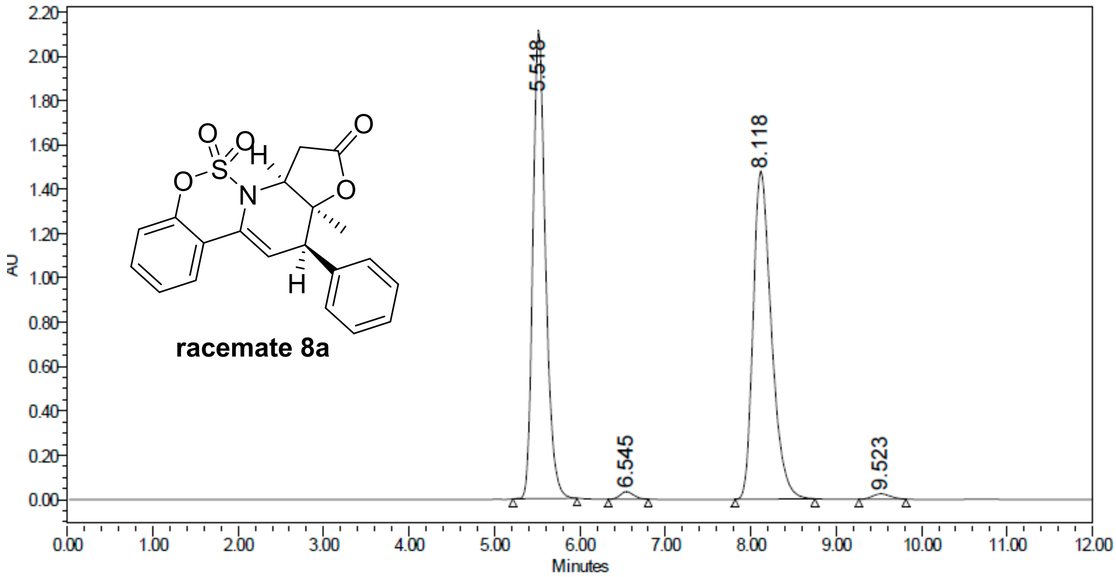

	RT	Area	% Area	Height
1	5.518	21616060	48.75	2109293
2	6.545	375747	0.85	34296
3	8.118	21988883	49.60	1479843
4	9.523	355988	0.80	24360

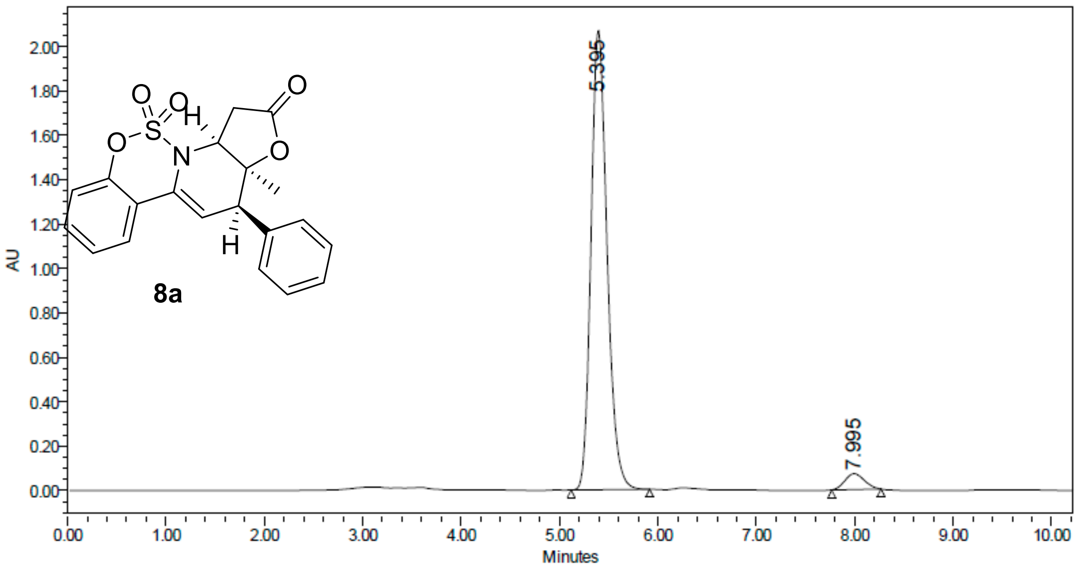

	RT	Area	% Area	Height
1	5.395	23545750	96.00	2072733
2	7.995	982185	4.00	71523

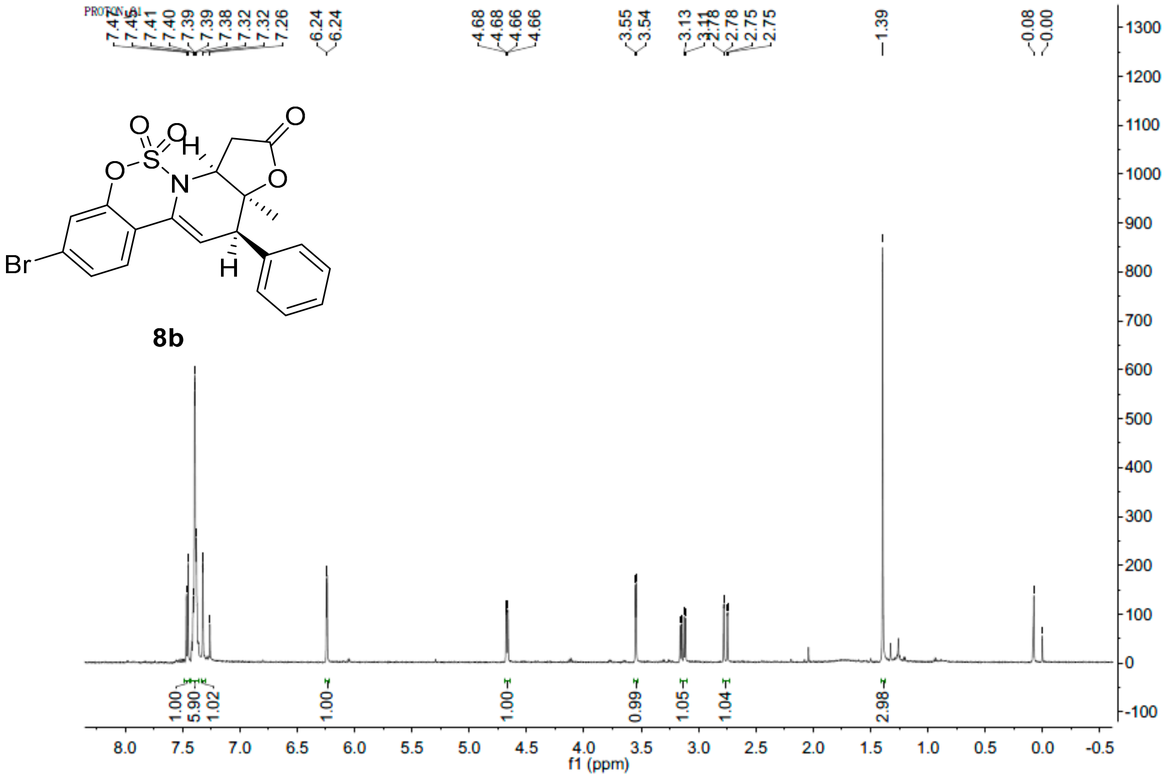
PROTON_01
7.45 7.45 7.41 7.40 7.39 7.39 7.38 7.32 7.32 7.26
6.24 6.24
4.68 4.68 4.66 4.66
3.55 3.54
3.13 3.11 2.78 2.78 2.75 2.75
1.39
0.08 0.00
Br
O
S
N
H
8b
1.00 5.90 1.02 1.00 1.00 0.99 1.05 1.04 2.98
f1 (ppm)

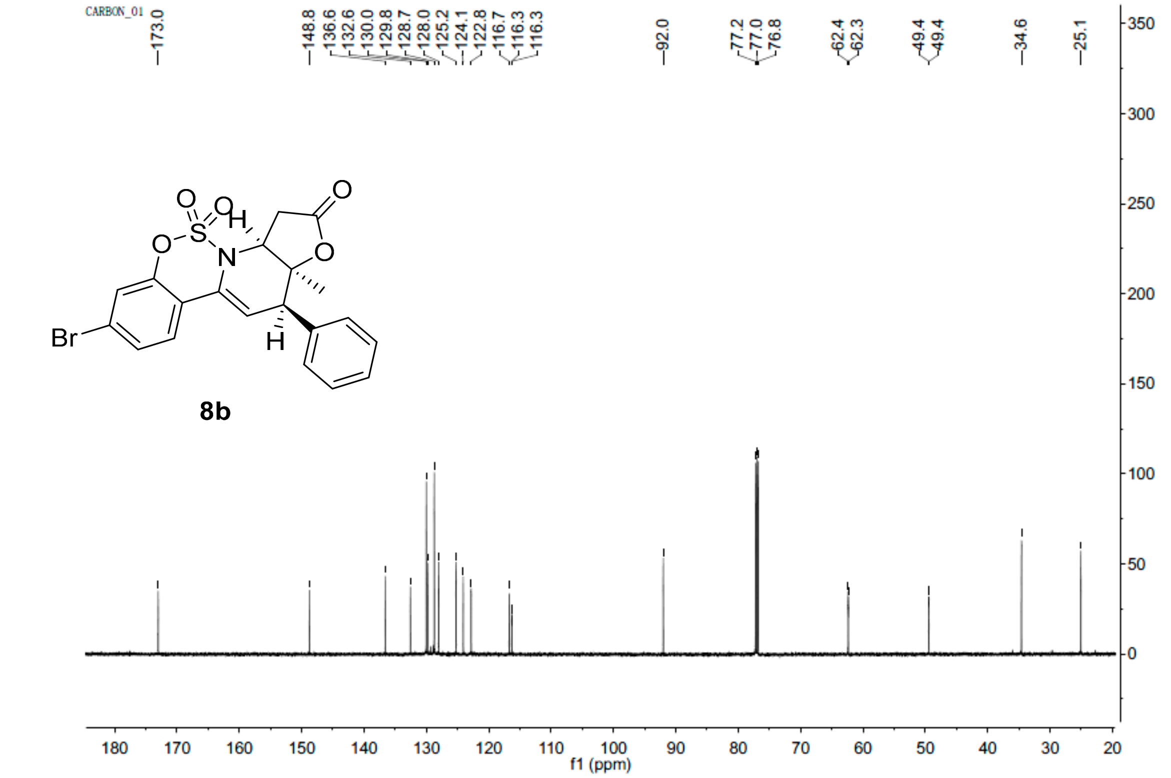
CARBON_01
173.0
148.8 136.6 132.6 130.0 129.8 128.7 128.0 125.2 124.1 122.8 116.7 116.3 116.3
92.0
77.2 77.0 76.8
62.4 62.3
49.4 49.4
34.6
25.1
Br
O
S
N
H
8b
f1 (ppm)

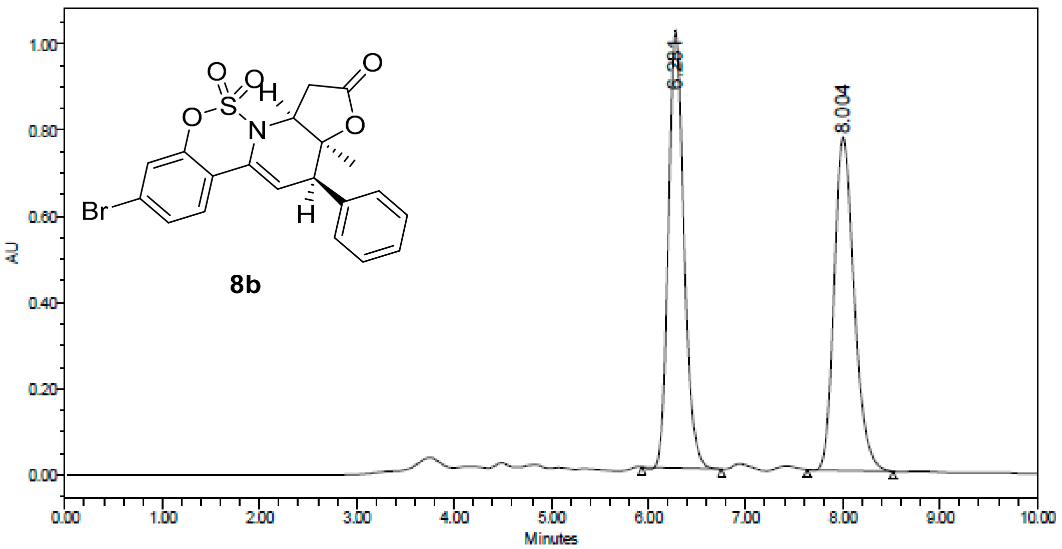

	RT	Area	% Area	Height
1	6.281	11217136	50.29	1016752
2	8.004	11087754	49.71	774545

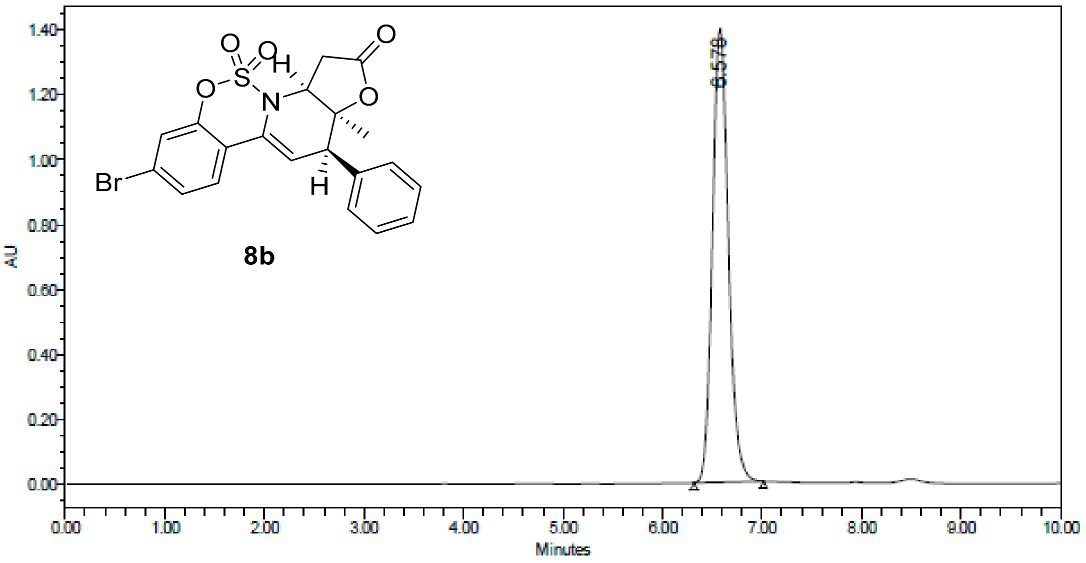

	RT	Area	% Area	Height
1	6.578	15501566	100.00	1401323

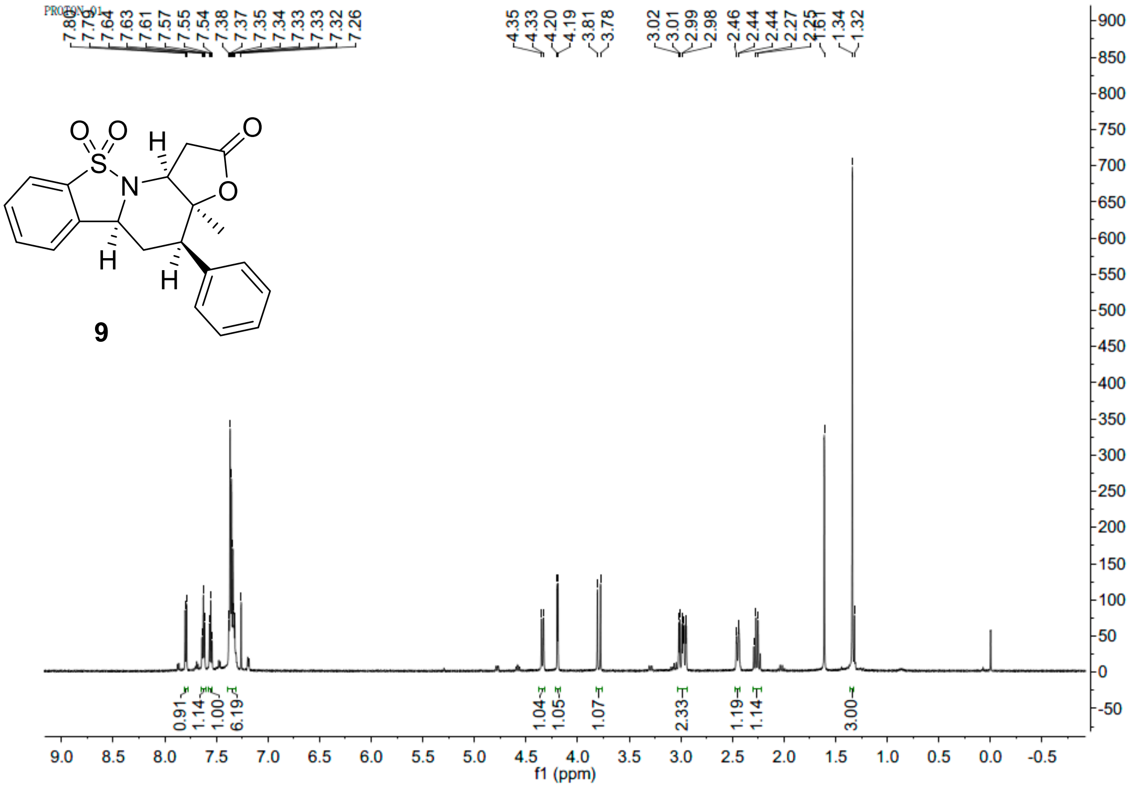
PROTON_01
7.80 7.79 7.64 7.63 7.61 7.57 7.55 7.54 7.38 7.37 7.35 7.34 7.33 7.33 7.32 7.26
4.35 4.33 4.20 4.19 3.81 3.78
3.02 3.01 2.99 2.98 2.46 2.44 2.27 2.25 1.61 1.34 1.32
9
0.91 1.14 1.00 6.19 1.04 1.05 1.07 2.33 1.19 1.14 3.00
f1 (ppm)

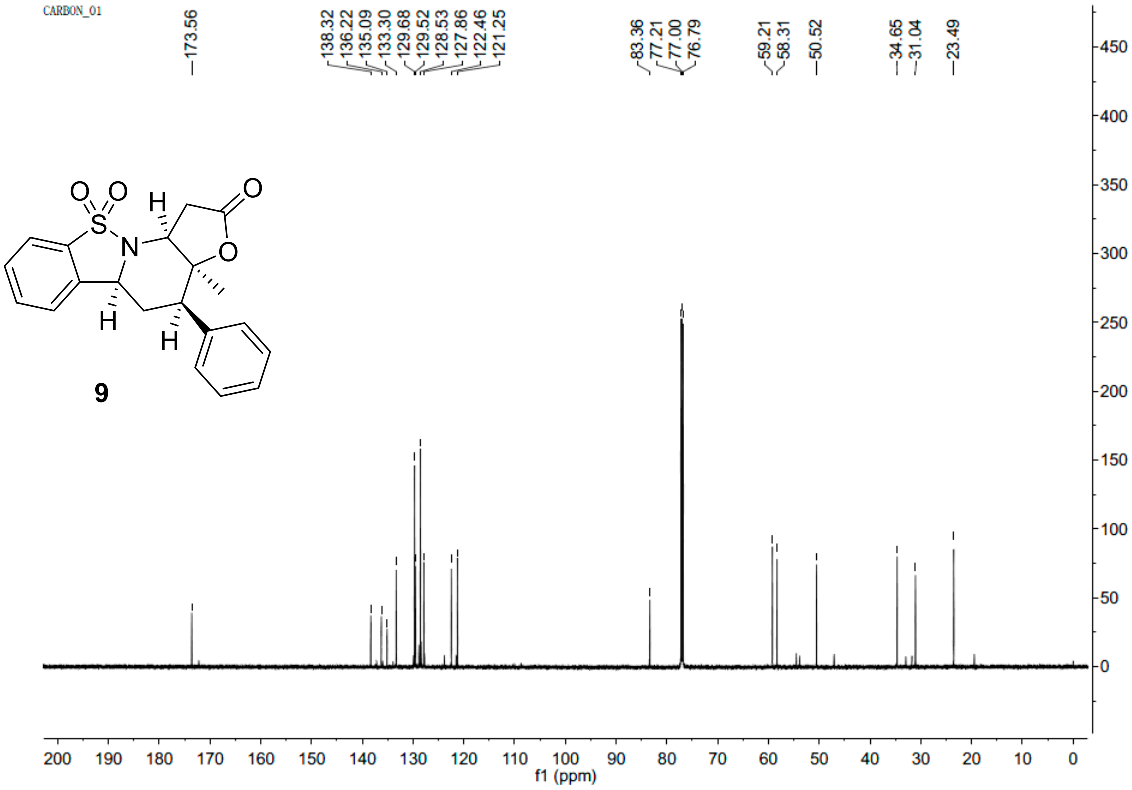
CARBON_01
173.56
138.32 136.22 135.09 133.30 129.68 129.52 128.53 127.86 122.46 121.25
83.36 77.21 77.00 76.79
59.21 58.31 50.52
34.65 31.04 23.49
9
f1 (ppm)

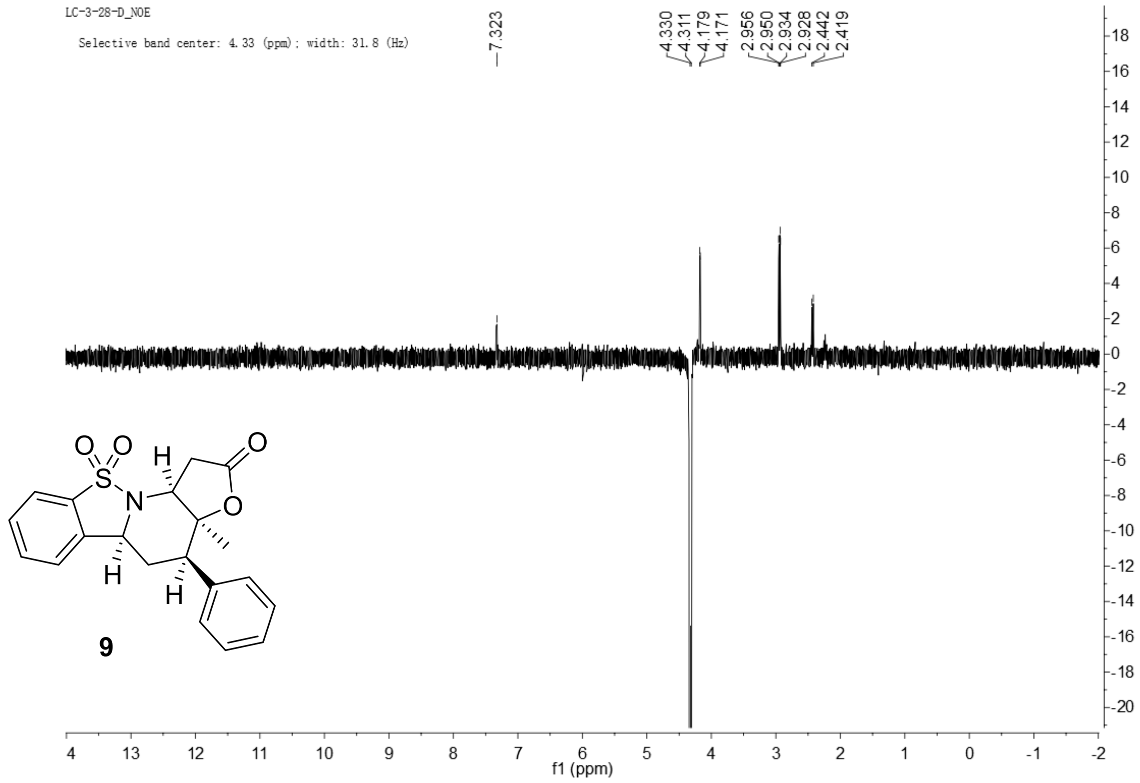
LC-3-28-D_NOE
Selective band center: 4.33 (ppm); width: 31.8 (Hz)
7.323
4.330
4.311
4.179
4.171
2.956
2.950
2.934
2.928
2.442
2.419
f1 (ppm)
9

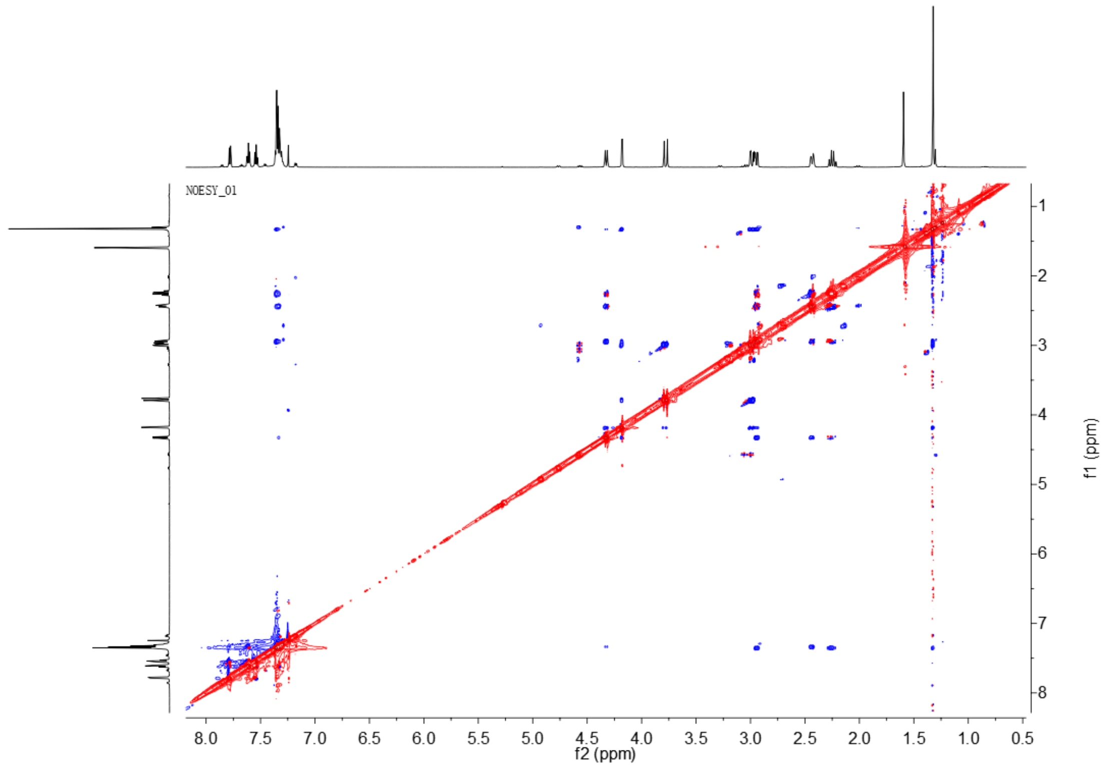
NOESY_01
f2 (ppm)
f1 (ppm)

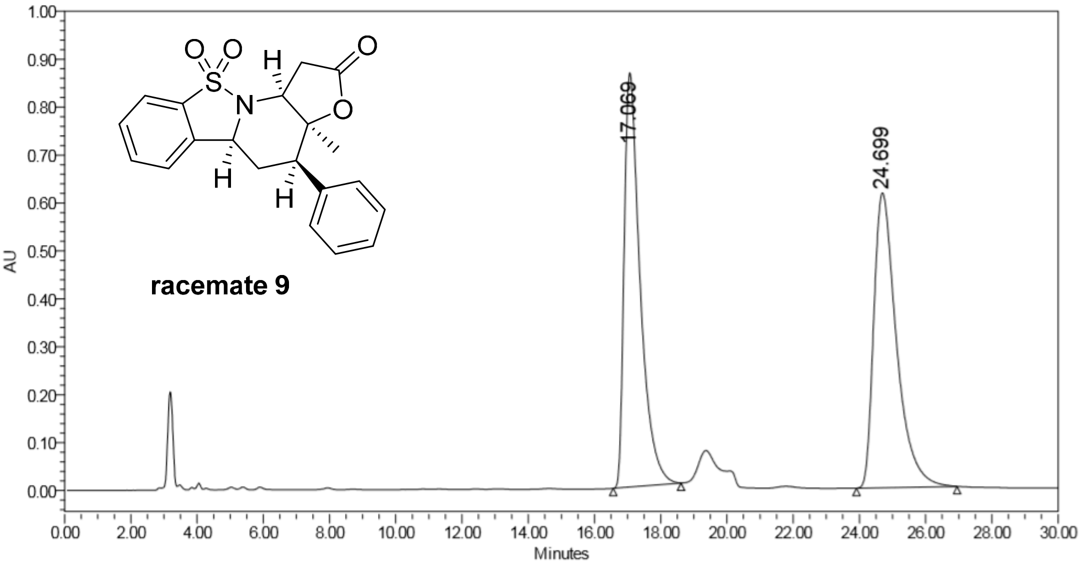

	RT	Area	% Area	Height
1	17.069	28990104	49.74	864370
2	24.699	29298930	50.26	614975

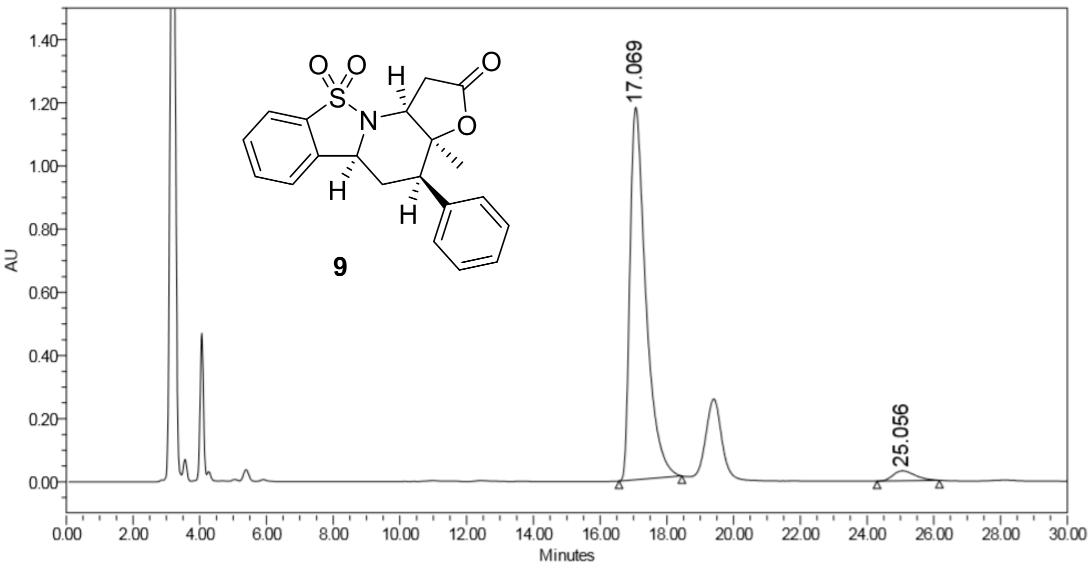

	RT	Area	% Area	Height
1	17.069	39112459	96.40	1179202
2	25.056	1460920	3.60	31697
